# Rising global burden of anxiety disorders among adolescents and young adults: trends, risk factors, and the impact of socioeconomic disparities and COVID-19 from 1990 to 2021

**DOI:** 10.3389/fpsyt.2024.1489427

**Published:** 2024-11-26

**Authors:** Fengsai Bie, Xiaoling Yan, Jie Xing, Leilei Wang, Yang Xu, Guan Wang, Qian Wang, Jinyu Guo, Jing Qiao, Zhenzhen Rao

**Affiliations:** ^1^ National Center for Occupational Safety and Health, National Health Commission of the People’s Republic of China, Beijing, China; ^2^ Institute of Medical Information, Chinese Academy of Medical Sciences and Peking Union Medical College, Beijing, China; ^3^ School of Public Health, Shandong Second Medical University, Weifang, China; ^4^ Office of Education and Training (Graduate School), Chinese Center for Disease Control and Prevention, Beijing, China; ^5^ Dalla Lana School of Public Health, University of Toronto, Toronto, ON, Canada; ^6^ Department of Psychology, School of Humanities, Tongji University, Shanghai, China; ^7^ Educational Department, Peking University First Hospital, Beijing, China; ^8^ Department of Epidemiology and Health Statistics, Xiangya School of Public Health, Central South University, Changsha, China

**Keywords:** anxiety disorders, adolescents, young adults, Global Burden of Disease, Joinpoint regression

## Abstract

**Background:**

Anxiety disorders are among the most prevalent mental health conditions globally, particularly affecting adolescents and young adults (10-24 years), and causing substantial psychological and social impairments. This study analyzed changes in the incidence, prevalence, and disability-adjusted life years (DALYs) of anxiety disorders in this age group from 1990 to 2021, emphasizing the impact of socioeconomic disparities and the COVID-19 pandemic, particularly post-2019.

**Methods:**

Utilizing the Global Burden of Disease(GBD) 2021 data from 204 countries, this study evaluated global trends in anxiety disorders among adolescents and young adults. Conducted between May 16 and August 1, 2024, it assessed prevalence, incidence, DALYs, and estimated annual percentage changes (EAPCs) from 1990 to 2021. Joinpoint regression identified significant shifts in incidence rates, with key risk factors, especially bullying victimization,examined. The analysis was stratified by region, country, age group, sex, and Socio-Demographic Index (SDI).

**Results:**

From 1990 to 2021, the global incidence of anxiety disorders among those aged 10-24 years increased by 52%, particularly in the 10-14 age group and post-2019. Females showed higher prevalence rates than males, and DALYs rose notably among the 20-24-year-olds. Regions with middle SDI reported the highest incidence and prevalence, whereas high SDI regions experienced the largest increases. India had the highest number of cases, while Mexico saw the greatest rise. A gradual decline in incidence was noted until 2001, followed by a slow increase, with a sharp rise from 2019 to 2021. Bullying victimization was a significant risk factor, especially in regions with a high anxiety disorder burden.

**Conclusion:**

The rising incidence of anxiety disorders among adolescents and young adults over the past 30 years reflects the increasing global mental health burden. Socioeconomic factors, particularly in middle SDI regions, and the impact of the COVID-19 pandemic have exacerbated this trend. Effective, targeted interventions focusing on early prevention and community-based mental health management are urgently needed to mitigate the long-term impact on young populations globally.

## 1 Introduction

Anxiety disorders are among the most common mental health conditions globally, significantly impacting the psychological health and social functioning of adolescents and young adults.These disorders typically manifest between the ages of 10 and 24, leading to severe health losses and role impairments (1–4). With increasing globalization and social pressures, the prevalence and socioeconomic impact of anxiety disorders are escalating. Approximately 2.08% of global health expenditure is allocated to treating anxiety disorders, underscoring their profound effect on individual health and the global economy (5). Given their early onset and chronic nature, anxiety disorders place a heavy burden on young people, especially those aged 10 to 24, leading to significant impairments in social and functional capacities. The etiology of anxiety disorders is multifactorial, involving genetic, biological, and environmental factors (6), and their early onset and chronic nature, particularly in individuals aged 10 to 24, underscores the need for a comprehensive understanding of how sociocultural and economic factors influence their global prevalence (7, 8).

The Global Burden of Disease (GBD) study has been instrumental in providing valuable insights into the incidence, prevalence, and disability burden of anxiety disorders across various regions. These studies reveal significant global disparities, often reflecting differences in healthcare infrastructure, social support systems, and levels of economic development (9–11). For instance, Baxter’s analysis of GBD 2010 data identified anxiety disorders as the sixth leading cause of global disability, highlighting their substantial impact on health systems (12). Further research has explored the specific social and age-related factors that contribute to the burden of anxiety disorders, particularly in children and adolescents. Studies by Erskine et al. (13) highlighted the high prevalence of anxiety disorders in younger populations, while Xiong, Yang, and Javaid et al. (14–17) examined Disability-Adjusted Life Years (DALYs) trends globally and regionally, identifying demographic factors that drive these burdens.

This study aims to address the following specific research questions: (1) What are the global trends in the incidence, prevalence, and DALYs of anxiety disorders among adolescents and young adults aged 10-24 years from 1990 to 2021? (2) How do these trends vary by region, country, age group, sex, and socioeconomic status as measured by the SDI? (3) What is the impact of significant risk factors, particularly bullying victimization, on the burden of anxiety disorders in this age group?

To address these questions, this study utilizes GBD data from 204 countries and regions over the period from 1990 to 2021. By analyzing the incidence, prevalence, and DALYs of anxiety disorders, this research seeks to enhance the understanding of the global burden of these disorders and provide a robust foundation for public health decision-making. The findings will also support the development of targeted interventions and preventive measures tailored to younger populations, particularly in regions with significant socioeconomic disparities.

## 2 Methods

### 2.1 Study design and ethics

This cross-sectional study was approved by the National Center for Occupational Safety and Health, National Health Commission. The ethics committee of the National Center for Occupational Safety and Health agreed to waive informed consent, as the study only involved data analysis without identifiable personal information. Data for adolescents and young adults aged 10–24 years were collected using the Global Health Data Exchange query tool created by GBD collaborators. The GHDx is a comprehensive online data repository that provides access to global health and demographic data, including data across different regions, genders, causes, and disease evaluation metrics such as incidence, prevalence, and DALYs.

### 2.2 Case definitions

To ensure consistency in measurement, we utilized case definitions for anxiety disorders as established within the GBD framework, including the diagnostic instruments used for case identification. These definitions comply with the criteria set forth in the fourth edition text revision of the Diagnostic and Statistical Manual of Mental Disorders (DSM-IV-TR) (18) and the tenth edition of the International Classification of Diseases (ICD-10) (19). Anxiety disorders encompass experiences of intense fear and distress, usually accompanied by other physiological symptoms. We employed validated diagnostic tools, such as structured clinical interviews and standardized self-report questionnaires, to ensure accurate and consistent identification of cases across different studies. Within GBD, anxiety disorders are modeled as a single cause for “any” anxiety disorder to prevent the double-counting of individuals who meet the criteria for multiple anxiety disorders (DSM-IV-TR: 300.0-300.3, 208.3, 309.21, 309.81; ICD-10: F40-42, F43.0, F43.1, F93.0-93.2, F93.8).

### 2.3 Study population and data collection

In our analysis of the Global Burden of Disease Study 2021, we accessed repeated cross-sectional data from the Global Health Data Exchange platform, covering the global burden of 371 diseases and injuries and 88 risk factors, including anxiety disorder data. The data span 21 regions and 204 countries and territories from 1990 to 2021. The study population focuses on adolescents and young adults aged 10-24 years, specifically assessing the burden of anxiety disorders within this age group. We extracted information on the incidence, prevalence, and DALYs for anxiety disorders in this population, evaluating their counts and rates. The data were accessed and downloaded via the Global Health Data Exchange (GHDx) platform (20) (http://ghdx.healthdata.org/gbd-results-tool). Additionally, we obtained SDI data to assess the impact of socioeconomic factors on disease burden. This cross-sectional study follows the STROBE (Strengthening the Reporting of Observational Studies in Epidemiology) guidelines for reporting observational studies.

### 2.4 Statistical analysis

#### 2.4.1 Global and regional burden analysis

To analyze the global distribution and regional differences in the burden of anxiety disorders among adolescents and young adults (10-24), we generated global maps and regional comparative analyses. The data were aggregated by geographical regions as defined by the GBD study, and maps were created using R with the `ggplot2` and `sf` packages to visualize the distribution of disease burden.

#### 2.4.2 Population analysis

Population-level analyses were conducted to explore the distribution of anxiety disorders among adolescents and young adults (10-24) across different demographic groups, including age, sex, and specific subpopulations. The data were stratified by age groups three (10-15 years, 16-19 years, 20-24 years) for both males and females. Statistical analyses were performed using R, and results were visualized using the `ggplot2` package.

#### 2.4.3 Sociodemographic Index analysis

The Socio-demographic Index (SDI) is a composite measure used to assess the development status of a region, incorporating three key indicators: income per capita, average educational attainment, and total fertility rate. These factors collectively capture the socio-economic and demographic context, which are critical in shaping health outcomes, including the burden of anxiety disorders (20, 21). In this study, we analyzed the relationship between SDI and the burden of anxiety disorders among adolescents and young adults (ages 10-24) by calculating SDI-specific disease rates. SDI categories (low, low-middle, middle, high-middle, and high) were used to compare disease burden across varying levels of socio-economic development. Data manipulation and visualization were performed using the `dplyr` and `ggplot2 `packages in R.

#### 2.4.4 Temporal trend analysis

Temporal trends in the incidence of anxiety disorders among adolescents and young adults (10-24) from 1990 to 2021 were assessed using Joinpoint regression analysis. The analysis was performed using the `Joinpoint` R package, which identifies significant changes in trends over time. The annual percentage change (APC) and average annual percentage change (AAPC) were calculated, with 95% confidence intervals (CIs) used to determine the statistical significance of the trends.

## 3 Results

### 3.1 Global trends

#### 3.1.1 Incidence

**Figure 1:**
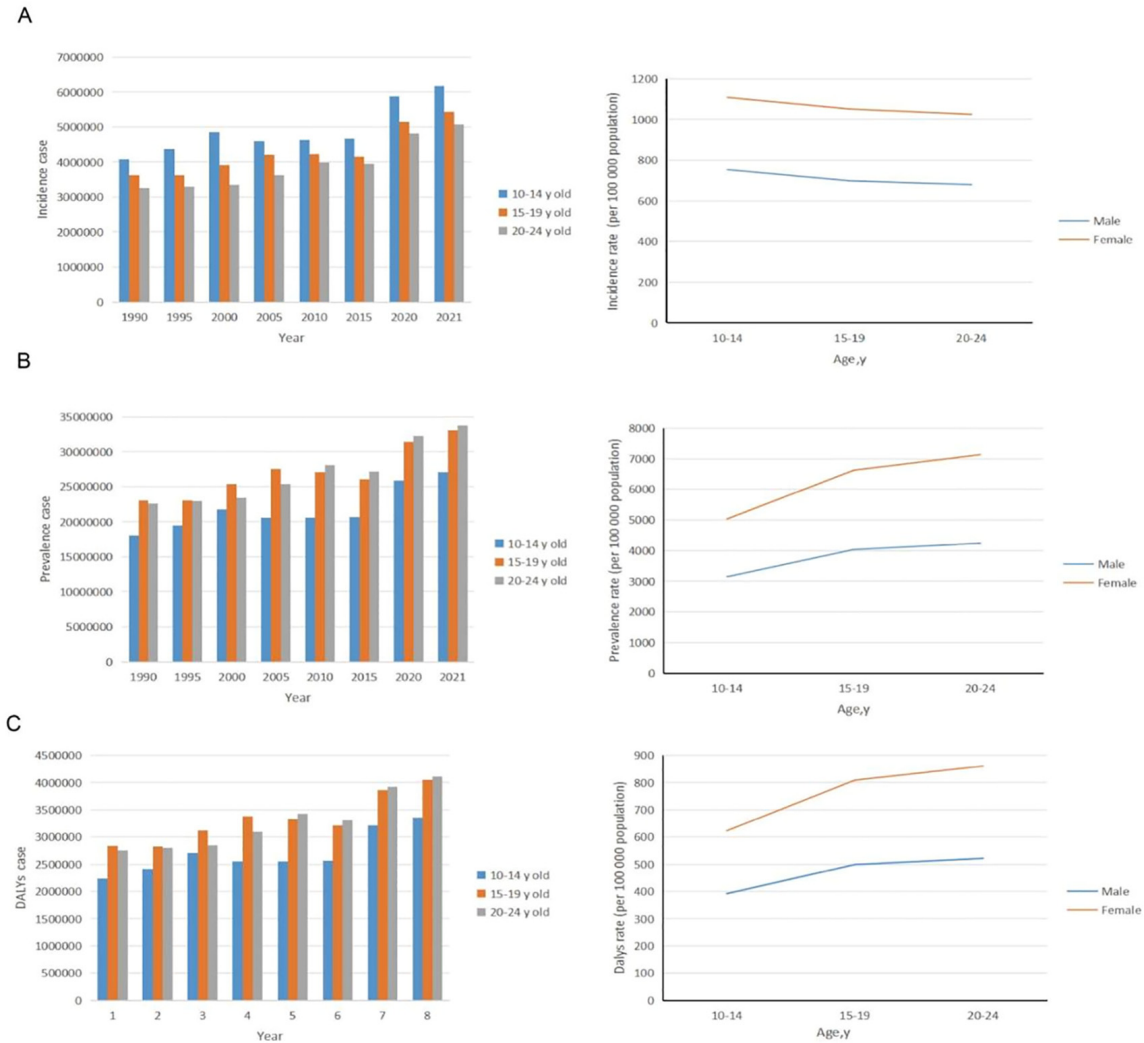
Trends in incidence, prevalence, and disability-adjusted life-years (DALYs) of anxiety disorders among adolescents and young adults aged 10–24 years from 1990 to 2021. **(A)** Trends in incident cases and incident rate. **(B)** Trends in prevalence cases and prevalence rate. **(C)** Trends in DALYs cases and DALYs rate.

The study included 399 121 466 adolescents and young adults aged 10–24 years, comprising 163 612 758 males (40.99%) and 235 508 708 females (59.01%). In 2021, there were 16 670 879 (95% UI: 12 024 089 to 22 032 683) incident cases of anxiety disorders among adolescents and young adults aged 10–24 years globally. From 1990 to 2021, the global incident cases of anxiety disorders in this age group increased by 52.00% (95% UI: 49.00% to 56.00%), with the incidence rate rising from 708.02 (95% UI: 511.55 to 939.28) per 100 000 in 1990 to 883.10 (95% UI: 636.95 to 1 167.13) per 100 000 in 2021. The Estimated Annual Percentage Change (EAPC) was 0.20 (95% CI: 0.01 to 0.39). During this period, the incidence of anxiety disorders increased across all age groups, with the highest increase observed in the 20-24 age group (28.33%) and the smallest increase in the 10-14 age group (21.58%). Despite having the smallest increase, the 10-14 age group still had the highest incidence rates in both 1990 and 2021, at 760.96 and 925.07, respectively. In 2021, the incidence rate of anxiety disorders was generally higher in females than in males across all age groups (**Table 1** and **Figure 1A**).

**Table 1:** Incidence of anxiety disorders in adolescents and young adults aged 10–24 years between 1990 and 2021 at the global and regional level.

Location	Rate per 100 000 (95% UI)					
	1990		2021		1990-2021	
	Incident cases	Incidence rate	Incident cases	Incidence rate	Cases change	EAPC^a^
Global	10954492.412 (7914602.800 - 14532409.404)	708.023 (511.546 - 939.275)	16670879.618 (12024089.386 - 22032683.605)	883.097 (636.945 - 1167.125)	0.783 (0.723 - 0.850)	0.202 (0.013 - 0.393)
SDI						
High	1742934.855 (1268323.308 - 2295511.065)	889.900 (647.575-1172.032)	2274833.619 (1640893.251-2991722.642)	1225.831 (884.222-1612.138)	0.452 (0.378 - 0.526)	0.465 (0.250 - 0.681)
High middle	2049548.972 (1496239.968 - 2706818.421)	722.220 (527.245 - 953.829)	2084463.642 (1533623.258-2780787.447)	922.819 (678.954-1231.090)	0.394 (0.305 - 0.488)	0.133 (-0.101 - 0.367)
Middle SDI	3847856.814 (2777918.389 - 5064049.155)	701.106 (506.156 - 922.705)	5018673.637 (3600931.747-6634075.923)	907.955 (651.463-1200.205)	0.748 (0.649 - 0.856)	0.232 (0.033 - 0.432)
Low middle	2282198.493 (1626631.181 - 3065960.789)	630.942 (449.702 - 847.623)	4390668.868 (3129600.665-5882209.782)	794.339 (566.192-1064.181)	1.160 (1.033 - 1.277)	0.345 (0.137 - 0.554)
Low SDI	1022114.243 (727873.176-1379168.376)	656.630 (467.603-886.010)	2888375.271 (2061185.504-3865352.723)	781.875 (557.957-1046.340)	1.748 (1.595 - 1.907)	0.242 (0.091 - 0.392)
Regions						
Andean Latin America	117924.034 (80370.705-168534.516)	957.924 (652.870-1369.045)	250938.886 (160223.932-373069.243)	1453.516 (928.067-2160.933)	1.128 (0.787 - 1.482)	0.518 (0.138 - 0.900)
Australasia	57849.487 (38987.019-80866.764)	1202.411 (810.352-1680.830)	78420.540 (51000.633-111379.978)	1366.886 (888.951-1941.376)	0.356 (0.103 - 0.679)	0.308 (0.155 - 0.461)
Caribbean	85407.241 (59211.427-120107.608)	799.785 (554.478-1124.732)	116190.202 (78428.069-170008.352)	1025.735 (692.368-1500.845)	0.36 (0.207 - 0.525)	0.276 (0.086 - 0.467)
Central Asia	85538.324 (59230.238-120359.589)	431.331 (298.671-606.919)	123138.984 (84104.186-177976.337)	556.500 (380.091-804.326)	0.44 (0.288 - 0.62)	0.193 (-0.024 - 0.410)
Central Europe	181810.393 (128407.326-247569.008)	622.674 (439.776-847.888)	163108.426 (113710.141-224368.513)	899.351 (626.978-1237.129)	-0.103 (-0.166 - -0.038)	0.344 (0.062 - 0.627)
Central Latin America	374947.417 (267445.427-500549.421)	691.077 (492.937-922.578)	671585.471 (470452.618-912400.378)	1032.682 (723.405-1402.978)	0.791 (0.646 - 0.955)	0.738 (0.470 - 1.006)
Central Sub-Saharan Africa	125262.036 (86196.117-175923.841)	723.729 (498.017-1016.439)	368599.553 (247670.127-542381.594)	820.132 (551.064-1206.796)	1.943 (1.448 - 2.567)	0.172 (0.043 - 0.302)
East Asia	2438390.226 (1752795.902-3202210.157)	655.165 (470.955-860.394)	1701655.511 (1265938.351-2226837.495)	700.239 (520.939-916.354)	-0.302 (-0.352 - -0.249)	-0.432 (-0.657 - -0.208)
Eastern Europe	315622.938 (227583.768-415460.207)	668.288 (481.877-879.679)	323485.232 (234530.606-426387.927)	980.413 (710.811-1292.288)	0.025 (-0.034 - 0.088)	0.316 (0.029 - 0.605)
Eastern Sub-Saharan Africa	452490.550 (317307.640-618439.864)	729.429 (511.510-996.945)	1293468.337 (888587.399-1768224.012)	889.371 (610.980-1215.806)	1.859 (1.663 - 2.061)	0.173 (0.012 - 0.335)
High-income Asia Pacific	258593.533 (185622.607-338850.037)	613.826 (440.614-804.332)	193764.487 (138330.300-258348.323)	742.252 (529.901-989.653)	-0.251 (-0.315 - -0.182)	-0.015 (-0.192 - 0.163)
High-income North America	588366.770 (415672.053-777801.499)	961.805 (679.500-1271.474)	984972.371 (676343.702-1299586.691)	1381.850 (948.865-1823.233)	0.674 (0.57 - 0.775)	0.438 (0.141 - 0.736)
North Africa and Middle East	1113940.960 (834250.430-1469423.013)	1022.846 (766.027-1349.257)	2061401.739 (1486146.766-2797949.358)	1270.141 (915.695-1723.968)	0.851 (0.703 - 0.99)	0.290 (0.097 - 0.484)
Oceania	15331.071 (10941.975-21178.976)	732.873 (523.061-1012.421)	33796.073 (22417.795-49582.050)	838.118 (555.945-1229.598)	1.204 (0.773 - 1.693)	0.087 (-0.010 - 0.185)
South Asia	1792408.401 (1259076.684-2390415.504)	535.896 (376.440-714.688)	3478267.526 (2378342.176-4619814.111)	661.430 (452.267-878.507)	0.941 (0.824 - 1.06)	0.375 (0.096 - 0.656)
Southeast Asia	929074.457 (662089.598-1234337.025)	626.204 (446.254-831.953)	1454024.049 (1027456.895-1970472.375)	850.260 (600.819-1152.261)	0.565 (0.487 - 0.655)	0.318 (0.103 - 0.534)
Southern Latin America	131613.632 (97479.916-176935.325)	994.305 (736.434-1336.698)	202068.494 (133411.162-289893.142)	1317.569 (869.895-1890.221)	0.535 (0.245 - 0.859)	0.231 (-0.009 - 0.471)
Southern Sub-Saharan Africa	121172.246 (86799.843-160473.282)	709.288 (508.087-939.339)	215811.642 (152930.383-289589.206)	989.303 (701.048-1327.506)	0.781 (0.634 - 0.951)	0.327 (0.073 - 0.581)
Tropical Latin America	479980.603 (344154.182-629408.545)	1002.894 (719.092-1315.116)	802552.797 (554622.704-1082144.227)	1586.806 (1096.599-2139.614)	0.672 (0.533 - 0.809)	0.836 (0.583 - 1.090)
Western Europe	915845.010 (657805.487-1217656.502)	1114.160 (800.245-1481.325)	1050409.843 (756577.927-1393581.551)	1457.376 (1049.703-1933.505)	0.147 (0.071 - 0.24)	0.434 (0.212 - 0.655)
Western Sub-Saharan Africa	372923.082 (271667.064-490755.550)	623.161 (453.961-820.062)	1103219.455 (805534.919-1446126.130)	683.677 (499.199-896.180)	1.958 (1.815 - 2.106)	0.162 (0.059 - 0.265)

^a^EAPC is expressed as 95% CIs.

#### 3.1.2 Prevalence

Over the past 30 years, the global prevalence of anxiety disorders among adolescents and young adults aged 10–24 years increased from 4 120.60 (95% UI: 3 209.06 to 5 204.47) per 100 000 in 1990 to 4 976.61 (95% UI: 3 808.62 to 6 371.49) per 100 000 in 2021, with an EAPC of 0.16 (95% CI: -0.01 to 0.32) (**Supplementary Table 1**). The prevalence increased across all age groups, with the largest increase observed in the 20–24 years age group (23.16%) and the smallest in the 15–19 years age group (19.32%). The age groups with the highest prevalence in 1990 and 2021 were 15–19 years (23 053 160.15) and 20–24 years (33 826 064.31), respectively. In 2021, the prevalence of anxiety disorders was generally higher in females than in males. The highest prevalence rates were observed in the 20–24 years age group for both males (4 243.16; 95% UI: 3 093.26 to 5 660.23) per 100 000 and females (7 132.57; 95% UI: 5 270.13 to 9 395.55) per 100 000 (**Figure 1B**).

#### 3.1.3 DALYs

From 1990 to 2021, the global rate of Disability-Adjusted Life Years (DALYs) due to anxiety disorders among adolescents and young adults aged 10–24 years increased by 104.85% (from 505.59; 95% UI: 318.35 to 739.73, to 610.44; 95% UI: 385.28 to 895.81 per 100 000) in 2021, with an EAPC of 0.16 (95% CI: -0.01 to 0.32) (**Supplementary Table 1**). The DALY rate increased across all age groups, with the largest increase observed in the 20–24 years age group (23.08%). The groups with the highest number of DALYs in 1990 and 2021 were the 15–19 years group (2 831 155.67) and the 20–24 years group (4 111 483.66), respectively. In 2021, the DALY rate due to anxiety disorders was generally higher in females than in males across all age groups. The 10–14 years age group had the lowest proportion of anxiety disorder-related DALYs, which increased with age for both genders (**Figure 1C**).

### 3.2 SDI regional trends

#### 3.2.1 Incidence

**Figure 2:**
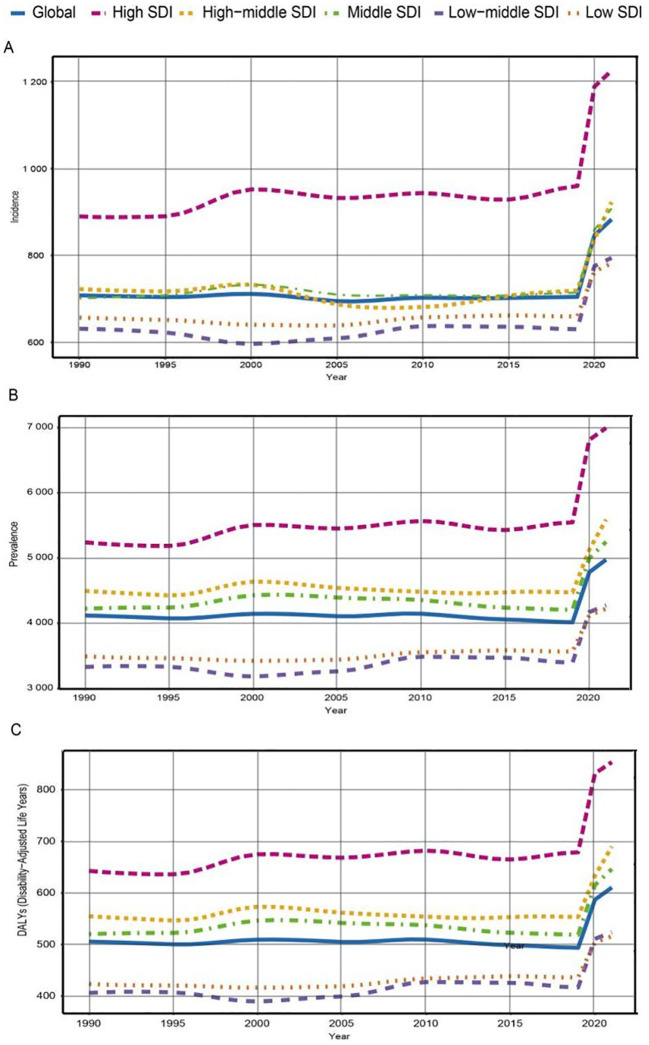
Trends in incidence, prevalence, and disability-adjusted life-years (DALYs) rates of anxiety disorder among adolescents and young adults aged 10–24 years in 5 sociodemographic index (SDI) regions from 1990 to 2021. **(A)** Incidence rate. **(B)** Prevalence rate. **(C)** DALYs rate.

In 2021, the Middle SDI region reported the highest number of anxiety disorder cases among adolescents and young adults aged 10–24 years (5 018 673; 95% UI: 3 600 931 to 6 634 076). Conversely, the High-middle SDI region had the lowest number of cases (2 084 464; 95% UI: 1 533 623 to 2 780 787). The greatest increase in the incidence of anxiety disorders among this age group was observed in the High SDI region (EAPC: 0.47; 95% CI: 0.25 to 0.68) (**Table 1** and **Figure 2A**).

#### 3.2.2 Prevalence

From 1990 to 2021, all five SDI regions exhibited an increasing trend in the prevalence of anxiety disorders among adolescents and young adults aged 10–24 years. In 2021, the Middle SDI region had the highest number of anxiety disorder cases (29 018 755; 95% UI: 22 578 284 to 36 378 105). The High SDI region showed the most significant increase in prevalence (33.45%), while the Low SDI region showed the smallest increase (20.90%). In 2021, the prevalence of anxiety disorders among adolescents and young adults was highest in the High SDI region (6 994.44; 95% UI: 5 440.63 to 8 965.47) and lowest in the Low SDI region (4 223.66; 95% UI: 3 129.09 to 5 665.63). The EAPC was highest in the High SDI region (0.45; 95% CI: 0.25 to 0.64) and lowest in the Middle SDI region (0.18; 95% CI: 0.00 to 0.35) (**Supplementary Table 1** and **Figure 2B**).

#### 3.2.3 DALYs

In 2021, the Middle SDI region had the highest number of DALYs related to anxiety disorders (3 572 197; 95% UI: 2 263 863 to 5 228 392), with a 24.26% increase from 1990 to 2021. The High SDI region experienced the largest increase in DALYs (32.83%), while the Low SDI region experienced the smallest increase (21.82%) (**Supplementary Table 1** and **Figure 2C**).

### 3.4 Geographic regional trends

#### 3.4.1 Incidence

**Figure 3:**
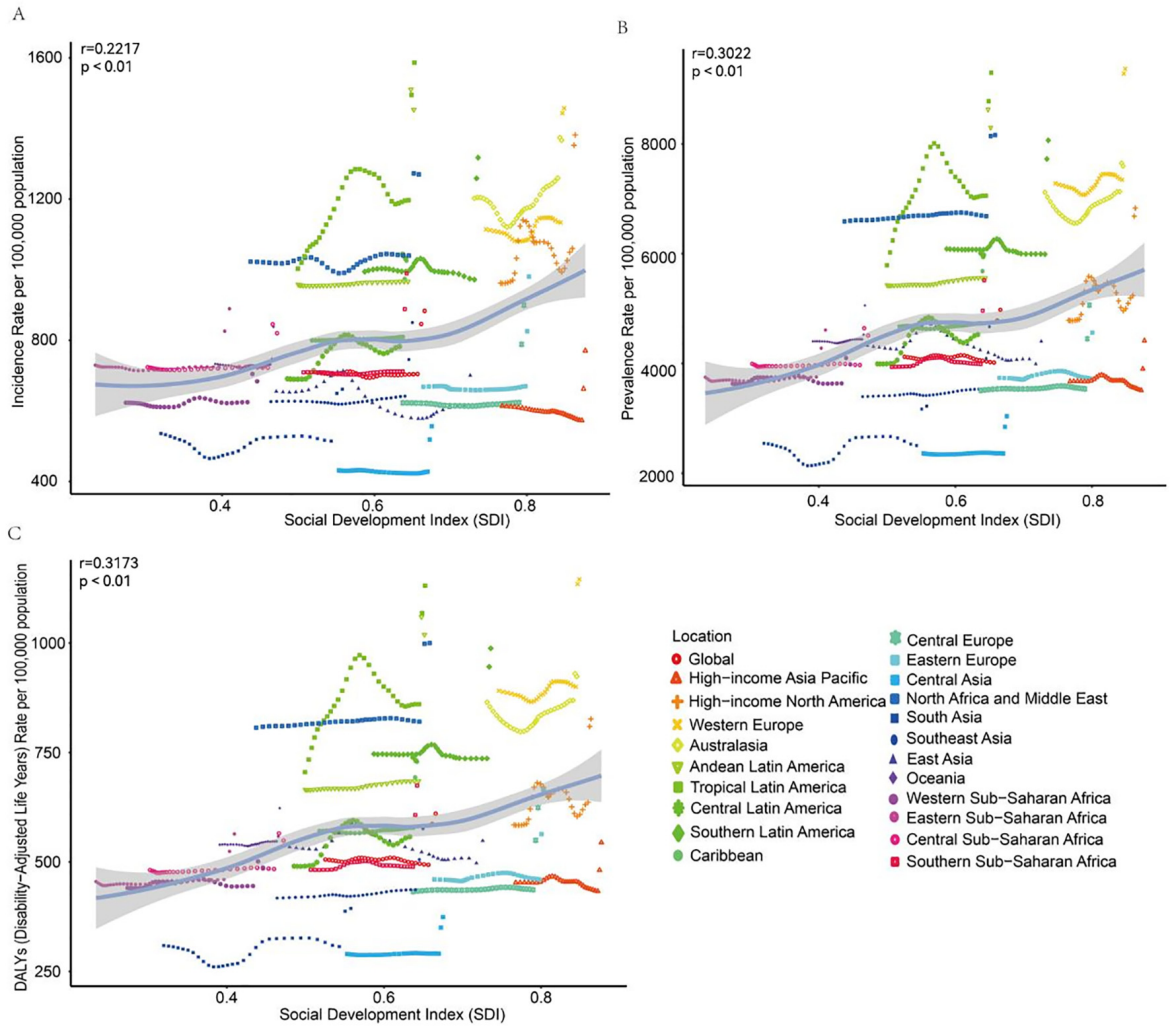
Trends in incidence, prevalence, and disability-adjusted life-years (DALYs) rates for adolescents and young adults aged 10–24 years anxiety disorder in 21 regions from 1990 to 2021. **(A)** Incidence rate. **(B)** Prevalence rate. **(C)** DALYs rate.

In 2021, South Asia had the highest number of anxiety disorder cases among adolescents and young adults aged 10–24 years (3 478 267; 95% UI: 2 378 342 to 4 619 814), while Oceania had the fewest cases (33 796; 95% UI: 22 418 to 49 582). The highest incidence rate was observed in Tropical Latin America (SDI: 0.652) at 1 586.81 cases per 100 000 (95% UI: 1 096.60 to 2 139.61). In contrast, Central Asia (SDI: 0.675) had the lowest incidence rate at 556.50 cases per 100 000 (95% UI: 380.09 to 804.33). From 1990 to 2021, the incidence rate of anxiety disorders in adolescents and young adults increased the most in Tropical Latin America (EAPC: 0.836; 95% CI: 0.583 to 1.090) and decreased the most in East Asia (EAPC: -0.432; 95% CI: -0.657 to -0.208) (**Table 1**). In 2021, the global SDI was 0.666; 14 regions, including Tropical Latin America and Western Europe, had incidence rates higher than the global average (883.097 per 100 000), while 7 regions, including Western Sub-Saharan Africa and South Asia, had lower rates (**Figure 3A**).

#### 3.4.2 Prevalence

In 2021, South Asia had the highest prevalence of anxiety disorders among adolescents and young adults aged 10–24 years (16 949 401; 95% UI: 13 184 205 to 21 187 938), while Oceania had the lowest prevalence (203 887; 95% UI: 136 455 to 297 032). Western Europe (SDI: 0.85) had the highest prevalence rate at 9 368.64 per 100 000 (95% UI: 6 993.13 to 12 320.51), whereas Central Asia (SDI: 0.68) had the lowest prevalence rate at 3 038.39 per 100 000 (95% UI: 2 143.85 to 4 257.92). Central Latin America saw the greatest increase in prevalence rate (EAPC: 0.68; 95% CI: 0.39 to 0.98), while High-income Asia Pacific had the smallest increase (EAPC: 0.01; 95% CI: -0.15 to 0.17). East Asia experienced a decrease in prevalence (EAPC: -0.29; 95% CI: -0.45 to -0.13). In 2021, the global SDI was 0.67; 13 regions, including South Asia, had prevalence rates higher than the global average (4 976.61 per 100 000), while 8 regions had lower rates (**Supplementary Table 1** and **Figure 3B**).

#### 3.4.3 DALYs

In 2021, South Asia had the highest number of DALYs related to anxiety disorders among adolescents and young adults aged 10–24 years (2 100 000; 95% UI: 1 300 000 to 3 000 000), while Oceania had the fewest (25 000; 95% UI: 14 000 to 40 000). Western Europe (SDI: 0.85) had the highest DALY rate at 1 100 per 100 000 (95% UI: 720 to 1 700), whereas Central Asia (SDI: 0.68) had the lowest at 370 per 100 000 (95% UI: 230 to 570). From 1990 to 2021, Central Latin America had the greatest increase in DALY rate (EAPC: 0.69; 95% CI: 0.40 to 0.98), while Oceania had the smallest increase (EAPC: 0.02; 95% CI: -0.14 to 0.17). In 2021, the global SDI was 0.67; 11 regions, including Eastern Sub-Saharan Africa, had DALY rates higher than the global average (610 per 100 000), while 10 regions had lower rates (**Supplementary Table 1** and **Figure 3C**).

### 3.5 National trends

#### 3.5.1 Incidence

**Figure 4:**
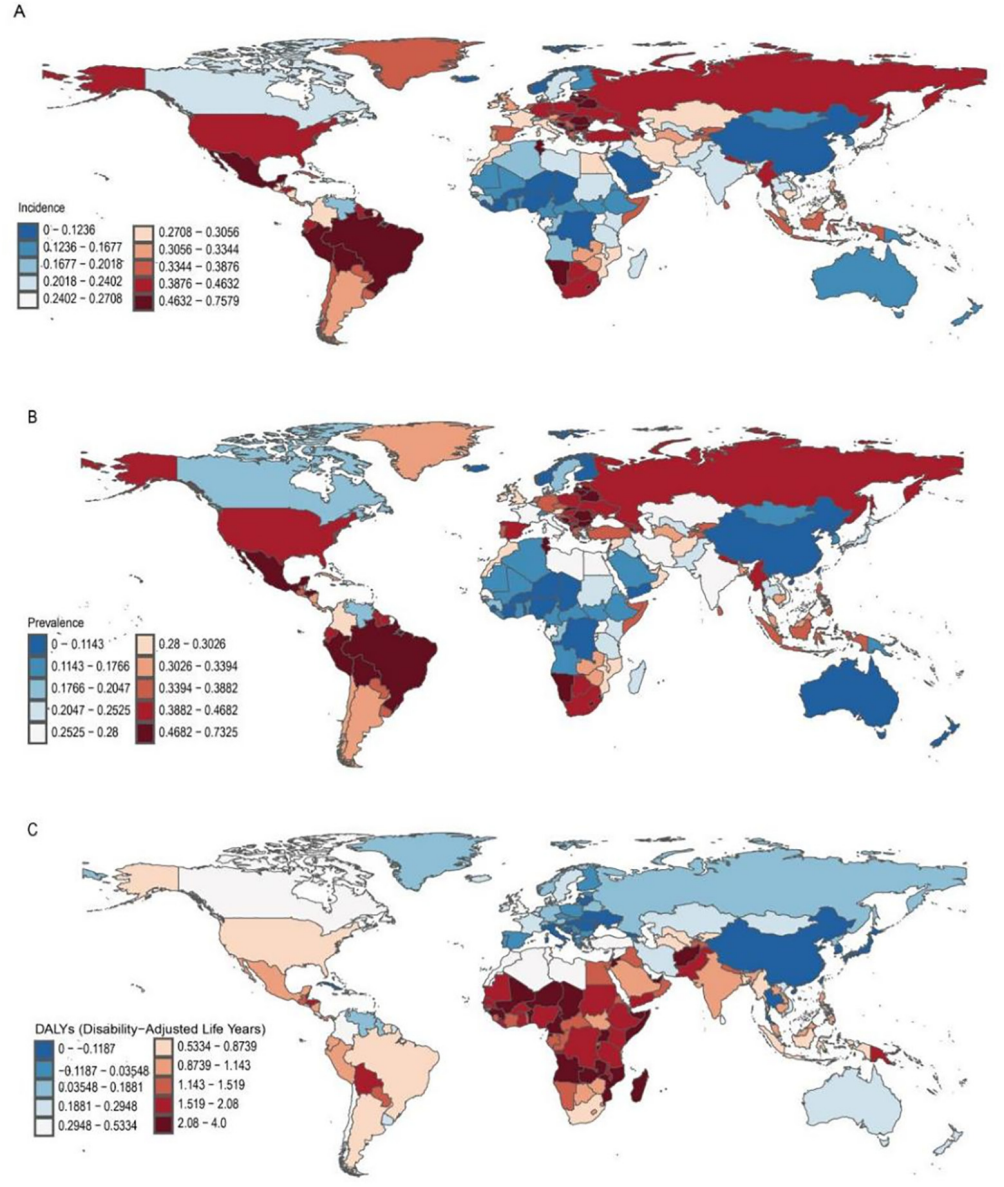
Change in Incident, Prevalence, and Disability-Adjusted Life-Years (DALYs) Cases of Anxiety Disorders in Adolescents and Young Adults Aged 10–24 Years in 204 Countries and Territories. **(A)** incident cases. **(B)** prevalence cases. **(C)** DALYs cases.

In 2021, India reported the highest number of anxiety disorder cases among adolescents and young adults aged 10–24 years (2 600 000; 95% UI: 1 800 000 to 3 500 000). Tokelau had the fewest cases. Portugal (SDI: 0.74) had the highest incidence rate at 1 900 per 100 000 (95% UI: 1 300 to 2 800), while Mongolia (SDI: 0.62) had the lowest incidence rate. Mexico showed the largest increase in incidence rate (EAPC: 1.40; 95% CI: 1.07 to 1.73), whereas China had the largest decrease (EAPC: -0.44; 95% CI: -0.68 to -0.21) (**Supplementary Table 2** and **Figure 4A**). The incidence rate in 127 countries was higher than the global average, while 77 countries had rates below the global average (**Supplementary Figure 3A**).

#### 3.5.2 Prevalence

In 2021, India had the highest prevalence of anxiety disorders among adolescents and young adults aged 10–24 years (12 665 987; 95% UI: 9 972 263 to 16 100 148) (**Supplementary Table 2** and **Figure 4B**). Portugal (SDI: 0.74) had the highest prevalence rate at 13,346.22 per 100,000 (95% UI: 8,726.43 to 18,944.25), while Mongolia (SDI: 0.62) had the lowest prevalence rate at 2 580.46 per 100,000 (95% UI: 1,623.39 to 3,772.35) (**Supplementary Table 2** and **Figure 1B**). Mexico experienced the largest increase in prevalence rate (EAPC: 1.28; 95% CI: 0.88 to 1.69). The Philippines, Taiwan (Province of China), and Singapore showed the greatest decreases in prevalence (EAPC: -0.47; 95% CI: -0.60 to -0.34) (**Supplementary Table 2** and **Figure 2B**). In 2021, the global prevalence rate of anxiety disorders among adolescents and young adults aged 10–24 years was 0.28% (95% UI: 0.23 to 0.33); the prevalence rate in 132 countries was higher than the global average, while 72 countries had rates below the global average (**Supplementary Figure 3B**).

#### 3.5.3 DALYs

In 2021, India had the highest number of DALYs related to anxiety disorders among adolescents and young adults aged 10–24 years (1 546 897; 95% UI: 988 390 to 2 202 786). Tokelau had the fewest DALYs (2.40; 95% UI: 1.33 to 3.92) (**Supplementary Table 2** and **Figure 4C**). Portugal (SDI: 0.74) had the highest DALY rate at 1 628.75 per 100 000 (95% UI: 901.99 to 2 580.12), while Mongolia (SDI: 0.62) had the lowest DALY rate at 317.90 per 100 000 (95% UI: 182.18 to 517.76). Mexico had the largest increase in DALYs (EAPC: 1.28; 95% CI: 0.88 to 1.69), while Taiwan (Province of China) had the largest decrease (EAPC: -0.46; 95% CI: -0.59 to -0.33) (**Supplementary Table 2** and **Figure 2C**). In 2021, the global DALY rate for anxiety disorders among adolescents and young adults aged 10–24 years was 610.44 per 100 000 (95% UI: 385.28 to 895.81); the DALY rate in 133 countries was higher than the global average, while 71 countries had rates below the global average (**Supplementary Figure 3C**).

## 4 Risk factors for anxiety disorders in adolescents and young adults aged 10–24 years

The GBD database identifies one primary risk factor for anxiety disorders in adolescents and young adults aged 10–24 years: bullying victimization. In 2021, bullying victimization was responsible for 1 703 468 disability-adjusted life years (DALYs) attributed to anxiety disorders globally (95% UI: 652,601 to 3,415,726). This accounted for 14.77% (95% UI: 6.66% to 26.46%) of the total DALYs associated with both childhood sexual abuse and bullying. This means that bullying alone contributed to a significant portion of the disease burden that results from both bullying and childhood sexual abuse combined. From 1990 to 2021, the DALY rate due to bullying victimization-induced anxiety disorders increased from 73.99 per 100 000 (95% UI: 27.09 to 151.52) to 90.24 per 100 000 (95% UI: 34.57 to 180.94), representing a relative increase of 22.00% (95% UI: 15.00% to 33.00%).

Among 21 geographic regions, South Asia had the highest proportion of DALYs of anxiety disorders attributable to bullying victimization at 18.91% (95% UI: 8.41% to 32.55%), while Central Asia had the lowest proportion of DALYs of anxiety disorders attributable to bullying victimization at 5.08% (95% UI: 1.91% to 10.63%). Notably, in nine regions, the DALY rate due to bullying victimization-related anxiety disorders in adolescents and young adults aged 10–24 years was below the global average (**Supplementary Figure 4**).

## 5 Joinpoint analysis

**Figure 5:**
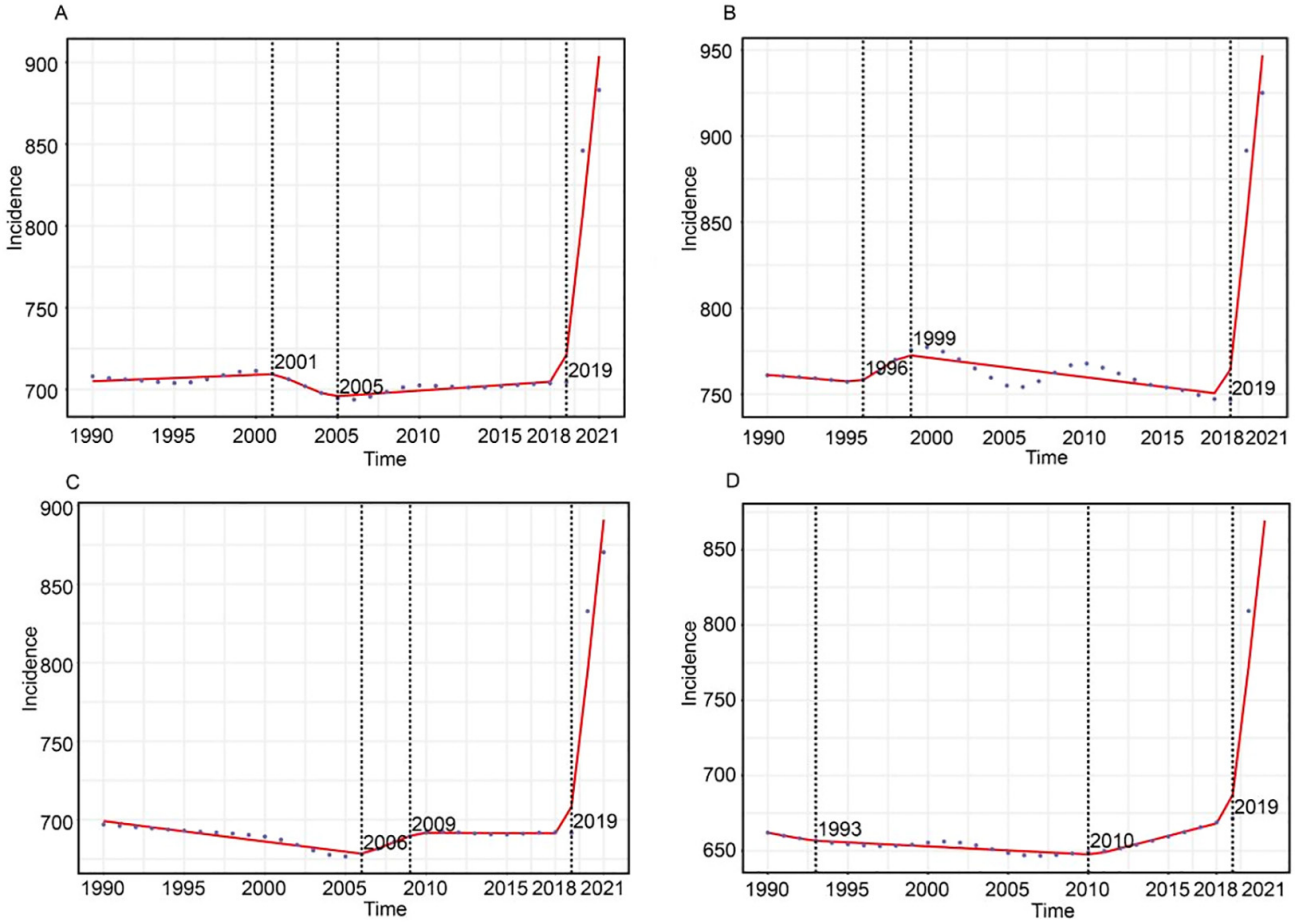
Joinpoint regression analysis of adolescents and young adults incidence for anxiety disorders globally from 1990 to 2021. **(A)** illustrates the shift in incidence trends for 10-24 age groups. **(B)** Illustrates the shift in incidence trends for 10-14 age groups. **(C)** Illustrates the shift in incidence trends for 15-19 age groups. **(D)** Illustrates the shift in incidence trends for 20-24 age groups.

The Joinpoint regression analysis on the incidence of anxiety disorders among adolescents and young adults from 1990 to 2021 revealed an overall upward trend in the 10-24 age group, with an average annual percent change (AAPC) of 6.25 (5.48, 7.02). From 1990 to 2001, there was a gradual decline, with an annual percent change (APC) of -2.26 (-2.32, -2.21). This decline continued from 2001 to 2005 with an APC of -0.08 (-0.19, 0.02). However, from 2005 to 2019, the incidence rate began to rise again, though at a slower pace, with an APC of 0.42 (0.06, 0.78) (P<0.05). Notably, from 2019 to 2021, the incidence rate increased significantly, with an APC of 12.49(10.67, 14.35) (P<0.05). Specifically, the incidence rates in the 10-14, 15-19, and 20-25 age groups also showed significant increases after 2019, with APCs of 11.30 (9.27, 13.38), 12.17 (10.31, 14.07), and 12.49 (10.67, 14.35), respectively, all with P-values less than 0.05, indicating statistical significance (**Figure 5**).

## 6 Discussion

Over the past 30 years, the incidence of anxiety disorders has increased significantly, particularly among adolescents and young adults. Females have consistently shown higher rates of anxiety across all age groups. Our analysis of global trends in anxiety disorders from 1990 to 2021, focusing on individuals aged 10 to 24, revealed a 52% rise in global incidence, with the sharpest increase occurring in the 20-24 age group. The higher prevalence among females is likely driven by a combination of biological, psychological, and social factors, highlighting the need for gender-specific mental health strategies. This persistent gender disparity, supported by various studies (14, 15), emphasizes the importance of targeted interventions to address the growing burden of anxiety disorders, especially in regions with differing socioeconomic conditions.

When examining the burden of anxiety disorders across different Socio-Demographic Index (SDI) regions, the study found that middle SDI regions had the highest number of cases, while high SDI regions showed the greatest increase in incidence. This higher concentration in high SDI regions is likely due to the greater availability of mental health services, leading to more accurate diagnosis and reporting. In contrast, low SDI regions may experience underreporting due to limited access to diagnostic services, mental health screening, and medical care. These findings align with previous studies by Polanczyk and Kessler et al. (22–24), which highlight significant disparities in mental disorders across various socioeconomic backgrounds. To address these differences, future intervention strategies must be tailored to the socioeconomic and cultural contexts of each region. In low SDI regions, enhancing mental health education and strengthening community support should be prioritized, while in high SDI regions, expanding mental health services, particularly for women and young people, is essential. By adopting a “global localization” strategy, which adapts global health policies to local needs, culturally relevant and effective interventions can be designed to address the growing burden of anxiety disorders worldwide.

Significant regional variations in anxiety disorder trends were observed across 21 global regions, with Tropical Latin America exhibiting the highest incidence rates and East Asia showing the smallest increase. These differences may be influenced by factors such as cultural norms, economic stressors, and healthcare infrastructure. Notably, bullying victimization emerged as a key risk factor for anxiety disorders, particularly in South Asia, where the highest DALY rates were recorded. This finding corroborates previous studies by Moore and Winsper et al. (25–27), highlighting the critical need for comprehensive anti-bullying policies and psychological support systems. To address the long-term mental health effects of bullying among adolescents and young adults, targeted intervention programs and expanded access to mental health services are essential. Schools should prioritize anti-bullying education and social-emotional learning, while also providing thorough training for teachers and parents to identify and respond to bullying. Additionally, establishing peer support systems, promoting mental health awareness, and creating a safe, inclusive school environment are crucial steps to reduce the psychological stress faced by affected students.

Our analysis also revealed stark differences at the country level, with India having the highest number of anxiety disorder cases and Portugal reporting the highest incidence and DALY rates, while Mongolia had showed the lowest incidence and prevalence rates. Mexico experienced the largest increase in incidence, while China saw the greatest decrease. Similarly, India had the highest number of prevalent cases, Portugal had the highest prevalence rate, and Mongolia had the lowest, with Mexico again showing the largest increase, and the Philippines, Singapore, and Taiwan seeing the greatest decreases. In terms of DALYs, India had the highest number, Tokelau had the fewest, with Portugal showing the highest DALY rate and Mongolia the lowest. Mexico experienced the largest increase in DALYs, while Taiwan saw the greatest decrease. These findings highlight the need for country-specific and regional interventions to improve the mental health of young people. Differences in cultural and social contexts across these countries may significantly influence the prevalence and incidence of anxiety disorders. For example, social expectations, mental health stigmatization, family structures, and coping mechanisms can all shape anxiety disorders. in collectivist cultures, such as those in many Asian countries, stronger social normative pressure, may increase social anxiety, while in individualistic cultures, like in Western regions, the focus on personal achievement and competition can also trigger anxiety. Additionally, in some regions, mental health stigmatization may prevent individuals from seeking help, leading to underreporting of anxiety disorders.

The Joinpoint analysis results indicate an overall upward trend in the incidence of anxiety disorders among the 10-24 age group. After a gradual decline from 1990 to 2005, this trend reversed, with a particularly sharp increase after 2019. This sudden rise is likely linked to the global COVID-19 pandemic, which introduced known risk factors for anxiety disorders, such as social isolation, economic uncertainty, and heightened psychological stress (28). During the pandemic, young people faced challenges, including adapting to online learning, social isolation, reduced household income, and future uncertainties, all of which contributed to the increased incidence of anxiety disorders (29, 30). In response, various global interventions were implemented, such as enhancing mental health support hotlines, providing online psychological counseling, conducting community support activities, promoting physical activity and encouraging self-management techniques like mindfulness and meditation (31, 32). These interventions aimed to mitigate the psychological burden of the pandemic and offer necessary support to adolescents and young adults. Consistent with findings by Solmi M,et al which also reported a significant increase in anxiety disorders among youth during the pandemic (33, 34), These results align with the broader global trend of rising mental health challenges among young people, driven modern life pressures and increased use of digital media use. This study underscores the evolving epidemiology of anxiety disorders in young populations, and highlights the urgent need for targeted mental health interventions and policy measures to address this growing public health concern.

However, this study has several limitations. The reliance on the GBD database may introduce biases due to regional variations in data collection and reporting practices. While cross-sectional data inherently limit the exploration of temporal relationships, they still offer a valuable snapshot of current trends. Although bullying victimization is identified as a significant risk factor, other potential influences, such as family abuse, socioeconomic stress, and genetic predispositions, were not fully accounted for, which may affect the outcomes. Additionally, the aggregation of various anxiety disorders introduces heterogeneity, as these disorders differ in onset age, risk factors, severity, functional impairment, and comorbidity patterns, For instance, specific phobias typically manifest in early childhood, while social anxiety and panic disorders often emerge during adolescence, each with distinct characteristics. Moreover, GBD diagnostic estimates, derived from diverse sources, such as administrative records, health data, and surveys, may not represent nationally samples, potentially leading to estimates that reflect only cases seen in clinical care. Future research should further explore investigate other factors influencing the burden of anxiety disorders, including family abuse, economic stress, and genetic factors, which were not fully considered in this study. Additionally, since different types of anxiety disorders vary in their course, risk factors, and comorbidity patterns, future studies should conduct more detailed stratified analyses on disorders such as generalized anxiety disorder, social anxiety disorder, panic disorder to develop more targeted interventions for different at-risk populations. Longitudinal studies exploring the long-term health impacts of anxiety disorders and their association with socioeconomic changes and public policies would provide more robust evidence for shaping sustainable public health strategies.

## 7 Conclusion

In light of these findings, the increasing trend of anxiety disorder incidence among adolescents and young adults over the past 30 years is closely linked to the rising global burden, particularly in high SDI regions where the prevalence and incidence have surged the most. Middle SDI regions, reporting the highest number of anxiety disorder cases and disability-adjusted life years (DALY) counts, highlight the significant impact of socio-economic factors on mental health. This study also underscores the critical role of bullying victimization as a major risk factor, especially in South Asia, contributing to the regional disparities in anxiety disorders. The pronounced increase in these disorders, exacerbated by the COVID-19 pandemic, emphasizes the urgent need for policymakers to develop cost-effective and targeted strategies. Such strategies should focus on reducing the incidence of anxiety disorders through public awareness campaigns, effective interventions, and strengthened social support systems. Prioritizing early prevention and community-based mental health management is essential to address this escalating mental health challenge and mitigate its long-term impact on young populations worldwide.

## Data Availability

Publicly available datasets were analyzed in this study. This data can be found here: http://ghdx.healthdata.org/gbd-2021, the Global Health Data Exchange query tool created by GBD collaborators.
