# Supplementary material for: Rising global burden of anxiety disorders among adolescents and young adults: trends, risk factors, and the impact of socioeconomic disparities and COVID-19 from 1990 to 2021
Source: Front Psychiatry. 2024 Nov 26;15:1489427. doi: 10.3389/fpsyt.2024.1489427 (PMC11651023; doi:10.3389/fpsyt.2024.1489427)
Supplement: Supplementary file 1 [file SupplementaryFile1.docx]

***Supplementary Material***

**Supplementary Figures and Tables are as follows:**

**Supplementary Figure 1.** The Incidence, Prevalence, and DALYs Rates of Anxiety Disorders in Adolescents and Young Adults Aged 10–24 Years in 204 Countries and Territories

**Supplementary Figure 2.** The National Burden of Anxiety Disorders in Adolescents and Young Adults Aged 10–24 Years in 204 Countries and Territories

**Supplementary Figure 3.** Incidence, Prevalence, and DALYs Rates of Anxiety Disorders in Adolescents and Young Adults Aged 10–24 Years in 204 Countries by SDI in 2021

**Supplementary Figure 4.** Proportion of DALYs of anxiety disorders attributable to bullying victimization in adolescents and young adults aged 10–24 years

**Supplementary Table 1.** Prevalence and DALYs of Anxiety Disorders in Adolescents and Young Adults Aged 10–24 Years Between 1990 and 2021 at the Global and Regional Levels

**Supplementary Table 2.** Incidence, Prevalence, and DALYs of Anxiety Disorders in Adolescents and Young Adults Aged 10–24 Years at the National Level


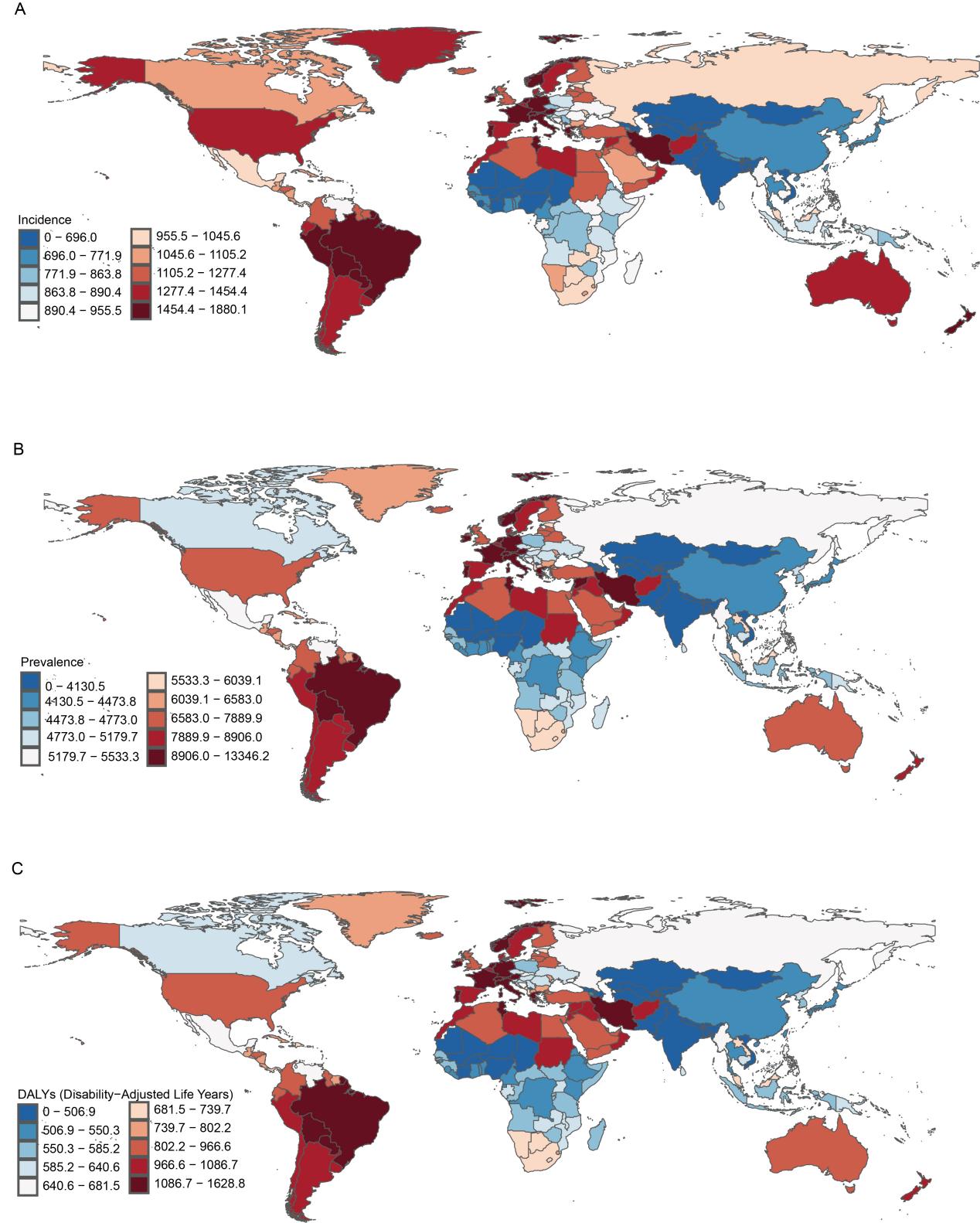


**Supplementary Figure 1.** The incidence, prevalence, and DALYs rates of anxiety disorders in adolescents and young adults aged 10–24 years in 204 countries and territories. (A) Disease burden of incidence rate. (B) Disease burden of prevalence rate. (C) Disease burden of DALYs rate. DALYs=disability-adjusted life-years.

**
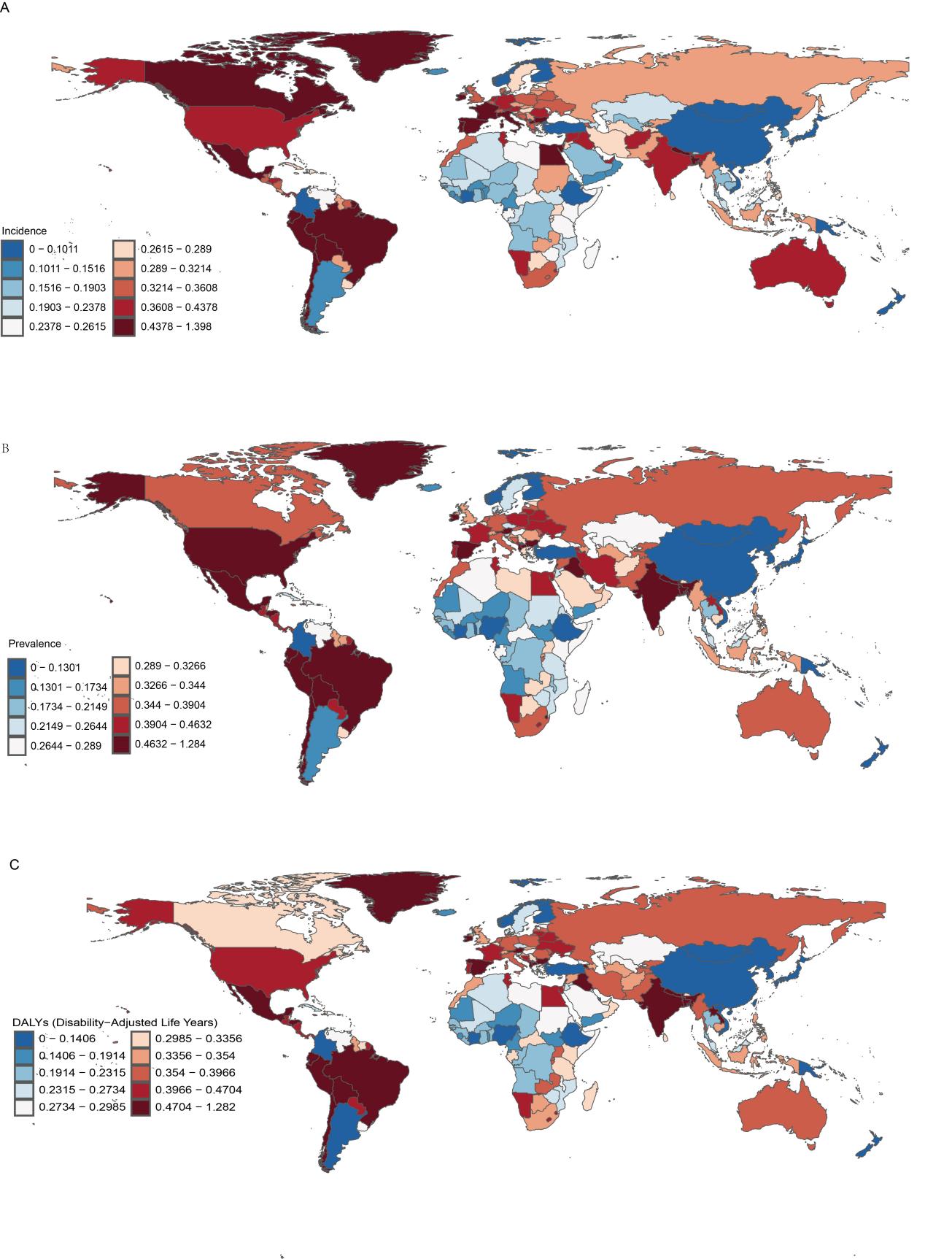
**

**Supplementary Figure 2.** The national burden of anxiety disorders in adolescents and young adults aged 10–24 years in 204 countries and territories. (A) EAPC for incidence rate. (B) EAPC for prevalence rate. (C) EAPC for DALYs rate. DALYs=disability-adjusted life-years; EAPC=estimated annual percentage change.

**
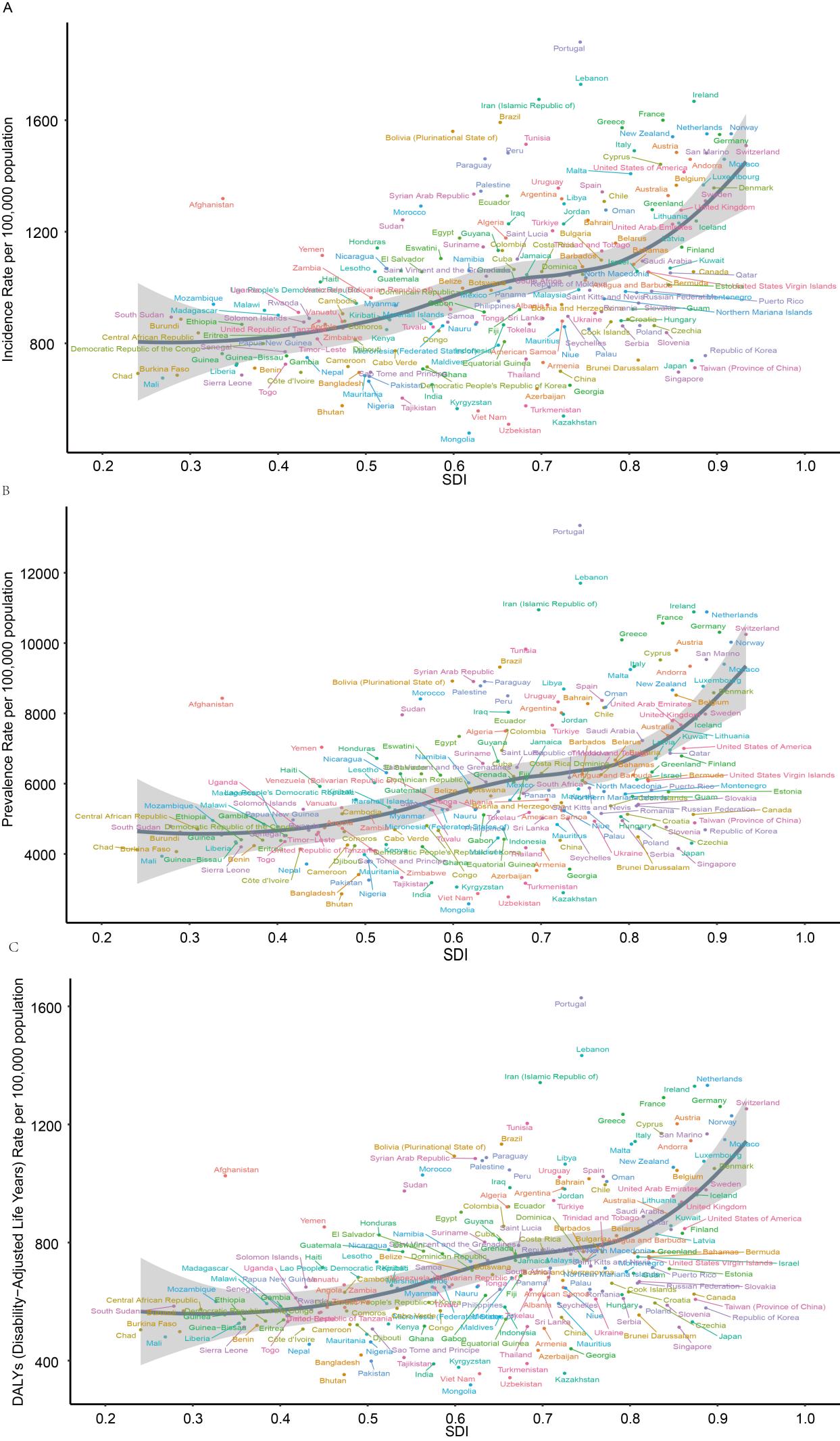
**

**Supplementary Figure 3.** Incidence, Prevalence, and DALYs rates of anxiety disorders in adolescents and young adults aged 10–24 years in 204 countries by SDI in 2021. (A) Incidence rate. (B) prevalence rate. (C) DALYs rate. DALYs=disability-adjusted life-years; SDI= socio-demographic index.


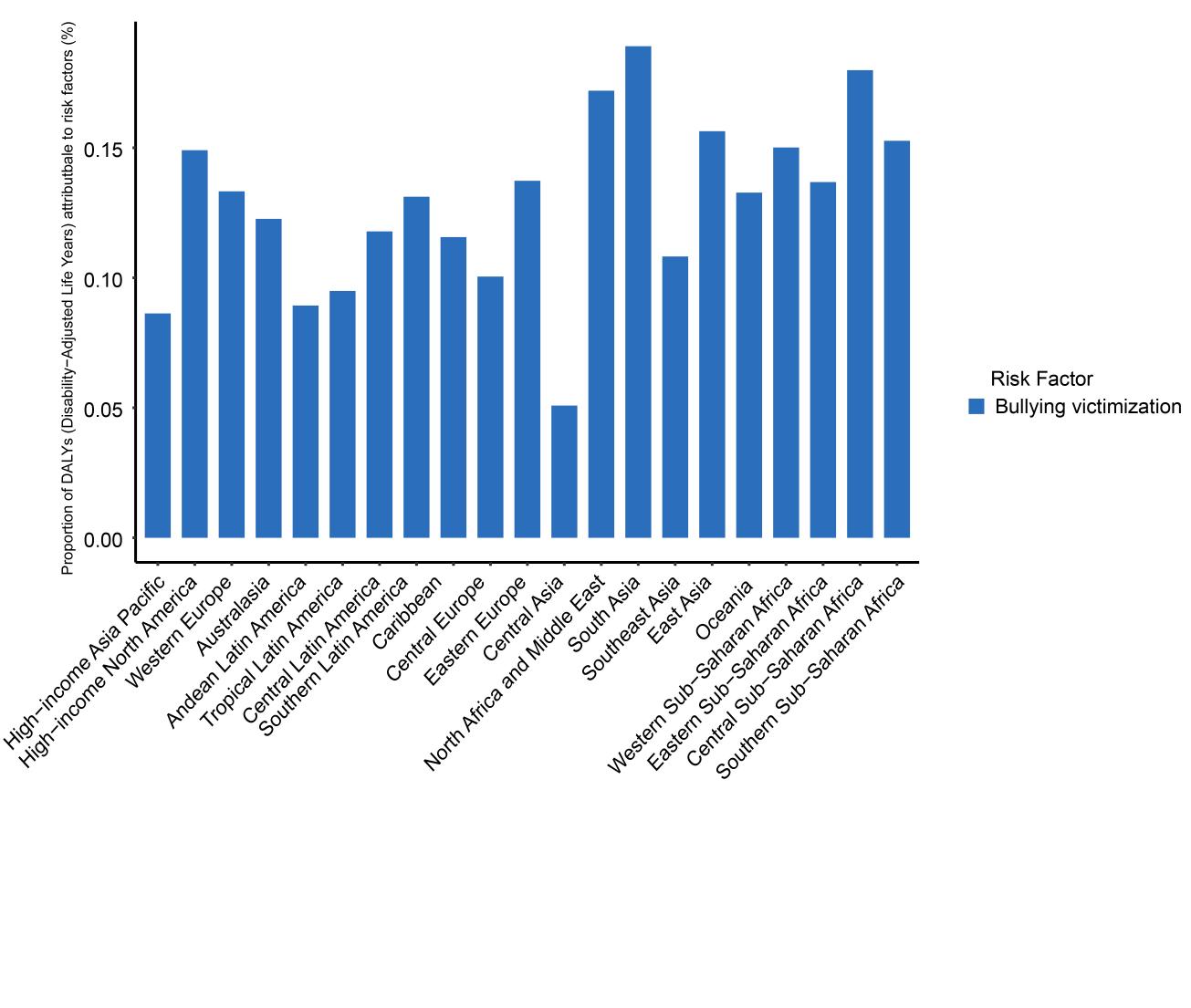


**Supplementary Figure 4.** Proportion of DALYs of anxiety disorders attributable to bullying victimization in adolescents and young adults aged 10–24 years in 21 regions.

| **Supplementary Table 1**. Prevalence and DALYs of anxiety disorders in adolescents and young adults aged 10–24 Years between 1990 and 2021 at the global and regional levels | | | | | | |
| --- | --- | --- | --- | --- | --- | --- |
| location | Rate per 100 000 (95% UI) | | | | |  |
|  | 1990 | | 2021 | | 1990-2021 |  |
|  | Prevalence cases | Prevalence rate | Prevalence cases | Prevalence rate | Cases change | EAPCa |
| Global | 63753648.210(49650307.708-80523297.472) | 4120.598(3209.055-5204.473) | 93947156.541(71898221.622-120279390.626) | 4976.610(3808.624-6371.493) | 0.474(0.437-0.511) | 0.155(-0.010-0.320) |
| **Socio-demographic index** |  |  |  |  |  |  |
| High SDI | 10265438.400(7985506.137-12992205.173) | 5241.282(4077.204-6633.502) | 12979927.145(10096444.623-16637656.373) | 6994.444(5440.633-8965.471) | 0.264(0.208-0.324) | 0.445(0.251-0.640) |
| High-middle SDI | 12758064.115(10061060.929-15985773.890) | 4495.689(3545.318-5633.069) | 12626310.204(9562158.145-16260637.825) | 5589.828(4233.289-7198.791) | -0.01(-0.075-0.053) | 0.178(0.009-0.347) |
| Middle SDI | 23184572.834(18346350.152-29012881.761) | 4224.389(3342.832-5286.347) | 29018755.166(22578283.733-36378105.273) | 5249.935(4084.756-6581.354) | 0.252(0.21-0.299) | 0.177(0.004-0.349) |
| Low-middle SDI | 12050197.164(9201960.793-15433349.432) | 3331.426(2543.996-4266.740) | 23638317.214(17899735.990-30770373.486) | 4276.530(3238.334-5566.827) | 0.962(0.864-1.058) | 0.427(0.227-0.628) |
| Low SDI | 5438252.920(4040773.083-7147230.869) | 3493.662(2595.888-4591.550) | 15602914.974(11559359.807-20929763.121) | 4223.665(3129.086-5665.627) | 1.869(1.725-2.022) | 0.316(0.173-0.459) |
| **Regions** |  |  |  |  |  |  |
| Andean Latin America | 668245.600(480094.440-907537.321) | 5428.315(3899.919-7372.137) | 1433007.345(977546.017-2009693.375) | 8300.424(5662.250-11640.768) | 1.144(0.824-1.51) | 0.564(0.188-0.941) |
| Australasia | 342549.378(256530.569-455729.356) | 7119.947(5332.032-9472.413) | 435974.194(308081.662-605828.062) | 7599.119(5369.926-10559.707) | 0.273(0.023-0.549) | 0.237(0.115-0.359 |
| Caribbean | 498980.763(356720.315-684607.390) | 4672.642(3340.462-6410.919) | 677109.279(473258.784-939649.462) | 5977.567(4177.961-8295.289) | 0.357(0.209-0.519) | 0.283(0.092-0.475) |
| Central Asia | 467987.798(332975.155-649928.690) | 2359.851(1679.043-3277.297) | 672317.161(474377.729-942167.763) | 3038.392(2143.848-4257.924) | 0.437(0.277-0.609) | 0.276(0.081-0.472) |
| Central Europe | 1024142.825(748657.160-1369986.526) | 3507.540(2564.042-4692.004) | 918275.530(668881.911-1209180.440) | 5063.211(3688.098-6667.210) | -0.103(-0.17--0.036) | 0.360(0.099-0.621) |
| Central Latin America | 2165885.132(1622084.870-2805278.105) | 3992.009(2989.714-5170.494) | 3868095.567(2899685.916-4995967.472) | 5947.887(4458.784-7682.191) | 0.786(0.649-0.946) | 0.683(0.389-0.977) |
| Central Sub-Saharan Africa | 691180.384(491466.037-949601.844) | 3993.447(2839.554-5486.534) | 2026256.202(1380660.583-2927159.158) | 4508.407(3071.961-6512.911) | 1.932(1.45-2.497) | 0.180(0.055-0.307) |
| East Asia | 16104914.899(12862764.989-20058140.373) | 4327.193(3456.067-5389.376) | 10701467.055(8347529.395-13402544.751) | 4403.703(3435.047-5515.209) | -0.336(-0.376--0.294) | -0.289(-0.452--0.126) |
| Eastern Europe | 1765309.845(1400063.577-2212931.885) | 3737.797(2964.439-4685.575) | 1778600.656(1422142.312-2232178.505) | 5390.548(4310.201-6765.243) | 0.008(-0.046-0.073) | 0.367(0.114-0.620) |
| Eastern Sub-Saharan Africa | 2326197.777(1756665.974-3024134.861) | 3749.906(2831.802-4875.003) | 6703578.784(4971447.756-8873405.407) | 4609.287(3418.298-6101.230) | 1.882(1.699-2.086) | 0.229(0.065-0.392) |
| High-income Asia Pacific | 1547670.622(1207768.119-1974842.812) | 3673.722(2866.892-4687.705) | 1152500.923(875702.183-1487505.988) | 4414.875(3354.544-5698.176) | -0.255(-0.321--0.187) | 0.010(-0.147-0.168) |
| High-income North America | 2928193.004(2334309.623-3577948.909) | 4786.724(3815.902-5848.882) | 4869458.646(3884058.569-6019210.364) | 6831.523(5449.073-8444.548) | 0.663(0.571-0.762) | 0.477(0.199-0.757) |
| North Africa and Middle East | 7175823.264(5462693.566-9382922.362) | 6589.001(5015.967-8615.609) | 13249367.659(9682135.219-18158898.297) | 8163.651(5965.687-11188.678) | 0.846(0.695-0.98) | 0.283(0.116-0.450) |
| Oceania | 92109.166(64842.309-127686.097) | 4403.106(3099.665-6103.794) | 203886.801(136455.106-297031.735) | 5056.243(3383.986-7366.168) | 1.214(0.767-1.715) | 0.125(0.036-0.214) |
| South Asia | 8508500.444(6579949.210-10756241.085) | 2543.878(1967.279-3215.910) | 16949401.086(13184204.583-21187937.888) | 3223.110(2507.117-4029.113) | 0.992(0.882-1.113) | 0.603(0.259-0.947) |
| Southeast Asia | 5038174.158(3850518.642-6510794.014) | 3395.769(2595.280-4388.327) | 7986328.734(6166544.225-10235818.652) | 4670.115(3605.971-5985.535) | 0.585(0.504-0.671) | 0.370(0.166-0.574) |
| Southern Latin America | 805810.395(605529.555-1063025.767) | 6087.679(4574.612-8030.872) | 1237190.177(821763.694-1728447.805) | 8066.982(5358.233-11270.182) | 0.535(0.253-0.865) | 0.246(0.002-0.490) |
| Southern Sub-Saharan Africa | 672514.702(518865.625-852280.175) | 3936.599(3037.207-4988.866) | 1203025.501(932658.686-1501952.281) | 5514.792(4275.403-6885.103) | 0.789(0.655-0.955) | 0.327(0.088-0.567) |
| Tropical Latin America | 2770852.063(2256456.912-3395112.396) | 5789.549(4714.747-7093.908) | 4703678.671(3742320.267-5875616.942) | 9300.106(7399.310-11617.261) | 0.698(0.559-0.834) | 0.662(0.360-0.964) |
| Western Europe | 5985515.825(4535361.262-7759890.195) | 7281.603(5517.436-9440.196) | 6752487.307(5040327.207-8880059.595) | 9368.643(6993.131-12320.514) | 0.128(0.046-0.221) | 0.392(0.186-0.599) |
| Western Sub-Saharan Africa | 2173090.167(1644459.565-2850427.189) | 3631.274(2747.922-4763.116) | 6425149.262(4780662.186-8440338.215) | 3981.735(2962.629-5230.570) | 1.957(1.817-2.109) | 0.124(0.025-0.222) |
| location | Rate per 100 000 (95% UI) | | | | |  |
|  | 1990 | | 2021 | | 1990-2021 |  |
|  | DALYs cases | DALYs rate | DALYs cases | DALYs rate | Cases change | EAPCa |
| Global | 7822521.631(4925458.665-11445028.621) | 505.594(318.348-739.728) | 11523716.425(7273257.711-16910889.818) | 610.439(385.282-895.811) | 0.473(0.434-0.511) | 0.158(-0.006-0.321) |
| **Socio-demographic index** |  |  |  |  |  |  |
| High SDI | 1258743.657(786738.146-1850573.793) | 642.684(401.689-944.858) | 1584215.402(1000565.945-2303099.688) | 853.680(539.171-1241.063) | 0.259(0.204-0.319) | 0.435(0.242-0.627) |
| High-middle SDI | 1574262.080(1013840.966-2314136.220) | 554.739(357.257-815.456) | 1558091.642(976352.972-2307594.591) | 689.787(432.244-1021.601) | -0.01(-0.077-0.053) | 0.182(0.014-0.350) |
| Middle SDI | 2854434.876(1829943.378-4168482.037) | 520.098(333.428-759.526) | 3572196.725(2263863.397-5228392.031) | 646.265(409.567-945.896) | 0.251(0.206-0.301) | 0.179(0.008-0.351) |
| Low-middle SDI | 1468967.963(922131.397-2155675.542) | 406.114(254.935-595.963) | 2893763.527(1835906.311-4230363.598) | 523.526(332.143-765.337) | 0.97(0.866-1.075) | 0.445(0.247-0.644) |
| Low SDI | 659107.121(411569.985-970693.380) | 423.426(264.402-623.596) | 1905521.994(1156774.397-2829339.522) | 515.819(313.136-765.894) | 1.891(1.739-2.052) | 0.349(0.207-0.491) |
| **Regions** |  |  |  |  |  |  |
| Andean Latin America | 81951.706(49863.270-123218.000) | 665.713(405.051-1000.929) | 176030.454(103403.383-270836.454) | 1019.623(598.945-1568.769) | 1.148(0.812-1.505) | 0.575(0.203-0.948) |
| Australasia | 41623.583(25840.405-61459.226) | 865.153(537.097-1277.441) | 53006.999(31679.231-78764.325) | 923.923(552.175-1372.878) | 0.273(0.023-0.55) | 0.243(0.124-0.363) |
| Caribbean | 60981.643(36466.394-92749.706) | 571.055(341.485-868.543) | 82588.505(48851.949-129329.175) | 729.097(431.268-1141.727) | 0.354(0.198-0.519) | 0.285(0.096-0.474) |
| Central Asia | 57509.112(34304.083-87761.754) | 289.992(172.980-442.543) | 82852.130(49892.090-125285.026) | 374.432(225.477-566.199) | 0.441(0.273-0.623) | 0.285(0.090-0.481) |
| Central Europe | 126202.114(77354.138-188998.806) | 432.224(264.927-647.293) | 113205.316(69639.740-168854.061) | 624.194(383.981-931.032) | -0.103(-0.166--0.029) | 0.365(0.105-0.625) |
| Central Latin America | 266159.969(166188.753-397367.959) | 490.568(306.308-732.401) | 475926.605(295684.230-704186.287) | 731.822(454.667-1082.812) | 0.788(0.642-0.958) | 0.688(0.395-0.982) |
| Central Sub-Saharan Africa | 83394.952(50991.596-125853.016) | 481.833(294.615-727.144) | 246730.969(147587.140-385307.457) | 548.975(328.380-857.307) | 1.959(1.455-2.587) | 0.220(0.095-0.346) |
| East Asia | 1996747.388(1297881.229-2902880.995) | 536.501(348.725-779.968) | 1333187.891(858073.038-1973010.501) | 548.613(353.101-811.903) | -0.332(-0.374--0.292) | -0.273(-0.438--0.107) |
| Eastern Europe | 217432.140(140543.409-320152.910) | 460.382(297.581-677.879) | 219466.563(140098.040-322755.749) | 665.155(424.606-978.202) | 0.009(-0.046-0.078) | 0.378(0.126-0.630) |
| Eastern Sub-Saharan Africa | 282463.083(176003.671-413195.826) | 455.340(283.724-666.085) | 820303.157(500696.842-1217615.800) | 564.029(344.272-837.216) | 1.904(1.702-2.109) | 0.266(0.104-0.427) |
| High-income Asia Pacific | 190850.462(120185.188-283940.549) | 453.024(285.285-673.993) | 142181.153(89112.537-208752.902) | 544.652(341.363-799.668) | -0.255(-0.324--0.181) | 0.015(-0.142-0.171) |
| High-income North America | 357810.630(234352.738-510226.748) | 584.914(383.097-834.069) | 589456.934(384993.485-854034.925) | 826.968(540.120-1198.154) | 0.647(0.553-0.745) | 0.456(0.180-0.732) |
| North Africa and Middle East | 878966.871(550237.859-1276171.591) | 807.087(505.241-1171.809) | 1623594.195(1002403.573-2401134.487) | 1000.384(617.635-1479.469) | 0.847(0.694-0.987) | 0.286(0.120-0.452) |
| Oceania | 11284.835(6813.659-17269.275) | 539.450(325.714-825.525) | 25121.428(13988.463-40143.365) | 622.993(346.904-995.526) | 1.226(0.794-1.76) | 0.143(0.055-0.232) |
| South Asia | 1034131.989(658731.100-1505541.140) | 309.186(196.948-450.128) | 2071745.669(1315567.167-2971456.544) | 393.965(250.169-565.054) | 1.003(0.894-1.128) | 0.626(0.285-0.968) |
| Southeast Asia | 619650.356(388123.720-916651.468) | 417.649(261.598-617.830) | 985254.954(637138.279-1469551.264) | 576.141(372.575-859.340) | 0.59(0.51-0.684) | 0.385(0.183-0.587) |
| Southern Latin America | 98890.539(61991.562-145739.957) | 747.091(468.329-1101.026) | 151568.969(87572.268-231566.158) | 988.291(571.007-1509.905) | 0.533(0.232-0.858) | 0.245(0.004-0.487) |
| Southern Sub-Saharan Africa | 82372.107(52311.338-120809.916) | 482.169(306.207-707.167) | 147176.803(93425.875-212909.682) | 674.673(428.274-976.000) | 0.787(0.639-0.971) | 0.334(0.096-0.572) |
| Tropical Latin America | 337618.286(219720.573-480175.770) | 705.436(459.095-1003.302) | 572225.260(371418.611-824729.741) | 1131.403(734.368-1630.654) | 0.695(0.553-0.83) | 0.662(0.365-0.959) |
| Western Europe | 732724.461(450133.812-1075628.406) | 891.387(547.605-1308.542) | 825862.480(516691.127-1215370.077) | 1145.831(716.876-1686.248) | 0.127(0.05-0.22) | 0.393(0.188-0.598) |
| Western Sub-Saharan Africa | 263755.402(163790.596-392059.731) | 440.740(273.697-655.139) | 786229.990(487065.471-1172646.365) | 487.235(301.840-726.702) | 1.981(1.828-2.131) | 0.155(0.058-0.252) |

a EAPC is expressed as 95% CIs.

| **Supplementary Table 2. Incidence, Prevalence and DALYs of anxiety disorders in adolescents and young adults aged 10–24 Years between 1990 and 2021 at the national level** | | | | | | |
| --- | --- | --- | --- | --- | --- | --- |
| location | Rate per 100 000 (95% UI) | | | | | |
|  | 1990 | | 2021 | | 1990-2021 | |
|  | Incidence cases | Incidence rate | Incidence cases | Incidence rate | Cases change | EAPCa |
| Afghanistan | 34181.841(24617.711-46785.147) | 1012.133(728.936-1385.319) | 25056.252(16272.288-37281.064) | 1025.980(600.287-1613.265) | 3.11(2.194 - 4.133) | 0.433(0.239-0.628) |
| Albania | 6400.174(4417.666-9110.711) | 653.439(451.031-930.177) | 3939.845(2458.670-5956.090) | 682.370(380.359-1098.345) | -0.226(-0.4 - -0.017) | 0.330(0.043-0.618) |
| Algeria | 84570.835(61600.266-115190.727) | 993.430(723.601-1353.114) | 46558.611(29190.203-67633.084) | 920.606(528.673-1441.711) | 0.415(0.085 - 0.82) | 0.225(0.045-0.406) |
| American Samoa | 109.299(74.955-151.216) | 726.787(498.410-1005.513) | 144.194(89.638-214.539) | 647.483(371.623-1064.600) | 0.121(-0.128 - 0.408) | 0.232(0.125-0.340) |
| Andorra | 135.339(96.433-188.709) | 1114.082(793.815-1553.415) | 3862.292(2432.830-5671.565) | 1144.853(634.107-1807.767) | 0.405(0.097 - 0.778) | 0.478(0.253-0.704) |
| Angola | 23415.893(16050.443-33319.971) | 730.755(500.897-1039.838) | 7681.380(4843.597-11568.967) | 580.555(326.029-912.284) | 2.915(2.008 - 4.017) | 0.183(0.039-0.326) |
| Antigua and Barbuda | 139.255(94.669-200.347) | 801.294(544.738-1152.826) | 41952.596(25981.981-62051.919) | 739.712(406.840-1175.684) | 0.451(0.11 - 0.815) | 0.222(0.036-0.409) |
| Argentina | 87765.110(61754.957-121204.970) | 1007.380(708.832-1391.207) | 19685.909(12658.673-29109.568) | 983.401(541.607-1544.326) | 0.606(0.21 - 1.061) | 0.105(-0.155-0.365) |
| Armenia | 4291.346(2964.612-6011.604) | 492.777(340.428-690.315) | 25160.733(15957.759-37872.128) | 509.002(279.640-821.139) | -0.082(-0.309 - 0.186) | 0.332(0.010-0.656) |
| Australia | 46416.790(30858.626-66179.153) | 1169.154(777.272-1666.932) | 5082.797(3213.068-7828.324) | 895.877(516.245-1365.041) | 0.353(0.043 - 0.778) | 0.388(0.204-0.572) |
| Austria | 18384.570(12880.273-25187.492) | 1130.362(791.934-1548.635) | 312.549(198.129-458.229) | 1202.109(674.435-1918.878) | 0.141(-0.11 - 0.437) | 0.391(0.211-0.572) |
| Azerbaijan | 9547.228(6543.856-13518.978) | 451.409(309.404-639.200) | 4046.363(2565.571-6429.839) | 434.845(252.317-701.120) | 0.473(0.121 - 0.917) | 0.237(-0.037-0.512) |
| Bahamas | 643.106(444.682-917.003) | 795.791(550.257-1134.714) | 791.773(499.708-1215.034) | 765.380(437.196-1169.671) | 0.67(0.282 - 1.185) | 0.373(0.135-0.611) |
| Bahrain | 1314.466(937.608-1818.866) | 1016.884(725.343-1407.093) | 1845.990(1168.596-2868.201) | 1016.013(568.064-1628.584) | 2.171(1.427 - 3.099) | 0.131(-0.060-0.323) |
| Bangladesh | 189633.773(125773.978-275419.353) | 543.360(360.381-789.162) | 16778.487(10716.138-26132.299) | 419.054(233.868-662.752) | 0.68(0.274 - 1.197) | 0.468(0.259-0.677) |
| Barbados | 535.283(363.441-767.690) | 792.489(538.077-1136.569) | 10039.407(6317.112-15162.807) | 768.570(443.369-1250.278) | 0.147(-0.087 - 0.457) | 0.320(0.117-0.524) |
| Belarus | 15481.448(10899.374-21686.575) | 699.222(492.272-979.477) | 309134.721(219639.140-407091.342) | 823.396(462.564-1320.285) | 0.065(-0.182 - 0.39) | 0.360(-0.035-0.756) |
| Belgium | 21822.781(15505.062-30145.610) | 1084.051(770.217-1497.490) | 39019.041(25210.204-57837.523) | 1044.265(601.830-1643.543) | 0.23(-0.04 - 0.574) | 0.328(0.104-0.553) |
| Belize | 492.923(341.577-693.210) | 788.752(546.575-1109.242) | 823.404(537.122-1247.100) | 706.346(402.649-1122.593) | 1.673(1.033 - 2.444) | 0.293(0.093-0.494) |
| Benin | 9121.640(6502.117-12514.606) | 632.195(450.644-867.352) | 5865.353(3712.908-8947.007) | 519.325(287.449-842.034) | 2.414(1.566 - 3.463) | 0.124(0.031-0.217) |
| Bermuda | 99.051(66.741-144.222) | 795.132(535.765-1157.743) | 598956.617(419389.814-805534.667) | 752.257(421.824-1189.891) | -0.023(-0.262 - 0.283) | 0.317(0.119-0.516) |
| Bhutan | 1247.721(812.225-1802.634) | 548.374(356.973-792.258) | 87402.570(52475.041-134288.178) | 352.697(199.412-569.123) | -0.055(-0.286 - 0.244) | 0.190(0.131-0.249) |
| Bolivia (Plurinational State of) | 19461.638(13378.709-27395.858) | 971.290(667.703-1367.270) | 4953.676(3101.438-7399.134) | 1092.744(649.697-1734.579) | 1.593(1.003 - 2.351) | 0.579(0.159-1.001) |
| Bosnia and Herzegovina | 7333.941(5070.094-10368.770) | 637.845(440.955-901.789) | 14062.370(8791.949-22358.572) | 671.596(372.928-1063.427) | -0.307(-0.477 - -0.12) | 0.324(0.033-0.615) |
| Botswana | 3115.207(2159.605-4341.860) | 690.665(478.801-962.624) | 270.437(174.039-399.942) | 691.296(394.047-1132.538) | 1.078(0.61 - 1.655) | 0.262(0.020-0.504) |
| Brazil | 466600.020(334937.135-611106.133) | 1000.731(718.350-1310.657) | 40837.573(25124.206-62671.024) | 1133.193(738.585-1639.022) | 0.658(0.512 - 0.8) | 0.856(0.599-1.113) |
| Brunei Darussalam | 481.620(337.007-665.096) | 632.175(442.356-873.007) | 22121.748(14090.826-32864.082) | 554.412(312.518-875.588) | 0.629(0.237 - 1.084) | 0.101(-0.022-0.224) |
| Bulgaria | 11903.354(8205.363-16651.561) | 645.894(445.236-903.539) | 548.214(366.710-802.612) | 768.194(438.756-1242.666) | -0.104(-0.321 - 0.157) | 0.512(0.095-0.931) |
| Burkina Faso | 18398.086(13273.916-25315.500) | 627.694(452.871-863.698) | 1637580.218(1221669.756-2144780.205) | 498.091(288.461-771.102) | 1.709(1.103 - 2.51) | 0.109(0.031-0.187) |
| Burundi | 11810.822(8035.352-16433.192) | 704.178(479.079-979.771) | 80262.305(50639.603-121942.346) | 568.437(304.543-905.006) | 2.241(1.467 - 3.196) | 0.265(0.106-0.425) |
| Cabo Verde | 705.233(490.155-961.202) | 617.698(429.316-841.896) | 10662.698(6929.980-16007.719) | 577.294(331.113-946.016) | 0.645(0.273 - 1.037) | 0.161(-0.039-0.362) |
| Cambodia | 22423.981(15725.593-31224.792) | 699.230(490.359-973.659) | 271.862(175.962-399.297) | 631.510(350.956-1004.543) | 0.871(0.422 - 1.471) | 0.169(-0.011-0.350) |
| Cameroon | 20370.141(14511.576-28031.655) | 631.799(450.090-869.428) | 3822.442(2375.282-5754.362) | 522.315(293.948-834.049) | 2.654(1.734 - 3.625) | 0.134(0.030-0.237) |
| Canada | 50255.239(38805.102-61635.774) | 862.216(665.769-1057.469) | 142579.464(89867.064-212070.183) | 625.239(375.121-972.497) | 0.359(-0.135 - 1.002) | 0.478(-0.022-0.980) |
| Central African Republic | 6268.511(4298.702-8744.112) | 744.293(510.408-1038.234) | 3631.811(2266.549-5309.119) | 573.878(315.021-929.172) | 1.424(0.872 - 2.155) | 0.243(0.111-0.374) |
| Chad | 11633.857(8379.899-15932.521) | 637.249(459.012-872.709) | 2205.189(1437.939-3234.671) | 504.419(276.890-797.953) | 2.47(1.662 - 3.402) | 0.202(0.108-0.296) |
| Chile | 36250.370(27779.417-46869.745) | 964.769(739.323-1247.393) | 118420.490(74846.321-176606.015) | 995.496(562.059-1540.363) | 0.417(-0.013 - 0.931) | 0.540(0.326-0.755) |
| China | 2356519.397(1692861.420-3096256.201) | 652.850(468.991-857.787) | 15598.997(10846.991-22220.208) | 546.599(351.113-809.883) | -0.305(-0.357 - -0.249) | -0.443(-0.675 - -0.210 |
| Colombia | 90547.845(62569.489-127593.911) | 884.387(611.120-1246.218) | 50275.650(35644.407-66033.891) | 855.410(468.220-1376.720) | 0.497(0.134 - 0.945) | -0.156(-0.571-0.261) |
| Comoros | 1071.876(730.089-1529.648) | 695.678(473.848-992.784) | 2041.321(1299.025-3153.698) | 567.991(324.049-927.613) | 0.808(0.398 - 1.414) | 0.261(0.094-0.428) |
| Congo | 5814.622(4046.826-8186.438) | 713.654(496.685-1004.758) | 195501.219(128504.946-283625.975) | 568.841(327.702-948.555) | 1.422(0.879 - 2.128) | 0.193(0.046-0.341) |
| Cook Islands | 42.865(30.542-58.812) | 726.710(517.795-997.067) | 190.152(126.665-270.105) | 662.133(376.697-1061.240) | -0.164(-0.35 - 0.046) | 0.199(0.081-0.318) |
| Costa Rica | 7384.574(5176.243-10403.455) | 801.693(561.950-1129.433) | 895.509(594.996-1383.239) | 774.567(432.099-1214.067) | 0.566(0.192 - 0.94) | 0.315(0.101-0.530) |
| Croatia | 6595.476(4550.844-9426.672) | 638.401(440.494-912.444) | 5081.198(3213.119-7702.440) | 632.766(359.606-1042.541) | -0.111(-0.323 - 0.15) | 0.322(0.070-0.575) |
| Cuba | 24442.096(16131.279-34964.863) | 785.884(518.667-1124.221) | 13925.977(8430.174-20186.052) | 761.535(433.068-1218.442) | -0.15(-0.346 - 0.094) | 0.272(0.066-0.478) |
| Cyprus | 2301.984(1674.678-3148.209) | 1210.531(880.653-1655.530) | 26846.917(17605.706-39367.568) | 1169.438(654.001-1905.876) | 0.356(0.063 - 0.738) | 0.073(-0.091-0.238) |
| Czechia | 14647.201(10146.818-20861.963) | 616.532(427.101-878.124) | 2480.057(1585.354-3791.114) | 595.247(336.170-939.907) | -0.079(-0.288 - 0.187) | 0.309(0.046-0.573) |
| C么te d'Ivoire | 24129.001(17089.015-33179.046) | 631.261(447.082-868.028) | 129674.693(95127.703-168684.210) | 506.788(285.927-816.894) | 1.481(0.876 - 2.243) | 0.114(0.019-0.208) |
| Democratic People's Republic of Korea | 37592.276(27014.083-50728.543) | 683.078(490.865-921.773) | 844.530(548.451-1258.591) | 597.136(342.116-943.467) | 0.038(-0.19 - 0.328) | -0.077(-0.141 - -0.012) |
| Democratic Republic of the Congo | 86578.269(59503.390-121946.318) | 720.941(495.487-1015.452) | 231274.438(168573.161-299991.624) | 533.949(311.664-860.801) | 1.75(1.099 - 2.555) | 0.159(0.034-0.284) |
| Denmark | 11719.121(8273.854-16203.470) | 1071.875(756.758-1482.030) | 5045.626(3235.786-7702.306) | 1050.770(621.053-1658.797) | 0.216(-0.043 - 0.534) | 0.326(0.155-0.497) |
| Djibouti | 1000.069(668.884-1443.112) | 680.157(454.914-981.474) | 12798.172(8137.443-19522.668) | 521.115(289.196-833.899) | 1.894(1.19 - 2.643) | 0.196(0.053-0.340) |
| Dominica | 181.184(123.965-257.791) | 794.106(543.323-1129.865) | 14252.174(9377.232-20752.993) | 743.824(426.060-1181.207) | -0.048(-0.286 - 0.204) | 0.275(0.082-0.468) |
| Dominican Republic | 19414.780(13226.943-27289.831) | 815.054(555.282-1145.658) | 16484.308(10583.383-24484.082) | 705.797(394.726-1113.481) | 0.446(0.107 - 0.805) | 0.263(0.061-0.466) |
| Ecuador | 30693.773(20775.550-43237.378) | 941.399(637.201-1326.120) | 23852.738(15794.819-35232.515) | 921.513(530.101-1457.726) | 1.103(0.63 - 1.714) | 0.460(0.139-0.782) |
| Egypt | 159005.096(114975.324-217814.535) | 924.394(668.422-1266.289) | 1155.793(728.658-1766.569) | 902.732(501.406-1451.369) | 1.203(0.705 - 1.786) | 0.464(0.145-0.783) |
| El Salvador | 14517.052(10158.271-20456.660) | 817.056(571.733-1151.353) | 62821.543(38629.322-92950.564) | 770.757(432.932-1188.326) | 0.272(-0.018 - 0.668) | 0.330(0.112-0.549) |
| Equa-rial Guinea | 938.090(645.623-1318.797) | 719.594(495.248-1011.629) | 195507.633(130145.073-286823.669) | 540.371(312.479-881.136) | 3.704(2.585 - 4.942) | 0.100(-0.032-0.232) |
| Eritrea | 8031.613(5437.065-11386.398) | 712.694(482.464-1010.385) | 3144.878(1925.485-4811.926) | 538.189(301.712-869.021) | 1.151(0.595 - 1.733) | 0.158(0.059-0.256) |
| Es-nia | 2253.380(1551.904-3185.045) | 689.879(475.120-975.111) | 784.603(481.808-1162.940) | 711.263(403.807-1137.110) | -0.094(-0.31 - 0.177) | 0.240(-0.041-0.522) |
| Eswatini | 1908.577(1317.957-2660.852) | 692.751(478.376-965.803) | 1463.271(981.563-2138.940) | 761.967(440.474-1218.835) | 1.128(0.589 - 1.723) | 0.414(0.078-0.750) |
| Ethiopia | 122301.638(86824.845-162424.970) | 760.213(539.693-1009.615) | 24282.794(16409.839-34788.029) | 540.521(346.764-799.752) | 1.677(1.296 - 2.093) | -0.020(-0.199-0.160) |
| Fiji | 1755.849(1253.194-2410.291) | 738.146(526.833-1013.268) | 13484.748(8523.149-20375.198) | 687.688(380.475-1120.882) | 0.256(-0.033 - 0.608) | 0.159(-0.006-0.325) |
| Finland | 9839.043(7168.521-12904.679) | 1010.740(736.404-1325.665) | 16396.682(10803.413-23723.579) | 831.283(459.705-1274.868) | 0.067(-0.211 - 0.378) | 0.069(-0.160-0.300) |
| France | 155466.372(110521.402-209123.107) | 1229.823(874.284-1654.277) | 28332.498(17101.532-43235.135) | 1290.823(719.318-2014.997) | 0.258(-0.009 - 0.571) | 0.449(0.239-0.659) |
| Gabon | 2246.651(1563.902-3178.205) | 731.542(509.229-1034.869) | 5696.118(3482.365-8504.907) | 620.896(359.833-979.925) | 1.291(0.77 - 1.977) | 0.249(0.086-0.412) |
| Gambia | 1994.390(1433.170-2756.085) | 637.084(457.809-880.398) | 20972.941(13407.800-31390.065) | 553.445(309.032-860.007) | 2.083(1.285 - 2.831) | 0.189(0.047-0.332) |
| Georgia | 5831.036(4045.050-8353.192) | 454.004(314.947-650.378) | 7894.466(5137.546-11784.064) | 440.148(241.171-714.277) | -0.306(-0.465 - -0.124) | 0.232(-0.037-0.503) |
| Germany | 166106.256(114907.350-228504.092) | 1115.141(771.421-1534.043) | 59762.971(39918.384-85389.958) | 1260.615(723.569-2012.922) | 0.177(-0.114 - 0.507) | 0.425(0.239-0.612) |
| Ghana | 28798.212(20193.727-40101.860) | 611.649(428.897-851.729) | 58259.565(37495.735-87865.567) | 516.697(278.843-807.185) | 1.583(0.971 - 2.254) | 0.162(0.038-0.287) |
| Greece | 27029.913(18983.582-36263.393) | 1153.424(810.070-1547.436) | 46682.371(29729.620-68881.662) | 1234.121(726.037-1968.672) | -0.102(-0.313 - 0.149) | 0.333(0.073-0.593) |
| Greenland | 129.470(81.862-187.145) | 936.337(592.033-1353.444) | 3122.008(2018.276-4537.663) | 769.317(414.953-1223.846) | 0.113(-0.149 - 0.447) | 0.478(0.231-0.727) |
| Grenada | 209.046(143.440-294.885) | 795.717(545.991-1122.457) | 15318.103(11096.208-20086.114) | 739.101(416.396-1174.508) | 0.25(-0.052 - 0.628) | 0.248(0.062-0.434) |
| Guam | 278.302(193.099-377.862) | 709.375(492.198-963.147) | 10498.037(6707.306-15660.002) | 695.779(395.916-1086.380) | 0.192(-0.092 - 0.552) | 0.283(0.099-0.467) |
| Guatemala | 21829.248(15197.686-30457.913) | 818.385(569.766-1141.876) | 140935.070(89344.576-207916.415) | 768.959(443.502-1240.499) | 1.402(0.846 - 2.038) | 0.323(0.105-0.542) |
| Guinea | 10785.609(7717.213-14856.575) | 643.806(460.650-886.807) | 93956.881(62830.275-135115.142) | 555.139(319.200-909.973) | 2.064(1.38 - 2.969) | 0.182(0.046-0.319) |
| Guinea-Bissau | 2019.767(1451.107-2770.675) | 624.776(448.872-857.055) | 9761.098(6170.768-14731.278) | 526.695(300.938-850.470) | 1.42(0.881 - 2.038) | 0.150(0.007-0.294) |
| Guyana | 2090.954(1417.886-2988.284) | 806.473(546.873-1152.569) | 2367.185(1521.309-3570.847) | 809.618(446.321-1328.401) | 0.132(-0.117 - 0.433) | 0.320(0.076-0.564) |
| Haiti | 15924.411(11093.020-22267.804) | 820.425(571.512-1147.236) | 1074.128(662.859-1607.094) | 715.020(408.197-1158.019) | 1.396(0.845 - 2.028) | 0.233(0.061-0.404) |
| Honduras | 12228.912(8593.020-17208.945) | 797.665(560.504-1122.502) | 20514.161(13536.766-29816.521) | 825.528(466.041-1353.342) | 1.974(1.275 - 2.813) | 0.413(0.132-0.695) |
| Hungary | 14636.025(10133.330-20692.064) | 634.918(439.589-897.632) | 7423.445(4629.334-11423.243) | 623.334(342.449-1009.458) | -0.126(-0.317 - 0.083) | 0.288(0.044-0.533) |
| Iceland | 700.563(504.484-954.155) | 1108.187(798.020-1509.333) | 129634.711(93013.331-168226.885) | 961.297(529.125-1470.214) | 0.206(-0.09 - 0.51) | 0.130(0.020-0.239) |
| India | 1371121.945(961572.778-1825052.640) | 531.926(373.042-708.028) | 68312.814(42614.002-102183.495) | 388.835(248.446-553.701) | 0.891(0.776 - 1.018) | 0.362(0.033-0.693) |
| Indonesia | 377526.914(269713.407-493429.255) | 635.007(453.663-829.957) | 1317.419(838.952-1969.942) | 581.397(383.893-864.536) | 0.587(0.47 - 0.696) | 0.295(0.071-0.519) |
| Iran (Islamic Republic of) | 242189.091(179145.031-315937.583) | 1314.228(972.122-1714.421) | 64561.558(40313.751-97290.947) | 1341.749(861.729-1951.894) | 0.225(0.146 - 0.31) | 0.289(0.057-0.521) |
| Iraq | 62153.061(45308.642-85099.668) | 1014.652(739.666-1389.256) | 51361.174(32690.742-75122.828) | 985.877(558.268-1572.685) | 1.474(0.894 - 2.158) | 0.432(0.224-0.641) |
| Ireland | 12647.378(9239.901-17097.151) | 1298.520(948.671-1755.383) | 276.294(171.191-411.282) | 1329.272(768.882-2114.460) | 0.296(0.013 - 0.646) | 0.642(0.387-0.898) |
| Israel | 12000.683(8482.383-16370.977) | 881.171(622.833-1202.067) | 172.417(106.417-252.603) | 751.509(428.001-1197.628) | 0.988(0.513 - 1.507) | 0.416(0.091-0.741) |
| Italy | 144708.351(104417.346-189419.971) | 1156.153(834.247-1513.379) | 3100.399(1917.970-4649.263) | 1142.245(751.623-1631.333) | -0.104(-0.175 - -0.024) | 0.546(0.189-0.905) |
| Jamaica | 5927.609(4071.335-8340.065) | 776.460(533.306-1092.469) | 261.377(164.161-399.130) | 750.206(408.741-1195.268) | 0.252(-0.056 - 0.615) | 0.276(0.066-0.486) |
| Japan | 164995.861(117690.684-214683.609) | 587.499(419.060-764.422) | 30840.500(21089.872-45233.656) | 531.091(346.357-768.061) | -0.214(-0.258 - -0.167) | -0.134(-0.390-0.123) |
| Jordan | 13589.283(9779.533-18754.511) | 1002.447(721.411-1383.473) | 202.030(127.557-303.878) | 980.930(526.700-1560.694) | 2.423(1.668 - 3.329) | 0.289(0.142-0.436) |
| Kazakhstan | 18457.173(12997.459-26151.211) | 422.535(297.547-598.673) | 613.840(388.725-921.593) | 357.034(206.278-559.544) | 0.199(-0.08 - 0.546) | 0.208(0.016-0.400) |
| Kenya | 55366.856(40261.227-72720.098) | 698.141(507.669-916.954) | 38159.072(24273.067-58822.547) | 524.034(337.070-753.214) | 1.646(1.518 - 1.791) | 0.255(0.090-0.421) |
| Kiribati | 162.933(115.246-226.427) | 729.098(515.707-1013.227) | 20783.384(13416.833-31599.519) | 654.329(367.389-1001.854) | 0.918(0.463 - 1.453) | 0.143(0.015-0.272) |
| Kuwait | 4437.597(3108.680-6247.757) | 962.909(674.549-1355.693) | 50459.941(31881.471-75335.138) | 856.843(479.665-1396.118) | 1.01(0.577 - 1.6) | 0.244(0.115-0.372) |
| Kyrgyzstan | 5404.559(3736.597-7677.494) | 418.305(289.207-594.227) | 23259.910(16473.656-32803.999) | 377.307(205.449-603.967) | 0.858(0.402 - 1.365) | 0.298(0.038-0.559) |
| Lao People's Democratic Republic | 9869.489(6801.047-13747.557) | 765.120(527.243-1065.763) | 154494.708(113392.315-200330.956) | 682.281(367.862-1055.435) | 0.995(0.516 - 1.615) | 0.246(0.118-0.375) |
| Latvia | 3852.373(2668.746-5478.148) | 713.847(494.520-1015.104) | 135917.387(81913.219-213489.440) | 828.233(465.124-1319.334) | -0.184(-0.395 - 0.055) | 0.320(-0.026-0.666) |
| Lebanon | 9957.536(7089.156-13689.050) | 1147.549(816.985-1577.585) | 399.944(255.276-603.068) | 1433.337(809.464-2211.335) | 1.057(0.613 - 1.655) | 0.512(0.210-0.815) |
| Lesotho | 3327.355(2342.756-4657.481) | 696.402(490.329-974.792) | 916500.069(632727.334-1211949.752) | 734.497(429.270-1162.272) | 0.944(0.475 - 1.59) | 0.337(0.045-0.631) |
| Liberia | 4703.516(3369.681-6451.973) | 646.930(463.472-887.416) | 1628.412(1027.872-2415.707) | 525.548(292.478-865.134) | 1.758(1.071 - 2.459) | 0.102(-0.008-0.212) |
| Libya | 15425.296(11193.696-21038.267) | 1048.364(760.768-1429.844) | 29088.031(18537.562-43318.394) | 1065.467(603.854-1709.310) | 0.47(0.146 - 0.854) | 0.255(0.054-0.457) |
| Lithuania | 6387.337(4406.111-9050.722) | 780.683(538.530-1106.211) | 36365.397(22657.027-56002.300) | 928.797(526.478-1481.887) | -0.204(-0.393 - -0.003) | 0.306(-0.020-0.633) |
| Luxembourg | 795.270(565.167-1098.616) | 1100.885(782.355-1520.802) | 28079.057(16932.230-42650.689) | 1075.119(615.043-1709.530) | 0.84(0.409 - 1.302) | 0.265(0.087-0.443) |
| Madagascar | 27914.957(19014.836-39832.356) | 728.913(496.514-1040.099) | 90168.275(56881.431-132770.130) | 583.640(321.282-957.425) | 2.1(1.313 - 3.022) | 0.246(0.088-0.405) |
| Malawi | 22832.417(15546.775-32772.629) | 722.545(491.987-1037.109) | 8918.312(5898.862-12975.193) | 593.035(330.826-950.746) | 1.861(1.134 - 2.721) | 0.223(0.059-0.387) |
| Malaysia | 40642.497(28109.057-57824.316) | 756.617(523.290-1076.481) | 20102.618(13199.223-29898.359) | 720.083(412.395-1139.341) | 0.975(0.472 - 1.563) | 0.197(0.015-0.380) |
| Maldives | 468.632(320.779-652.430) | 654.394(447.933-911.047) | 11560.692(7508.280-17461.234) | 548.595(301.988-872.707) | 0.69(0.293 - 1.159) | 0.067(-0.106-0.241) |
| Mali | 15023.796(10701.840-20584.633) | 588.875(419.471-806.838) | 318564.812(196826.574-483850.560) | 480.274(275.144-804.980) | 2.594(1.71 - 3.592) | 0.203(0.075-0.331) |
| Malta | 1004.534(720.406-1369.528) | 1227.574(880.360-1673.609) | 4167.640(2767.386-6034.548) | 1133.380(662.684-1793.040) | -0.109(-0.308 - 0.135) | 0.099(-0.066-0.264) |
| Marshall Islands | 113.937(82.148-153.709) | 747.376(538.852-1008.262) | 37999.305(25629.283-56705.394) | 649.208(366.016-1050.163) | 0.266(-0.007 - 0.644) | 0.079(-0.040-0.198) |
| Mauritania | 3770.447(2667.196-5204.216) | 592.273(418.971-817.494) | 22669.338(14574.475-33804.944) | 489.274(273.280-780.273) | 1.606(0.956 - 2.346) | 0.173(0.048-0.298) |
| Mauritius | 2139.144(1488.358-3019.454) | 659.169(458.632-930.433) | 54653.591(35940.564-80296.886) | 593.350(337.959-968.047) | 0.025(-0.226 - 0.291) | 0.221(0.035-0.408) |
| Mexico | 165098.234(118889.358-214949.243) | 568.153(409.135-739.706) | 2593061.766(1771219.001-3464174.415) | 680.759(435.594-966.802) | 1.007(0.847 - 1.165) | 1.398(1.065-1.732) |
| Micronesia (Federated States of) | 256.807(185.393-346.985) | 747.459(539.603-1009.931) | 22238.470(14593.642-32483.986) | 649.439(362.442-997.794) | 0.053(-0.178 - 0.372) | 0.068(-0.046-0.182) |
| Monaco | 47.589(33.356-66.670) | 1095.159(767.605-1534.253) | 13682.817(8835.660-20865.180) | 1148.522(628.267-1778.494) | 0.616(0.22 - 1.165) | 0.361(0.187-0.536) |
| Mongolia | 2997.522(2069.670-4202.174) | 423.146(292.166-593.202) | 52433.535(32355.905-79232.473) | 317.896(182.176-517.757) | 0.212(-0.08 - 0.582) | -0.199(-0.447-0.050) |
| Montenegro | 1021.982(708.449-1441.041) | 644.459(446.746-908.715) | 4687.013(2989.862-6954.882) | 701.375(389.969-1145.397) | 0.131(-0.15 - 0.428) | 0.369(0.048-0.691) |
| Morocco | 80552.334(57809.937-111164.596) | 994.985(714.070-1373.109) | 296617.793(221031.336-394006.611) | 1028.559(585.608-1621.387) | 0.499(0.175 - 0.94) | 0.360(0.113-0.608) |
| Mozambique | 30446.258(20735.053-42941.701) | 736.760(501.762-1039.134) | 46512.738(29649.259-67498.994) | 599.794(347.523-952.356) | 2.267(1.515 - 3.093) | 0.221(0.056-0.385) |
| Myanmar | 86465.052(60376.886-123599.857) | 661.890(462.185-946.157) | 494677.065(335483.347-665805.236) | 645.213(353.599-1046.454) | 0.649(0.266 - 1.125) | 0.309(0.070-0.549) |
| Namibia | 3237.051(2240.603-4488.723) | 682.138(472.158-945.900) | 773464.767(534397.554-1042261.277) | 736.323(420.737-1132.974) | 1.441(0.893 - 2.118) | 0.378(0.081-0.675) |
| Nauru | 22.296(16.048-30.870) | 736.853(530.354-1020.202) | 331407.517(238872.843-441385.584) | 649.866(369.695-1070.919) | 0.334(0.009 - 0.674) | 0.135(0.019-0.251) |
| Nepal | 31520.493(20910.264-45516.636) | 529.182(351.052-764.157) | 11041.812(6868.212-16543.608) | 455.366(251.711-754.431) | 1.246(0.72 - 1.967) | 0.495(0.251-0.739) |
| Netherlands | 37684.202(26118.559-52210.558) | 1150.350(797.297-1593.783) | 140474.627(94396.082-209566.465) | 1332.283(768.788-2105.531) | 0.239(-0.058 - 0.558) | 0.397(0.183-0.611) |
| New Zealand | 11432.697(7909.103-15424.178) | 1359.410(940.435-1834.019) | 61745.262(40336.887-90528.400) | 1054.800(652.366-1567.074) | 0.364(0.1 - 0.641) | 0.002(-0.129-0.133) |
| Nicaragua | 10807.892(7557.072-15104.692) | 823.811(576.024-1151.327) | 135554.107(85153.147-203038.422) | 775.642(450.484-1254.751) | 0.86(0.427 - 1.356) | 0.341(0.106-0.576) |
| Niger | 14812.000(10609.200-20356.782) | 605.173(433.459-831.715) | 18466.201(11642.647-27888.433) | 460.428(242.061-728.792) | 2.636(1.788 - 3.716) | 0.161(0.098-0.225) |
| Nigeria | 175752.364(127910.413-227593.683) | 622.671(453.172-806.339) | 20484.482(13285.941-29297.434) | 463.221(302.391-675.848) | 1.945(1.795 - 2.112) | 0.175(0.051-0.299) |
| Niue | 4.674(3.420-6.346) | 729.500(533.754-990.411) | 119688.868(77579.806-176526.642) | 645.612(350.569-1035.246) | -0.27(-0.445 - -0.056) | 0.117(0.007-0.226) |
| North Macedonia | 3286.445(2263.057-4666.212) | 643.620(443.199-913.835) | 1179.632(672.172-1771.816) | 740.255(406.411-1168.224) | 0.163(-0.114 - 0.479) | 0.442(0.070-0.815) |
| Northern Mariana Islands | 92.133(63.931-130.091) | 727.110(504.540-1026.680) | 120734.314(77895.478-177328.268) | 713.593(404.273-1125.857) | 0.194(-0.095 - 0.54) | 0.295(0.120-0.469) |
| Norway | 12952.696(9246.885-17363.143) | 1412.920(1008.679-1894.025) | 91673.848(59537.710-137757.261) | 1229.138(786.150-1775.352) | 0.183(0.067 - 0.324) | -0.019(-0.149-0.112) |
| Oman | 5500.949(4008.975-7436.821) | 1012.046(737.557-1368.201) | 231703.831(147693.534-331317.850) | 1006.588(563.920-1611.295) | 1.155(0.669 - 1.692) | 0.126(-0.118-0.371) |
| Pakistan | 198884.467(141323.707-265246.539) | 558.352(396.755-744.658) | 350294.490(223201.929-525015.476) | 398.373(245.546-600.733) | 1.487(1.128 - 1.917) | 0.320(0.159-0.482) |
| Palau | 33.672(23.826-46.272) | 718.995(508.758-988.038) | 70784.252(43733.519-110660.636) | 641.224(355.372-1012.051) | -0.112(-0.325 - 0.151) | 0.119(0.004-0.235) |
| Palestine | 7189.932(5219.711-9896.236) | 1058.711(768.597-1457.211) | 122705.972(80157.888-180357.155) | 1077.297(597.063-1760.148) | 2.093(1.347 - 2.975) | 0.270(0.092-0.449) |
| Panama | 5707.507(4010.189-8127.380) | 755.293(530.681-1075.523) | 2894.155(1784.856-4507.985) | 713.036(394.041-1108.855) | 0.935(0.443 - 1.516) | 0.403(0.147-0.660) |
| Papua New Guinea | 9583.414(6886.933-13269.383) | 731.863(525.939-1013.352) | 14084.830(8741.932-20948.944) | 612.032(324.627-996.341) | 1.625(0.955 - 2.386) | 0.068(-0.020-0.156) |
| Paraguay | 13380.583(9323.890-18662.328) | 1084.657(755.814-1512.806) | 99473.971(62246.285-150306.645) | 1087.711(620.923-1745.482) | 1.174(0.688 - 1.76) | 0.297(0.083-0.512) |
| Peru | 67768.623(45839.565-97825.406) | 961.770(650.554-1388.335) | 153743.819(101723.288-232223.757) | 1045.826(596.697-1664.035) | 1.006(0.527 - 1.517) | 0.526(0.130-0.924) |
| Philippines | 146151.267(105469.014-190245.431) | 713.696(515.034-929.020) | 6468.822(4220.507-9673.491) | 641.229(418.084-930.519) | 1.115(1.013 - 1.228) | 0.281(0.052-0.511) |
| Poland | 52562.543(38435.648-68572.852) | 613.099(448.320-799.846) | 4412.532(2710.095-6739.685) | 582.217(374.726-854.293) | -0.044(-0.116 - 0.027) | 0.323(0.062-0.586) |
| Portugal | 35599.418(25617.278-48669.249) | 1427.024(1026.884-1950.936) | 49845.385(33046.003-73935.059) | 1628.753(901.996-2580.122) | -0.134(-0.307 - 0.086) | 0.438(0.209-0.668) |
| Puer- Rico | 7846.220(5478.545-10997.001) | 798.531(557.566-1119.194) | 11852.240(7728.212-16852.793) | 715.241(391.887-1128.687) | -0.231(-0.423 - -0.027) | 0.190(0.005-0.375) |
| Qatar | 936.806(671.109-1279.086) | 949.870(680.468-1296.923) | 196.621(126.316-294.226) | 843.999(483.433-1308.048) | 4.003(2.833 - 5.374) | -0.265(-0.504 - -0.027) |
| Republic of Korea | 88163.588(62456.151-122420.495) | 670.810(475.210-931.460) | 327418.343(228172.527-434886.202) | 578.382(321.202-963.072) | -0.339(-0.493 - -0.16) | 0.129(0.025-0.232) |
| Republic of Moldova | 7645.234(5384.019-10850.837) | 743.937(523.904-1055.865) | 147316.106(103434.003-196812.385) | 760.504(434.605-1202.558) | -0.255(-0.436 - -0.035) | 0.242(-0.046-0.531) |
| Romania | 36025.420(24687.280-51535.995) | 610.599(418.427-873.489) | 169652.046(105453.252-263130.979) | 635.022(358.972-995.280) | -0.214(-0.411 - -0.002) | 0.387(0.060-0.715) |
| Russian Federation | 211271.966(153205.274-274176.508) | 671.748(487.123-871.756) | 1160.010(765.914-1745.751) | 662.424(431.458-951.280) | 0.095(0.046 - 0.146) | 0.303(0.019-0.589) |
| Rwanda | 15886.315(10645.452-22569.405) | 701.645(470.174-996.815) | 86550.023(52702.405-129487.350) | 573.176(324.884-950.527) | 1.416(0.884 - 2.092) | 0.239(0.086-0.393) |
| Saint Kitts and Nevis | 101.374(69.807-143.301) | 798.380(549.773-1128.584) | 12971.205(8700.883-18996.804) | 720.118(404.533-1147.196) | 0.19(-0.093 - 0.506) | 0.237(0.072-0.403) |
| Saint Lucia | 364.162(251.824-516.738) | 802.960(555.261-1139.384) | 238085.998(153150.767-362245.378) | 789.913(428.101-1277.730) | 0.098(-0.181 - 0.455) | 0.258(0.032-0.484) |
| Saint Vincent and the Grenadines | 297.946(206.946-421.796) | 796.655(553.338-1127.809) | 15194.605(9511.342-23072.685) | 739.452(421.632-1165.587) | -0.073(-0.284 - 0.214) | 0.239(0.048-0.431) |
| Samoa | 430.286(310.801-594.083) | 729.321(526.797-1006.951) | 43589.157(28220.536-65492.158) | 649.355(364.193-1060.280) | 0.274(-0.02 - 0.626) | 0.191(0.077-0.305) |
| San Marino | 62.424(43.681-86.356) | 1120.440(784.015-1549.988) | 59869.069(38051.477-90135.333) | 1167.888(658.384-1869.302) | 0.284(0.011 - 0.627) | 0.459(0.233-0.685) |
| Sao -me and Principe | 252.248(181.706-342.491) | 623.640(449.238-846.752) | 1937.733(1188.100-3016.213) | 507.042(297.749-811.640) | 0.947(0.484 - 1.503) | 0.138(0.022-0.254) |
| Saudi Arabia | 49170.937(35597.199-67674.959) | 974.972(705.829-1341.874) | 2192.024(1363.605-3337.308) | 888.466(497.194-1421.758) | 0.834(0.387 - 1.383) | 0.159(-0.002-0.320) |
| Senegal | 14788.840(10481.512-20118.132) | 610.512(432.697-830.515) | 63599.281(39236.559-98646.006) | 551.505(301.046-913.859) | 1.724(1.089 - 2.574) | 0.210(0.009-0.411) |
| Serbia | 13620.868(9443.785-19215.490) | 622.856(431.846-878.688) | 5147.740(3224.798-7916.231) | 610.404(341.395-998.853) | 0.022(-0.217 - 0.333) | 0.309(0.065-0.554) |
| Seychelles | 144.568(98.449-202.378) | 647.723(441.094-906.736) | 38385.865(24318.009-59106.998) | 617.570(349.036-998.441) | 0.36(0.036 - 0.742) | 0.243(0.017-0.470) |
| Sierra Leone | 8010.418(5608.164-11113.351) | 658.662(461.135-913.802) | 17272.700(10537.103-25658.222) | 513.477(295.163-837.157) | 1.493(0.876 - 2.219) | 0.102(0.012-0.191) |
| Singapore | 4952.464(3461.166-6875.659) | 600.553(419.713-833.767) | 31136.806(19882.138-47249.626) | 513.155(293.501-835.847) | 0.019(-0.222 - 0.283) | 0.189(0.081-0.298) |
| Slovakia | 8081.081(5670.819-11456.893) | 634.955(445.573-900.202) | 74379.062(47828.519-112112.870) | 667.872(370.270-1059.444) | -0.023(-0.246 - 0.252) | 0.304(0.024-0.585) |
| Slovenia | 2788.392(1898.693-3907.493) | 629.237(428.465-881.777) | 6474.510(4040.354-10000.858) | 587.523(312.127-964.164) | -0.111(-0.313 - 0.118) | 0.278(0.032-0.524) |
| Solomon Islands | 850.431(594.953-1172.090) | 737.310(515.815-1016.184) | 146514.044(103851.788-193563.468) | 648.980(362.757-1067.413) | 1.171(0.698 - 1.875) | 0.159(0.042-0.276) |
| Somalia | 17291.943(11887.960-24378.434) | 678.928(466.753-957.163) | 4887.035(3197.155-7352.226) | 572.440(326.364-897.138) | 2.844(1.932 - 4.01) | 0.243(0.039-0.447) |
| South Africa | 86249.977(62035.657-113920.624) | 730.124(525.145-964.362) | 74430.970(47722.233-109468.485) | 699.582(451.805-1014.147) | 0.708(0.533 - 0.903) | 0.358(0.084-0.633) |
| South Sudan | 15704.821(10592.230-22213.818) | 791.280(533.684-1119.232) | 66473.616(40813.415-99517.157) | 584.344(325.736-936.950) | 0.936(0.459 - 1.469) | 0.198(0.086-0.310) |
| Spain | 94870.613(67146.207-129683.717) | 974.063(689.409-1331.499) | 138166.338(89409.071-208130.866) | 1023.666(589.561-1559.738) | -0.01(-0.245 - 0.265) | 0.809(0.461-1.158) |
| Sri Lanka | 34189.694(23577.226-47965.895) | 660.144(455.236-926.139) | 65324.843(40435.582-97399.153) | 619.278(342.357-1036.107) | 0.362(0.015 - 0.772) | 0.282(0.086-0.479) |
| Sudan | 65131.199(46559.221-88902.206) | 1008.320(720.801-1376.328) | 9824.562(6170.378-15594.008) | 974.488(570.905-1599.244) | 1.739(1.09 - 2.575) | 0.299(0.112-0.486) |
| Suriname | 956.664(651.885-1368.516) | 782.983(533.536-1120.063) | 19966.080(12849.167-29900.944) | 801.388(442.553-1286.754) | 0.702(0.248 - 1.223) | 0.331(0.065-0.598) |
| Sweden | 17707.476(12837.807-23882.267) | 1069.583(775.441-1442.559) | 4061.099(2676.975-6131.559) | 978.425(600.624-1442.897) | 0.314(0.071 - 0.61) | 0.271(0.088-0.455) |
| Switzerland | 16909.933(12074.199-23537.270) | 1271.780(908.089-1770.216) | 7901.948(5119.030-11686.442) | 1252.772(714.399-1965.368) | 0.213(-0.077 - 0.516) | 0.323(0.214-0.431) |
| Syrian Arab Republic | 45904.664(33215.422-62421.158) | 1059.403(766.556-1440.576) | 38272.982(24360.329-57586.083) | 1084.481(619.637-1696.452) | 0.345(0.047 - 0.695) | 0.413(0.230-0.596) |
| Taiwan (Province of China) | 44278.553(33124.153-59370.687) | 774.453(579.357-1038.421) | 53992.828(35265.117-80902.459) | 607.407(340.377-1028.538) | -0.434(-0.592 - -0.263) | -0.383(-0.489 - -0.277) |
| Tajikistan | 7356.352(5070.167-10290.298) | 443.782(305.865-620.777) | 40369.111(26587.011-60253.285) | 410.399(227.325-650.859) | 1.281(0.701 - 2.011) | 0.266(0.034-0.499) |
| Thailand | 110871.685(76937.348-159369.224) | 618.768(429.382-889.430) | 331.635(216.784-489.288) | 515.334(299.209-848.130) | -0.212(-0.406 - 0.027) | 0.164(0.045-0.284) |
| Timor-Leste | 1484.650(1018.875-2079.705) | 644.920(442.591-903.406) | 6149.021(3944.931-9207.816) | 561.986(309.098-906.510) | 1.601(0.983 - 2.319) | 0.251(0.106-0.396) |
| -go | 7841.209(5644.317-10876.867) | 656.376(472.478-910.487) | 33046.965(21770.811-48742.497) | 532.144(300.122-826.735) | 1.422(0.843 - 2.076) | 0.124(0.029-0.220) |
| -kelau | 3.355(2.427-4.591) | 733.342(530.474-1003.545) | 517551.919(377320.759-666594.231) | 640.458(356.630-1046.914) | -0.03(-0.241 - 0.24) | 0.094(-0.027-0.216) |
| -nga | 246.047(173.988-335.022) | 736.383(520.720-1002.672) | 30406.318(18889.832-45274.642) | 653.271(367.649-1036.978) | 0.105(-0.144 - 0.401) | 0.171(0.057-0.284) |
| Trinidad and -bago | 2623.518(1824.521-3730.397) | 764.341(531.559-1086.821) | 120.599(72.254-183.202) | 787.207(456.230-1278.709) | 0.182(-0.081 - 0.492) | 0.345(0.078-0.613) |
| Tunisia | 27239.968(19568.653-37516.701) | 1018.700(731.814-1403.021) | 96.769(60.656-148.843) | 1203.564(687.648-1848.098) | 0.395(0.068 - 0.79) | 0.369(0.081-0.659) |
| Turkey | 157356.342(122484.625-195577.890) | 857.262(667.284-1065.489) | 29.738(19.077-44.117) | 942.287(507.149-1455.504) | 0.472(0.022 - 1.01) | 0.275(0.050-0.500) |
| Turkmenistan | 4981.110(3466.881-7044.450) | 434.529(302.435-614.526) | 147.158(93.881-225.441) | 390.018(211.870-639.550) | 0.542(0.18 - 0.997) | 0.103(-0.010-0.217) |
| Tuvalu | 17.910(12.667-24.487) | 727.847(514.763-995.091) | 3.253(2.119-4.616) | 646.421(355.928-1054.214) | 0.67(0.285 - 1.164) | 0.585(0.388-0.782) |
| Uganda | 40897.033(27850.900-58856.208) | 723.894(492.972-1041.778) | 53860.486(34882.138-81739.711) | 585.284(335.684-904.732) | 2.378(1.572 - 3.34) | 0.288(0.125-0.451) |
| Ukraine | 68731.200(48681.035-91312.224) | 633.390(448.619-841.485) | 18993.959(12084.578-28888.770) | 608.174(367.633-949.078) | -0.13(-0.337 - 0.113) | 0.356(0.073-0.640) |
| United Arab Emirates | 3886.487(2824.241-5224.349) | 923.707(671.242-1241.679) | 109.963(70.032-166.580) | 956.868(537.699-1533.182) | 2.521(1.658 - 3.575) | 0.389(0.040-0.738) |
| United Kingdom | 114595.789(82556.098-150182.704) | 966.855(696.534-1267.106) | 144.042(87.279-211.398) | 937.281(603.486-1358.679) | 0.348(0.297 - 0.403) | 0.348(0.102-0.595) |
| United Republic of Tanzania | 61428.443(42161.085-87114.038) | 720.637(494.605-1021.963) | 40280.876(25509.843-60820.515) | 563.932(314.030-947.975) | 1.762(1.1 - 2.593) | 0.243(0.102-0.383) |
| United States of America | 537968.567(371182.282-723374.450) | 972.302(670.859-1307.397) | 178389.456(117766.151-270539.256) | 847.105(551.707-1233.463) | 0.704(0.601 - 0.807) | 0.436(0.106-0.766) |
| United States Virgin Islands | 225.667(156.669-319.423) | 803.073(557.532-1136.718) | 6035.269(3749.470-9259.529) | 746.895(416.700-1177.287) | -0.348(-0.501 - -0.167) | 0.285(0.080-0.490) |
| Uruguay | 7591.901(5379.711-10601.279) | 990.485(701.870-1383.107) | 122.514(76.919-182.904) | 1021.660(567.891-1591.851) | 0.286(-0.038 - 0.604) | 0.285(0.090-0.481) |
| Uzbekistan | 26671.996(18175.079-37517.089) | 417.502(284.498-587.262) | 80.138(52.712-115.565) | 342.188(190.625-562.800) | 0.531(0.155 - 0.998) | 0.169(-0.016-0.355) |
| Vanuatu | 348.819(243.164-481.937) | 737.123(513.853-1018.427) | 29.917(19.769-44.744) | 656.454(358.671-1035.665) | 1.361(0.811 - 2.061) | 0.142(0.021-0.262) |
| Venezuela (Bolivarian Republic of) | 46826.154(32804.503-65295.811) | 781.431(547.439-1089.651) | 76.910(49.561-113.979) | 657.019(375.557-1038.974) | 0.167(-0.085 - 0.505) | 0.239(0.115-0.363) |
| Viet Nam | 95352.895(66174.174-132132.285) | 442.610(307.168-613.333) | 491.234(314.981-713.141) | 355.756(200.240-589.155) | 0.242(-0.075 - 0.603) | 0.039(-0.304-0.384) |
| Yemen | 43637.867(31661.690-59118.556) | 1035.709(751.465-1403.131) | 3.414(2.236-5.121) | 852.668(479.647-1336.560) | 1.812(1.154 - 2.672) | 0.122(0.028-0.217) |
| Zambia | 20182.110(13687.413-28473.180) | 727.621(493.469-1026.536) | 35.818(23.155-54.883) | 618.936(341.910-1005.605) | 2.151(1.375 - 3.026) | 0.293(0.111-0.475) |
| Zimbabwe | 23334.080(16143.142-32673.163) | 649.668(449.458-909.687) | 29.901(19.127-44.144) | 580.117(322.407-917.602) | 0.868(0.419 - 1.402) | 0.242(0.052-0.432) |
| location | Rate per 100 000 (95% UI) | | | | | |
|  | 1990 | | 2021 | | 1990-2021 | |
|  | Prevalence cases | Prevalence rate | Prevalence cases | Prevalence rate | Cases change | EAPCa |
| Afghanistan | 220233.579(156516.575-301949.068) | 6521.169(4634.493-8940.784) | 897810.830(603617.996-1307708.737) | 8430.305(5667.880-12279.183) | 3.077(2.086 - 4.176) | 0.294(0.080-0.510) |
| Albania | 37129.285(25876.716-51837.921) | 3790.790(2641.936-5292.498) | 29272.296(19443.647-43402.958) | 5539.129(3679.277-8213.042) | -0.212(-0.383 - -0.002) | 0.468(0.183-0.754) |
| Algeria | 545081.760(388560.087-743706.067) | 6402.925(4564.308-8736.110) | 762114.424(505664.867-1131905.561) | 7497.222(4974.426-11135.004) | 0.398(0.055 - 0.821) | 0.265(0.135-0.395) |
| American Samoa | 666.791(470.031-916.452) | 4433.828(3125.470-6093.945) | 728.974(458.000-1094.803) | 5233.734(3288.251-7860.236) | 0.093(-0.149 - 0.362) | 0.153(0.041-0.265) |
| Andorra | 881.771(622.966-1216.428) | 7258.549(5128.126-10013.379) | 1216.913(806.748-1729.608) | 9341.744(6193.074-13277.490) | 0.38(0.073 - 0.744) | 0.296(0.058-0.535) |
| Angola | 129360.859(91627.706-179101.181) | 4037.050(2859.486-5589.329) | 500977.640(327588.514-744451.231) | 4744.609(3102.493-7050.474) | 2.873(2.02 - 3.954) | 0.163(0.028-0.297) |
| Antigua and Barbuda | 808.162(571.059-1106.957) | 4650.287(3285.963-6369.601) | 1188.228(769.334-1716.625) | 6034.075(3906.841-8717.390) | 0.47(0.137 - 0.859) | 0.261(0.068-0.454) |
| Argentina | 526378.441(372025.369-736593.987) | 6041.844(4270.158-8454.727) | 856962.004(549597.211-1234455.970) | 8015.145(5140.369-11545.837) | 0.628(0.24 - 1.063) | 0.140(-0.130-0.411) |
| Armenia | 24414.532(17386.989-33446.121) | 2803.532(1996.556-3840.633) | 22250.533(14113.462-32178.411) | 4129.010(2619.021-5971.316) | -0.089(-0.309 - 0.16) | 0.456(0.171-0.741) |
| Australia | 269401.077(194557.033-366250.053) | 6785.720(4900.535-9225.168) | 348249.977(228704.375-496661.794) | 7370.837(4840.611-10512.027) | 0.293(-0.021 - 0.655) | 0.358(0.202-0.514) |
| Austria | 123269.692(88913.254-165428.454) | 7579.147(5466.767-10171.248) | 138345.491(92286.101-203453.679) | 9792.111(6532.021-14400.476) | 0.122(-0.13 - 0.404) | 0.500(0.323-0.677) |
| Azerbaijan | 53563.922(37683.372-74838.988) | 2532.592(1781.733-3538.512) | 77907.749(51055.289-118561.384) | 3531.554(2314.334-5374.381) | 0.454(0.11 - 0.906) | 0.341(0.093-0.589) |
| Bahamas | 3742.400(2619.764-5134.213) | 4630.907(3241.739-6353.160) | 6194.868(4228.481-9052.422) | 6247.821(4264.626-9129.801) | 0.655(0.265 - 1.194) | 0.336(0.094-0.579) |
| Bahrain | 8765.423(6313.515-11887.241) | 6781.018(4884.198-9196.087) | 27787.425(18086.545-38799.746) | 8277.297(5387.606-11557.639) | 2.17(1.47 - 3.157) | 0.146(-0.025-0.317) |
| Bangladesh | 914356.294(646096.708-1265426.824) | 2619.914(1851.267-3625.840) | 1563147.288(1019259.123-2290313.451) | 3413.352(2225.696-5001.222) | 0.71(0.314 - 1.229) | 0.479(0.279-0.680) |
| Barbados | 3122.335(2207.747-4199.142) | 4622.635(3268.581-6216.853) | 3583.410(2358.054-5272.504) | 6273.297(4128.127-9230.310) | 0.148(-0.105 - 0.451) | 0.294(0.086-0.503) |
| Belarus | 90840.883(64474.352-125374.830) | 4102.840(2911.992-5662.570) | 94874.634(60846.445-138172.955) | 6672.651(4279.406-9717.875) | 0.044(-0.19 - 0.36) | 0.435(0.082-0.788) |
| Belgium | 138882.197(97969.091-187875.422) | 6899.003(4866.635-9332.751) | 167328.738(112537.989-244318.657) | 8517.971(5728.815-12437.190) | 0.205(-0.047 - 0.555) | 0.302(0.086-0.518) |
| Belize | 2794.660(1975.087-3875.446) | 4471.884(3160.442-6201.307) | 7615.151(4944.132-11033.520) | 5766.568(3743.941-8355.126) | 1.725(1.072 - 2.519) | 0.333(0.127-0.539) |
| Benin | 53776.712(37631.758-73769.165) | 3727.114(2608.152-5112.735) | 185505.789(118684.858-266530.539) | 4236.477(2710.458-6086.875) | 2.45(1.617 - 3.484) | 0.179(0.088-0.270) |
| Bermuda | 583.637(416.229-798.336) | 4685.147(3341.279-6408.645) | 560.847(374.716-824.192) | 6117.688(4087.384-8990.236) | -0.039(-0.268 - 0.238) | 0.270(0.070-0.470) |
| Bhutan | 6064.615(4277.529-8320.375) | 2665.400(1879.975-3656.807) | 5862.978(3686.065-8651.275) | 2864.212(1800.735-4226.365) | -0.033(-0.257 - 0.262) | 0.317(0.255-0.379) |
| Bolivia (Plurinational State of) | 109917.743(77962.581-150867.012) | 5485.764(3890.949-7529.456) | 288483.220(190509.506-412352.586) | 8918.255(5889.467-12747.589) | 1.625(1.044 - 2.349) | 0.647(0.233-1.062) |
| Bosnia and Herzegovina | 42608.711(30517.707-58089.968) | 3705.751(2654.176-5052.182) | 29548.617(19201.077-43284.384) | 5458.056(3546.716-7995.250) | -0.307(-0.472 - -0.126) | 0.384(0.104-0.665) |
| Botswana | 17547.905(12336.135-24078.622) | 3890.504(2735.015-5338.414) | 36935.356(23868.432-53633.525) | 5649.397(3650.763-8203.442) | 1.105(0.635 - 1.721) | 0.325(0.095-0.555) |
| Brazil | 2691183.768(2197263.203-3294265.410) | 5771.861(4712.535-7065.308) | 4526420.248(3606238.893-5648113.662) | 9316.268(7422.352-11624.934) | 0.682(0.539 - 0.821) | 0.672(0.364-0.981) |
| Brunei Darussalam | 2886.750(2041.826-3980.702) | 3789.157(2680.107-5225.081) | 4757.197(3011.895-7013.757) | 4485.717(2840.014-6613.500) | 0.648(0.256 - 1.141) | 0.150(0.024-0.276) |
| Bulgaria | 68721.247(48212.363-95333.216) | 3728.920(2616.076-5172.926) | 60689.352(39069.595-89117.173) | 6252.118(4024.886-9180.706) | -0.117(-0.339 - 0.167) | 0.471(0.091-0.853) |
| Burkina Faso | 107244.893(75341.346-149361.357) | 3658.911(2570.447-5095.813) | 295673.095(191788.622-442401.133) | 4067.299(2638.257-6085.700) | 1.757(1.144 - 2.55) | 0.160(0.083-0.237) |
| Burundi | 61390.752(43230.574-84049.341) | 3660.206(2577.470-5011.145) | 199510.368(131409.320-290785.063) | 4621.477(3043.978-6735.772) | 2.25(1.503 - 3.289) | 0.347(0.192-0.503) |
| Cabo Verde | 4184.181(2993.606-5779.686) | 3664.831(2622.033-5062.298) | 7039.161(4563.161-10372.652) | 4692.075(3041.654-6914.070) | 0.682(0.301 - 1.12) | 0.326(0.134-0.518) |
| Cambodia | 125490.425(88124.444-174518.347) | 3913.073(2747.918-5441.874) | 238117.655(154784.430-354119.327) | 5142.608(3342.867-7647.887) | 0.897(0.449 - 1.446) | 0.314(0.150-0.478) |
| Cameroon | 121184.490(84407.434-168266.437) | 3758.650(2617.976-5218.941) | 441823.630(288969.346-651784.119) | 4254.295(2782.469-6275.992) | 2.646(1.707 - 3.699) | 0.148(0.054-0.243) |
| Canada | 253200.715(215617.537-298916.992) | 4344.099(3699.294-5128.441) | 331266.895(218639.212-497467.713) | 5123.896(3381.818-7694.619) | 0.308(-0.091 - 0.868) | 0.347(-0.116-0.813) |
| Central African Republic | 35108.863(24748.521-48104.408) | 4168.660(2938.522-5711.689) | 85370.036(53302.462-126281.379) | 4704.886(2937.588-6959.578) | 1.432(0.884 - 2.113) | 0.273(0.140-0.407) |
| Chad | 68999.583(48382.724-96552.441) | 3779.476(2650.181-5288.694) | 240187.620(154512.712-361215.876) | 4117.350(2648.692-6192.044) | 2.481(1.633 - 3.402) | 0.200(0.105-0.294) |
| Chile | 233199.204(194454.577-277592.333) | 6206.373(5175.222-7387.854) | 320253.762(207523.377-461829.306) | 8160.704(5288.109-11768.331) | 0.373(-0.047 - 0.837) | 0.504(0.282-0.725) |
| China | 15536620.809(12457215.551-19308435.916) | 4304.268(3451.149-5349.212) | 10267532.346(8029994.904-12824840.318) | 4387.098(3431.046-5479.781) | -0.339(-0.38 - -0.297) | -0.293(-0.463 - -0.123) |
| Colombia | 548897.239(391584.383-750805.256) | 5361.115(3824.630-7333.164) | 831170.393(545396.873-1245809.876) | 6944.729(4556.988-10409.192) | 0.514(0.155 - 0.973) | -0.211(-0.682-0.263) |
| Comoros | 5552.839(3950.760-7583.641) | 3603.948(2564.154-4921.995) | 10204.206(6729.526-15317.046) | 4631.751(3054.573-6952.499) | 0.838(0.446 - 1.399) | 0.329(0.161-0.497) |
| Congo | 32020.489(22586.042-44254.946) | 3930.015(2772.084-5431.604) | 77755.772(51631.199-115188.000) | 4663.641(3096.740-6908.754) | 1.428(0.893 - 2.2) | 0.197(0.054-0.340) |
| Cook Islands | 259.209(185.402-358.889) | 4394.521(3143.223-6084.447) | 219.168(143.184-328.890) | 5367.634(3506.711-8054.833) | -0.154(-0.346 - 0.065) | 0.214(0.094-0.334) |
| Costa Rica | 43344.787(30605.430-58950.817) | 4705.651(3322.625-6399.892) | 68830.389(45602.584-100807.127) | 6293.905(4169.936-9217.883) | 0.588(0.2 - 0.998) | 0.419(0.212-0.626) |
| Croatia | 38174.967(26829.968-52642.814) | 3695.101(2596.975-5095.499) | 33956.450(21832.551-50510.026) | 5128.051(3297.118-7627.946) | -0.111(-0.328 - 0.148) | 0.341(0.100-0.582) |
| Cuba | 146401.976(103304.975-200303.387) | 4707.244(3321.552-6440.329) | 121081.713(80808.523-175849.585) | 6203.555(4140.180-9009.556) | -0.173(-0.364 - 0.048) | 0.237(0.024-0.449) |
| Cyprus | 14895.274(10579.798-20424.071) | 7832.891(5563.537-10740.288) | 20614.041(13526.278-29710.492) | 9520.322(6246.932-13721.397) | 0.384(0.093 - 0.754) | 0.240(0.104-0.376) |
| Czechia | 83016.626(58343.074-114056.601) | 3494.345(2455.783-4800.883) | 75365.294(48072.426-109321.179) | 4825.683(3078.105-6999.898) | -0.092(-0.302 - 0.175) | 0.252(0.015-0.489) |
| C么te d'Ivoire | 143837.723(101534.450-198834.207) | 3763.073(2656.337-5201.887) | 356033.547(237499.368-524064.781) | 4138.313(2760.545-6091.403) | 1.475(0.898 - 2.198) | 0.123(0.042-0.205) |
| Democratic People's Republic of Korea | 252015.486(178907.580-347611.052) | 4579.297(3250.876-6316.335) | 262100.772(168978.277-390866.955) | 4805.733(3098.291-7166.718) | 0.04(-0.184 - 0.371) | -0.022(-0.078-0.035) |
| Democratic Republic of the Congo | 477047.774(340482.368-652907.429) | 3972.396(2835.211-5436.787) | 1309123.993(860372.816-1937216.802) | 4394.290(2887.983-6502.587) | 1.744(1.081 - 2.553) | 0.175(0.052-0.299) |
| Denmark | 75136.776(53901.591-102259.231) | 6872.292(4930.042-9353.014) | 90168.888(60277.386-130031.096) | 8586.516(5740.037-12382.475) | 0.2(-0.064 - 0.527) | 0.254(0.078-0.431) |
| Djibouti | 5201.806(3782.914-7166.461) | 3537.797(2572.796-4873.978) | 15060.473(9656.901-21961.205) | 4232.253(2713.756-6171.478) | 1.895(1.198 - 2.683) | 0.211(0.071-0.351) |
| Dominica | 1042.099(737.234-1424.080) | 4567.378(3231.197-6241.549) | 1002.680(654.202-1481.873) | 6088.505(3972.463-8998.274) | -0.038(-0.28 - 0.207) | 0.327(0.128-0.527) |
| Dominican Republic | 113789.645(81585.461-158126.127) | 4777.017(3425.049-6638.312) | 164540.547(108836.519-240740.057) | 5758.455(3808.971-8425.223) | 0.446(0.103 - 0.83) | 0.273(0.067-0.480) |
| Ecuador | 172765.904(123720.338-234970.066) | 5298.850(3794.589-7206.695) | 365193.332(242987.723-531751.313) | 7514.469(4999.882-10941.680) | 1.114(0.637 - 1.724) | 0.482(0.164-0.801) |
| Egypt | 996863.480(709337.692-1367330.718) | 5795.377(4123.814-7949.130) | 2185074.901(1415328.654-3207772.001) | 7344.800(4757.414-10782.442) | 1.192(0.706 - 1.773) | 0.434(0.192-0.677) |
| El Salvador | 84747.411(60478.550-115978.972) | 4769.799(3403.886-6527.590) | 109792.131(71548.939-162306.325) | 6280.261(4092.698-9284.145) | 0.296(0.002 - 0.691) | 0.382(0.161-0.603) |
| Equa-rial Guinea | 5171.023(3651.516-7115.424) | 3966.612(2801.022-5458.133) | 24188.941(15771.017-35454.619) | 4417.785(2880.365-6475.310) | 3.678(2.593 - 4.894) | 0.100(-0.030-0.230) |
| Eritrea | 41785.694(29385.585-56217.156) | 3707.900(2607.563-4988.492) | 91308.478(58460.361-132335.234) | 4380.146(2804.394-6348.235) | 1.185(0.621 - 1.81) | 0.217(0.113-0.321) |
| Es-nia | 13106.178(9086.378-18184.376) | 4012.497(2781.823-5567.203) | 11662.583(7418.356-17189.562) | 5767.016(3668.293-8500.044) | -0.11(-0.31 - 0.16) | 0.291(0.048-0.534) |
| Eswatini | 10694.974(7530.588-14766.477) | 3881.927(2733.358-5359.750) | 22960.200(14709.052-33770.751) | 6242.018(3998.840-9181.002) | 1.147(0.638 - 1.736) | 0.464(0.142-0.788) |
| Ethiopia | 616822.675(492207.995-764920.552) | 3834.098(3059.508-4754.657) | 1661570.008(1275267.184-2117891.074) | 4404.859(3380.762-5614.577) | 1.694(1.337 - 2.088) | 0.107(-0.073-0.288) |
| Fiji | 10543.549(7383.612-14638.327) | 4432.429(3104.015-6153.843) | 13350.819(8423.734-19447.029) | 5576.623(3518.584-8123.003) | 0.266(-0.02 - 0.602) | 0.213(0.065-0.361) |
| Finland | 63365.701(47207.095-83027.147) | 6509.397(4849.465-8529.168) | 62239.126(39208.106-90941.380) | 6787.171(4275.640-9917.149) | -0.018(-0.313 - 0.284) | -0.111(-0.374-0.153) |
| France | 1059531.627(753135.353-1438827.237) | 8381.469(5957.709-11381.903) | 1291199.507(854101.649-1825564.536) | 10566.693(6989.648-14939.737) | 0.219(-0.045 - 0.499) | 0.427(0.213-0.641) |
| Gabon | 12471.376(8874.827-16974.318) | 4060.858(2889.770-5527.080) | 28839.821(18939.252-42277.421) | 5110.950(3356.386-7492.341) | 1.312(0.767 - 1.969) | 0.284(0.120-0.447) |
| Gambia | 11961.850(8387.868-16550.417) | 3821.067(2679.402-5286.828) | 36843.361(24061.952-54005.758) | 4523.416(2954.188-6630.516) | 2.08(1.319 - 2.888) | 0.203(0.066-0.341) |
| Georgia | 32536.420(22750.794-45458.385) | 2533.281(1771.373-3539.383) | 22225.074(14380.877-33248.460) | 3567.914(2308.642-5337.559) | -0.317(-0.479 - -0.133) | 0.277(0.034-0.520) |
| Germany | 1117902.227(832157.013-1482922.621) | 7504.944(5586.617-9955.479) | 1301298.094(876791.695-1877582.327) | 10307.791(6945.208-14872.631) | 0.164(-0.113 - 0.491) | 0.380(0.193-0.567) |
| Ghana | 169827.099(118315.713-236804.983) | 3606.981(2512.924-5029.534) | 441908.942(281653.762-667791.298) | 4207.706(2681.811-6358.481) | 1.602(0.978 - 2.279) | 0.208(0.090-0.325) |
| Greece | 174464.556(124842.732-238104.573) | 7444.775(5327.306-10160.431) | 155791.623(106557.613-220127.389) | 10091.342(6902.228-14258.666) | -0.107(-0.307 - 0.148) | 0.290(0.045-0.536) |
| Greenland | 667.469(470.942-940.559) | 4827.180(3405.884-6802.183) | 712.897(444.748-1029.828) | 6330.181(3949.151-9144.373) | 0.068(-0.186 - 0.364) | 0.468(0.191-0.745) |
| Grenada | 1188.427(832.284-1620.913) | 4523.653(3168.021-6169.874) | 1534.791(1005.057-2255.947) | 6040.297(3955.484-8878.466) | 0.291(-0.016 - 0.654) | 0.336(0.156-0.516) |
| Guam | 1723.780(1212.182-2363.849) | 4393.814(3089.781-6025.312) | 2025.967(1335.317-2965.285) | 5634.638(3713.797-8247.080) | 0.175(-0.093 - 0.521) | 0.276(0.092-0.461) |
| Guatemala | 124381.406(87683.247-169315.585) | 4663.094(3287.270-6347.689) | 310662.269(199895.744-447601.577) | 6282.445(4042.442-9051.735) | 1.498(0.931 - 2.175) | 0.430(0.207-0.654) |
| Guinea | 63886.617(44830.604-88338.372) | 3813.469(2675.993-5273.023) | 196604.242(124634.121-289876.608) | 4539.212(2877.561-6692.690) | 2.077(1.388 - 2.939) | 0.206(0.078-0.334) |
| Guinea-Bissau | 11890.413(8369.046-16385.185) | 3678.069(2588.802-5068.440) | 28965.404(18675.682-42963.232) | 4309.478(2778.571-6392.077) | 1.436(0.884 - 2.05) | 0.201(0.067-0.335) |
| Guyana | 12127.768(8683.976-16831.282) | 4677.632(3349.375-6491.759) | 13875.360(8988.021-20575.499) | 6640.912(4301.774-9847.679) | 0.144(-0.103 - 0.462) | 0.335(0.073-0.598) |
| Haiti | 91267.318(63587.431-124572.401) | 4702.086(3276.020-6417.962) | 221546.920(141368.107-334226.873) | 5922.893(3779.371-8935.307) | 1.427(0.869 - 2.084) | 0.301(0.136-0.466) |
| Honduras | 69690.160(49286.896-96200.581) | 4545.737(3214.877-6274.954) | 213964.340(140296.656-316479.541) | 6718.373(4405.245-9937.299) | 2.07(1.37 - 2.959) | 0.517(0.239-0.795) |
| Hungary | 83276.245(59203.540-114992.595) | 3612.566(2568.280-4988.437) | 73507.237(48151.523-106443.146) | 5060.800(3315.119-7328.359) | -0.117(-0.308 - 0.119) | 0.275(0.046-0.504) |
| Iceland | 4471.994(3239.107-6146.404) | 7074.035(5123.789-9722.704) | 5348.611(3346.238-7674.553) | 7844.488(4907.727-11255.809) | 0.196(-0.109 - 0.51) | 0.151(0.048-0.254) |
| India | 6498315.445(5084676.275-8171725.819) | 2521.019(1972.598-3170.217) | 12665986.662(9972263.432-16100147.716) | 3183.776(2506.670-4047.001) | 0.949(0.838 - 1.068) | 0.657(0.225-1.090) |
| Indonesia | 2017557.046(1620064.182-2505928.869) | 3393.567(2724.977-4215.017) | 3252883.933(2564042.938-4076564.241) | 4704.432(3708.207-5895.667) | 0.612(0.49 - 0.726) | 0.333(0.113-0.553) |
| Iran (Islamic Republic of) | 1577265.339(1277699.614-1920087.790) | 8558.958(6933.379-10419.269) | 1938991.709(1562328.438-2388168.930) | 10946.217(8819.835-13481.964) | 0.229(0.155 - 0.306) | 0.395(0.210-0.580) |
| Iraq | 404558.508(287562.909-553774.931) | 6604.438(4694.479-9040.403) | 1005389.770(667785.213-1510491.164) | 8032.425(5335.179-12067.864) | 1.485(0.909 - 2.172) | 0.473(0.301-0.644) |
| Ireland | 82528.136(57881.009-113583.470) | 8473.255(5942.707-11661.740) | 107043.687(70551.459-157003.505) | 10886.671(7175.300-15967.738) | 0.297(0.008 - 0.637) | 0.879(0.555-1.205) |
| Israel | 69317.444(49652.998-93715.897) | 5089.751(3645.856-6881.249) | 137826.499(89312.262-203592.306) | 6115.622(3962.953-9033.775) | 0.988(0.521 - 1.499) | 0.335(0.082-0.589) |
| Italy | 932451.019(746665.275-1147324.718) | 7449.856(5965.513-9166.598) | 811939.193(643252.152-997176.022) | 9334.149(7394.903-11463.654) | -0.129(-0.188 - -0.056) | 0.353(0.029-0.679) |
| Jamaica | 33837.245(23953.877-46729.183) | 4432.357(3137.730-6121.078) | 43453.679(28709.778-64876.063) | 6123.000(4045.456-9141.599) | 0.284(-0.008 - 0.645) | 0.368(0.153-0.583) |
| Japan | 968510.639(777384.153-1199756.517) | 3448.566(2768.024-4271.961) | 756450.825(603925.577-950404.243) | 4306.115(3437.861-5410.199) | -0.219(-0.261 - -0.179) | -0.097(-0.334-0.141) |
| Jordan | 88559.740(63787.086-120050.351) | 6532.829(4705.413-8855.812) | 302033.332(196220.996-430159.301) | 7974.360(5180.676-11357.174) | 2.411(1.625 - 3.243) | 0.234(0.097-0.373) |
| Kazakhstan | 100180.689(70627.934-138980.588) | 2293.410(1616.867-3181.646) | 118860.815(75360.594-172449.540) | 2897.456(1837.056-4203.782) | 0.186(-0.085 - 0.552) | 0.269(0.106-0.432) |
| Kenya | 274882.543(219289.709-339585.685) | 3466.095(2765.105-4281.961) | 736326.742(577179.489-918710.933) | 4267.303(3344.982-5324.291) | 1.679(1.559 - 1.811) | 0.286(0.119-0.454) |
| Kiribati | 993.041(700.036-1382.260) | 4443.698(3132.551-6185.395) | 1883.561(1177.525-2814.963) | 5328.339(3331.061-7963.150) | 0.897(0.442 - 1.423) | 0.181(0.063-0.300) |
| Kuwait | 29013.491(20374.937-39306.985) | 6295.601(4421.132-8529.173) | 57994.295(38094.790-83555.218) | 6956.638(4569.616-10022.768) | 0.999(0.554 - 1.522) | 0.250(0.151-0.349) |
| Kyrgyzstan | 29203.798(20701.836-40563.095) | 2260.331(1602.291-3139.523) | 54266.007(35264.272-79461.894) | 3058.324(1987.424-4478.313) | 0.858(0.421 - 1.378) | 0.370(0.130-0.610) |
| Lao People's Democratic Republic | 56109.382(39544.432-76004.932) | 4349.811(3065.634-5892.190) | 115672.697(73684.812-168503.394) | 5543.522(3531.286-8075.391) | 1.062(0.568 - 1.697) | 0.456(0.336-0.576) |
| Latvia | 22852.288(15974.774-30955.468) | 4234.544(2960.136-5736.069) | 18222.056(11274.890-26635.594) | 6717.162(4156.241-9818.629) | -0.203(-0.402 - 0.026) | 0.376(0.067-0.685) |
| Lebanon | 68780.626(49069.472-93470.907) | 7926.573(5654.975-10771.987) | 138700.041(91248.420-197098.265) | 11703.567(7699.580-16631.234) | 1.017(0.575 - 1.59) | 0.774(0.458-1.091) |
| Lesotho | 18487.014(13041.224-26044.941) | 3869.256(2729.475-5451.099) | 36885.016(23778.719-55047.627) | 6027.271(3885.610-8995.170) | 0.995(0.517 - 1.606) | 0.410(0.124-0.696) |
| Liberia | 28318.177(19974.910-39859.591) | 3894.932(2747.384-5482.359) | 78141.090(50232.672-115367.109) | 4345.092(2793.224-6415.071) | 1.759(1.115 - 2.495) | 0.165(0.070-0.260) |
| Libya | 100030.269(72069.825-137898.109) | 6798.454(4898.152-9372.103) | 151565.358(97094.106-218513.532) | 8693.074(5568.860-12532.905) | 0.515(0.205 - 0.889) | 0.296(0.110-0.483) |
| Lithuania | 39325.993(28002.028-53959.395) | 4806.560(3422.505-6595.105) | 31105.939(20065.756-45567.735) | 7537.700(4862.404-11042.133) | -0.209(-0.389 - -0.015) | 0.397(0.092-0.702) |
| Luxembourg | 5218.719(3741.785-7131.992) | 7224.218(5179.715-9872.743) | 9383.628(6248.620-13377.985) | 8770.344(5840.230-12503.643) | 0.798(0.364 - 1.274) | 0.234(0.052-0.416) |
| Madagascar | 145961.000(104853.923-201228.734) | 3811.319(2737.935-5254.465) | 458657.682(295603.488-672518.583) | 4773.368(3076.421-6999.073) | 2.142(1.358 - 3.054) | 0.284(0.121-0.448) |
| Malawi | 119595.674(84994.311-163161.948) | 3784.675(2689.695-5163.356) | 344730.608(222866.379-500980.425) | 4845.622(3132.667-7041.909) | 1.882(1.143 - 2.645) | 0.214(0.044-0.385) |
| Malaysia | 234572.657(166951.503-325114.416) | 4366.900(3108.037-6052.462) | 473094.329(311139.298-691704.921) | 5846.469(3845.040-8548.045) | 1.017(0.556 - 1.615) | 0.263(-0.068-0.595) |
| Maldives | 2580.716(1811.867-3612.789) | 3603.690(2530.075-5044.868) | 4447.065(2940.902-6745.016) | 4456.007(2946.815-6758.579) | 0.723(0.349 - 1.208) | 0.258(0.112-0.404) |
| Mali | 86657.339(60657.101-120541.197) | 3396.634(2377.525-4724.751) | 314599.843(202850.214-466952.088) | 3935.983(2537.875-5842.074) | 2.63(1.795 - 3.733) | 0.229(0.101-0.357) |
| Malta | 6388.931(4551.290-8694.260) | 7807.487(5561.829-10624.677) | 5880.774(3873.896-8680.727) | 9247.099(6091.426-13649.825) | -0.08(-0.293 - 0.169) | 0.227(0.083-0.372) |
| Marshall Islands | 667.198(470.436-904.804) | 4376.526(3085.856-5935.120) | 872.661(568.100-1296.114) | 5264.309(3427.052-7818.786) | 0.308(0.021 - 0.667) | 0.189(0.081-0.297) |
| Mauritania | 22044.327(15315.126-30745.046) | 3462.788(2405.745-4829.523) | 57189.571(36895.240-85995.877) | 3981.772(2568.798-5987.385) | 1.594(0.953 - 2.313) | 0.173(0.055-0.292) |
| Mauritius | 11946.126(8538.306-16446.335) | 3681.154(2631.047-5067.876) | 12497.047(8055.043-18219.347) | 4836.839(3117.612-7051.590) | 0.046(-0.196 - 0.326) | 0.272(0.087-0.456) |
| Mexico | 927113.769(742275.146-1149817.149) | 3190.482(2554.396-3956.872) | 1834140.881(1464535.058-2267382.259) | 5527.554(4413.672-6833.214) | 0.978(0.853 - 1.117) | 1.284(0.879-1.691) |
| Micronesia (Federated States of) | 1511.992(1057.324-2084.617) | 4400.791(3077.438-6067.470) | 1650.333(1072.666-2435.185) | 5265.441(3422.374-7769.536) | 0.091(-0.151 - 0.396) | 0.176(0.063-0.289) |
| Monaco | 317.870(224.311-427.799) | 7315.040(5161.992-9844.801) | 495.163(324.043-718.497) | 9399.603(6151.262-13639.115) | 0.558(0.191 - 1.045) | 0.274(0.081-0.467) |
| Mongolia | 16291.515(11422.759-22884.904) | 2299.798(1612.499-3230.557) | 19594.794(12327.295-28645.458) | 2580.458(1623.394-3772.349) | 0.203(-0.072 - 0.574) | -0.065(-0.232-0.103) |
| Montenegro | 5932.475(4181.336-8154.341) | 3740.998(2636.736-5142.098) | 6689.836(4342.512-9673.913) | 5704.895(3703.166-8249.628) | 0.128(-0.148 - 0.445) | 0.392(0.083-0.702) |
| Morocco | 524155.897(373762.604-703945.207) | 6474.391(4616.728-8695.154) | 785858.362(522277.558-1135828.519) | 8410.536(5589.600-12156.041) | 0.499(0.177 - 0.944) | 0.350(0.115-0.585) |
| Mozambique | 158777.734(111999.546-218334.956) | 3842.218(2710.246-5283.426) | 523860.494(344866.136-779737.599) | 4950.480(3258.984-7368.518) | 2.299(1.515 - 3.145) | 0.231(0.065-0.397) |
| Myanmar | 481878.466(342122.096-665850.069) | 3688.779(2618.944-5097.081) | 800447.010(528159.616-1166084.068) | 5252.097(3465.496-7651.209) | 0.661(0.281 - 1.141) | 0.331(0.099-0.564) |
| Namibia | 18199.498(12860.693-24977.791) | 3835.146(2710.110-5263.523) | 44908.420(29395.670-63663.811) | 6023.382(3942.721-8538.966) | 1.468(0.887 - 2.119) | 0.424(0.136-0.713) |
| Nauru | 133.965(94.278-185.489) | 4427.307(3115.716-6130.079) | 180.068(115.846-263.936) | 5257.912(3382.649-7706.827) | 0.344(0.018 - 0.715) | 0.162(0.051-0.273) |
| Nepal | 152050.416(107926.711-208978.417) | 2552.702(1811.930-3508.438) | 350939.282(225998.555-542879.571) | 3713.175(2391.218-5744.034) | 1.308(0.767 - 2.04) | 0.602(0.349-0.855) |
| Netherlands | 263360.050(198422.603-344482.364) | 8039.346(6057.061-10515.691) | 327625.123(217000.393-472797.039) | 10885.608(7210.012-15709.061) | 0.244(-0.05 - 0.574) | 0.365(0.165-0.566) |
| New Zealand | 73148.301(59839.316-90050.811) | 8697.733(7115.222-10707.533) | 87724.217(67168.559-114123.203) | 8664.405(6634.150-11271.798) | 0.199(-0.026 - 0.439) | -0.219(-0.286 - -0.151) |
| Nicaragua | 62786.001(44369.461-87021.667) | 4785.745(3381.979-6633.063) | 118749.683(78737.427-170972.468) | 6311.174(4184.647-9086.651) | 0.891(0.442 - 1.42) | 0.415(0.185-0.645) |
| Niger | 86215.156(61675.150-119453.551) | 3522.485(2519.856-4880.504) | 315110.972(200348.930-457408.298) | 3740.618(2378.301-5429.800) | 2.655(1.834 - 3.695) | 0.142(0.079-0.206) |
| Nigeria | 1008773.508(811972.416-1251233.345) | 3573.973(2876.729-4432.982) | 2954007.330(2350976.846-3697420.242) | 3790.084(3016.377-4743.906) | 1.928(1.784 - 2.09) | 0.074(-0.071-0.220) |
| Niue | 27.819(19.527-38.329) | 4341.568(3047.377-5981.766) | 20.779(13.465-30.740) | 5230.870(3389.600-7738.548) | -0.253(-0.427 - -0.045) | 0.169(0.057-0.280) |
| North Macedonia | 18919.903(13173.519-25954.062) | 3705.291(2579.914-5082.867) | 22240.129(14248.498-32780.785) | 6021.633(3857.857-8875.572) | 0.175(-0.104 - 0.485) | 0.519(0.163-0.877) |
| Northern Mariana Islands | 578.931(404.390-782.421) | 4568.923(3191.444-6174.860) | 661.797(431.819-996.960) | 5775.971(3768.793-8701.174) | 0.143(-0.132 - 0.463) | 0.105(-0.096-0.306) |
| Norway | 86092.948(69589.687-106179.156) | 9391.284(7591.057-11582.349) | 98983.457(78214.968-123822.640) | 10023.732(7920.575-12539.116) | 0.15(0.048 - 0.262) | -0.106(-0.266-0.054) |
| Oman | 34700.155(24773.531-47829.512) | 6384.015(4557.749-8799.509) | 75812.117(48795.349-111279.322) | 8173.592(5260.812-11997.446) | 1.185(0.685 - 1.696) | 0.301(0.119-0.484) |
| Pakistan | 937713.674(739640.128-1161404.172) | 2632.554(2076.479-3260.546) | 2363464.875(1755157.863-3114567.024) | 3255.866(2417.873-4290.570) | 1.52(1.185 - 1.946) | 0.370(0.211-0.530) |
| Palau | 206.738(145.893-281.522) | 4414.445(3115.236-6011.308) | 181.960(116.193-265.875) | 5179.120(3307.200-7567.586) | -0.12(-0.329 - 0.148) | 0.100(-0.012-0.213) |
| Palestine | 47161.598(33470.690-65151.626) | 6944.500(4928.527-9593.514) | 145172.066(93894.303-205754.726) | 8783.139(5680.754-12448.486) | 2.078(1.379 - 2.988) | 0.288(0.120-0.458) |
| Panama | 33098.548(23748.219-45775.953) | 4380.040(3142.680-6057.683) | 64088.293(39760.056-96366.632) | 5813.919(3606.927-8742.125) | 0.936(0.468 - 1.466) | 0.403(0.141-0.665) |
| Papua New Guinea | 57611.448(40569.152-79800.041) | 4399.652(3098.172-6094.143) | 151725.098(98110.502-228543.704) | 4968.248(3212.635-7483.678) | 1.634(0.975 - 2.428) | 0.107(0.027-0.187) |
| Paraguay | 79668.295(56727.241-109158.992) | 6458.075(4598.426-8848.651) | 177258.423(116789.212-253551.464) | 8905.602(5867.582-12738.625) | 1.225(0.728 - 1.826) | 0.401(0.187-0.616) |
| Peru | 385561.953(273937.460-522832.185) | 5471.884(3887.712-7420.019) | 779330.793(496156.726-1145137.201) | 8499.026(5410.859-12488.343) | 1.021(0.523 - 1.489) | 0.577(0.186-0.969) |
| Philippines | 793204.734(634263.474-976677.465) | 3873.432(3097.279-4769.378) | 1692127.401(1345646.541-2074210.864) | 5207.125(4140.911-6382.897) | 1.133(1.038 - 1.242) | 0.328(0.104-0.553) |
| Poland | 284878.933(226924.031-353331.622) | 3322.878(2646.882-4121.322) | 274603.553(218658.465-343595.306) | 4718.392(3757.112-5903.847) | -0.036(-0.103 - 0.029) | 0.394(0.159-0.630) |
| Portugal | 247670.240(178124.408-332818.647) | 9928.010(7140.224-13341.235) | 218931.868(143148.765-310762.163) | 13346.216(8726.433-18944.245) | -0.116(-0.3 - 0.114) | 0.425(0.177-0.673) |
| Puer- Rico | 45380.525(32271.676-62585.486) | 4618.497(3284.375-6369.492) | 35566.324(23045.718-52658.897) | 5815.501(3768.239-8610.333) | -0.216(-0.404 - -0.006) | 0.128(-0.071-0.328) |
| Qatar | 6111.675(4321.111-8200.336) | 6196.905(4381.371-8314.694) | 30573.863(19841.520-44036.084) | 6864.865(4455.092-9887.588) | 4.003(2.859 - 5.351) | -0.070(-0.231-0.090) |
| Republic of Korea | 545700.100(385785.682-745897.995) | 4152.066(2935.326-5675.311) | 361208.121(231423.791-527028.629) | 4686.174(3002.403-6837.465) | -0.338(-0.498 - -0.171) | 0.123(-0.070-0.316) |
| Republic of Moldova | 45111.391(31776.177-61194.726) | 4389.666(3092.053-5954.691) | 33687.275(21887.937-49051.498) | 6173.500(4011.164-8989.134) | -0.253(-0.43 - -0.032) | 0.383(0.138-0.629) |
| Romania | 205015.073(145719.264-283837.778) | 3474.823(2469.812-4810.798) | 159817.717(101951.156-227970.499) | 5151.839(3286.469-7348.792) | -0.22(-0.414 - 0.007) | 0.344(0.041-0.647) |
| Russian Federation | 1174357.083(948835.262-1447185.264) | 3733.920(3016.863-4601.389) | 1264560.287(1016284.325-1564370.677) | 5367.642(4313.792-6640.238) | 0.077(0.034 - 0.126) | 0.352(0.100-0.605) |
| Rwanda | 82298.069(58299.315-113038.296) | 3634.831(2574.886-4992.524) | 201711.446(134677.741-298602.953) | 4673.170(3120.160-6917.914) | 1.451(0.927 - 2.094) | 0.331(0.179-0.482) |
| Saint Kitts and Nevis | 583.461(415.371-796.913) | 4595.108(3271.291-6276.162) | 716.122(468.463-1045.590) | 5880.278(3846.681-8585.624) | 0.227(-0.072 - 0.556) | 0.331(0.157-0.505) |
| Saint Lucia | 2088.319(1467.945-2840.886) | 4604.651(3236.754-6264.027) | 2345.942(1550.522-3413.554) | 6463.586(4272.029-9405.088) | 0.123(-0.14 - 0.489) | 0.342(0.119-0.565) |
| Saint Vincent and the Grenadines | 1705.220(1219.874-2340.238) | 4559.461(3261.731-6257.391) | 1605.634(1049.057-2301.090) | 6047.070(3950.914-8666.265) | -0.058(-0.278 - 0.22) | 0.288(0.096-0.481) |
| Samoa | 2569.547(1805.156-3567.170) | 4355.295(3059.678-6046.233) | 3293.960(2181.350-4852.465) | 5262.730(3485.123-7752.740) | 0.282(-0.014 - 0.634) | 0.198(0.082-0.314) |
| San Marino | 411.464(290.929-558.541) | 7385.312(5221.839-10025.176) | 515.449(347.974-716.584) | 9532.130(6435.026-13251.674) | 0.253(-0.027 - 0.596) | 0.275(0.030-0.521) |
| Sao -me and Principe | 1476.272(1060.388-2046.513) | 3649.836(2621.632-5059.662) | 2915.924(1896.118-4285.867) | 4133.900(2688.122-6076.065) | 0.975(0.487 - 1.573) | 0.172(0.060-0.284) |
| Saudi Arabia | 316325.102(226685.612-435587.384) | 6272.164(4494.772-8636.923) | 594942.725(383205.928-866691.338) | 7225.234(4653.813-10525.462) | 0.881(0.441 - 1.45) | 0.295(0.154-0.437) |
| Senegal | 86781.493(60957.720-120005.732) | 3582.506(2516.451-4954.066) | 236266.723(149651.722-357943.925) | 4516.018(2860.453-6841.765) | 1.723(1.073 - 2.535) | 0.195(-0.001-0.391) |
| Serbia | 77906.560(55114.646-108096.275) | 3562.519(2520.288-4943.037) | 79895.500(51339.623-116765.715) | 4947.504(3179.190-7230.680) | 0.026(-0.217 - 0.325) | 0.309(0.075-0.544) |
| Seychelles | 813.281(577.323-1108.553) | 3643.829(2586.644-4966.770) | 1096.250(732.110-1601.000) | 5000.349(3339.388-7302.677) | 0.348(0.025 - 0.703) | 0.263(0.048-0.479) |
| Sierra Leone | 48960.281(34449.717-67306.269) | 4025.790(2832.649-5534.300) | 122234.405(78279.357-180614.257) | 4200.813(2690.216-6207.146) | 1.497(0.892 - 2.236) | 0.134(0.042-0.226) |
| Singapore | 30573.132(21364.916-41856.885) | 3707.407(2590.786-5075.715) | 30084.780(19416.831-44081.436) | 4155.081(2681.705-6088.193) | -0.016(-0.242 - 0.241) | 0.131(0.042-0.220) |
| Slovakia | 46086.019(32785.702-63503.628) | 3621.116(2576.070-4989.669) | 45236.479(29699.815-66740.648) | 5411.070(3552.614-7983.342) | -0.018(-0.238 - 0.254) | 0.346(0.089-0.603) |
| Slovenia | 16098.813(11330.041-22218.153) | 3632.907(2556.772-5013.816) | 14087.254(9035.631-21092.360) | 4761.915(3054.315-7129.852) | -0.125(-0.328 - 0.096) | 0.258(0.036-0.480) |
| Solomon Islands | 5060.673(3525.752-6966.042) | 4387.524(3056.772-6039.449) | 11105.106(7126.850-16712.471) | 5270.676(3382.527-7932.030) | 1.194(0.701 - 1.918) | 0.173(0.063-0.284) |
| Somalia | 87731.402(61998.502-120190.638) | 3444.572(2434.229-4719.010) | 342325.417(218036.868-509967.735) | 4705.809(2997.265-7010.320) | 2.902(1.971 - 4.113) | 0.270(0.060-0.481) |
| South Africa | 478879.009(382357.644-589127.115) | 4053.809(3236.736-4987.082) | 820009.381(640467.087-1029246.066) | 5721.469(4468.745-7181.381) | 0.712(0.541 - 0.89) | 0.348(0.089-0.608) |
| South Sudan | 84737.418(60583.437-114714.407) | 4269.454(3052.467-5779.830) | 162882.832(104126.095-234687.672) | 4788.968(3061.443-6900.124) | 0.922(0.464 - 1.42) | 0.166(0.053-0.280) |
| Spain | 579066.800(425329.616-779707.381) | 5945.442(4366.979-8005.475) | 585466.606(390304.980-834384.189) | 8367.986(5578.570-11925.728) | 0.011(-0.234 - 0.291) | 0.876(0.573-1.180) |
| Sri Lanka | 191342.647(136019.448-267278.180) | 3694.496(2626.301-5160.680) | 262588.431(168757.016-374688.770) | 5016.842(3224.161-7158.557) | 0.372(0.019 - 0.803) | 0.293(0.101-0.485) |
| Sudan | 417782.487(297150.946-567417.785) | 6467.846(4600.304-8784.405) | 1142315.651(759710.433-1731251.291) | 7957.965(5292.538-12060.798) | 1.734(1.059 - 2.568) | 0.264(0.080-0.448) |
| Suriname | 5540.666(3903.975-7691.714) | 4534.763(3195.212-6295.290) | 9347.266(5848.735-13788.132) | 6576.527(4115.039-9701.020) | 0.687(0.274 - 1.194) | 0.328(0.064-0.592) |
| Sweden | 110500.335(88248.126-136052.795) | 6674.543(5330.445-8217.987) | 141589.853(103932.539-188519.374) | 7979.789(5857.480-10624.666) | 0.281(0.054 - 0.572) | 0.257(0.084-0.431) |
| Switzerland | 118159.668(83734.115-160618.141) | 8886.680(6297.566-12079.942) | 139320.095(90702.745-198751.205) | 10246.863(6671.102-14617.966) | 0.179(-0.101 - 0.484) | 0.342(0.242-0.443) |
| Syrian Arab Republic | 297465.969(210361.785-404403.678) | 6865.016(4854.797-9332.959) | 411915.400(262033.438-606203.231) | 8906.134(5665.495-13106.883) | 0.385(0.072 - 0.739) | 0.371(0.199-0.543) |
| Taiwan (Province of China) | 316278.603(232404.441-409174.756) | 5531.860(4064.862-7156.657) | 171833.938(109071.583-254756.473) | 4885.156(3100.852-7242.603) | -0.457(-0.622 - -0.242) | -0.465(-0.596 - -0.335) |
| Tajikistan | 40330.966(28615.779-55313.615) | 2433.023(1726.288-3336.873) | 92544.711(59391.513-138192.794) | 3329.633(2136.826-4971.989) | 1.295(0.713 - 2.003) | 0.362(0.140-0.584) |
| Thailand | 613943.709(431209.425-841469.135) | 3426.381(2406.552-4696.186) | 490699.345(308670.015-736222.021) | 4175.051(2626.278-6264.048) | -0.201(-0.404 - 0.033) | 0.212(0.093-0.330) |
| Timor-Leste | 8277.996(5886.079-11469.052) | 3595.893(2556.864-4982.061) | 21534.067(14105.281-30775.614) | 4549.422(2979.970-6501.849) | 1.601(0.973 - 2.298) | 0.284(0.134-0.434) |
| -go | 46998.335(32848.931-65333.266) | 3934.162(2749.736-5468.953) | 114023.646(72257.826-167562.949) | 4354.119(2759.245-6398.576) | 1.426(0.879 - 2.072) | 0.125(0.038-0.212) |
| -kelau | 20.134(14.082-27.674) | 4401.017(3078.163-6049.185) | 19.385(12.743-28.001) | 5182.108(3406.441-7485.367) | -0.037(-0.249 - 0.24) | 0.130(0.019-0.241) |
| -nga | 1469.165(1037.220-2032.779) | 4397.001(3104.252-6083.817) | 1644.717(1054.057-2446.488) | 5291.851(3391.413-7871.535) | 0.119(-0.127 - 0.43) | 0.190(0.076-0.303) |
| Trinidad and -bago | 14794.515(10518.026-20480.564) | 4310.261(3064.341-5966.846) | 17588.666(11783.382-26581.881) | 6416.633(4298.771-9697.505) | 0.189(-0.09 - 0.515) | 0.421(0.175-0.668) |
| Tunisia | 178622.086(126011.410-241892.880) | 6679.974(4712.480-9046.128) | 246718.153(159207.246-363010.865) | 9827.511(6341.694-14459.792) | 0.381(0.075 - 0.764) | 0.407(0.153-0.661) |
| Turkey | 1013692.182(866362.446-1187236.480) | 5522.495(4719.857-6467.947) | 1475124.888(962376.831-2119985.571) | 7662.290(4998.906-11011.911) | 0.455(-0.014 - 0.989) | 0.344(0.128-0.561) |
| Turkmenistan | 27260.146(19123.674-38197.330) | 2378.051(1668.262-3332.161) | 42254.090(27621.738-62947.784) | 3167.210(2070.423-4718.332) | 0.55(0.176 - 0.972) | 0.145(0.026-0.265) |
| Tuvalu | 109.191(77.008-150.230) | 4437.326(3129.466-6105.078) | 182.565(120.222-270.158) | 5240.944(3451.262-7755.517) | 0.672(0.284 - 1.173) | 0.400(0.193-0.607) |
| Uganda | 213604.764(151966.534-292597.423) | 3780.890(2689.868-5179.091) | 723628.973(478816.768-1083585.679) | 4769.796(3156.118-7142.449) | 2.388(1.592 - 3.387) | 0.314(0.146-0.483) |
| Ukraine | 379716.029(301255.276-473882.771) | 3499.262(2776.209-4367.052) | 324487.881(233109.442-441834.172) | 4929.814(3541.538-6712.609) | -0.145(-0.34 - 0.08) | 0.404(0.159-0.650) |
| United Arab Emirates | 25181.659(18422.456-33396.658) | 5984.962(4378.492-7937.434) | 86872.222(56624.528-126914.302) | 7790.375(5077.875-11381.198) | 2.45(1.598 - 3.436) | 0.333(0.086-0.581) |
| United Kingdom | 706311.034(561820.881-876228.243) | 5959.213(4740.136-7392.820) | 927989.587(726257.611-1151696.539) | 7671.119(6003.525-9520.366) | 0.314(0.266 - 0.362) | 0.340(0.086-0.594) |
| United Republic of Tanzania | 320416.338(226374.253-435546.990) | 3758.908(2655.670-5109.543) | 890382.325(568601.121-1314778.112) | 4616.790(2948.297-6817.357) | 1.779(1.132 - 2.597) | 0.251(0.109-0.393) |
| United States of America | 2674257.665(2107597.748-3296421.390) | 4833.342(3809.185-5957.815) | 4537402.495(3613717.702-5617719.093) | 7001.977(5576.575-8669.087) | 0.697(0.606 - 0.795) | 0.488(0.182-0.795) |
| United States Virgin Islands | 1286.289(901.175-1756.312) | 4577.472(3206.982-6250.125) | 847.006(554.867-1197.816) | 6077.150(3981.095-8594.162) | -0.342(-0.495 - -0.156) | 0.325(0.120-0.530) |
| Uruguay | 46194.477(33006.259-63101.515) | 6026.812(4306.197-8232.607) | 59906.129(39714.942-83893.680) | 8326.113(5519.821-11660.047) | 0.297(-0.032 - 0.634) | 0.302(0.094-0.510) |
| Uzbekistan | 144205.811(100948.860-198281.523) | 2257.280(1580.171-3103.737) | 222413.388(144420.152-331326.122) | 2776.681(1802.988-4136.384) | 0.542(0.181 - 1.024) | 0.272(0.102-0.441) |
| Vanuatu | 2079.910(1458.097-2859.303) | 4395.255(3081.243-6042.264) | 4985.940(3278.678-7376.617) | 5316.669(3496.161-7865.926) | 1.397(0.857 - 2.064) | 0.197(0.081-0.312) |
| Venezuela (Bolivarian Republic of) | 271825.810(194446.008-372797.001) | 4536.207(3244.899-6221.206) | 316697.188(208536.256-475427.644) | 5342.797(3518.083-8020.638) | 0.165(-0.081 - 0.483) | 0.271(0.160-0.381) |
| Viet Nam | 493168.787(352825.203-678785.331) | 2289.196(1637.748-3150.793) | 609984.266(384061.794-893371.022) | 2871.980(1808.273-4206.246) | 0.237(-0.079 - 0.605) | 0.081(-0.302-0.465) |
| Yemen | 271546.755(190298.439-371246.160) | 6444.942(4516.579-8811.226) | 774242.380(495687.897-1128536.548) | 7035.915(4504.555-10255.557) | 1.851(1.169 - 2.682) | 0.154(0.084-0.224) |
| Zambia | 105776.611(75525.932-144205.971) | 3813.538(2722.918-5199.022) | 335582.665(219315.493-505129.300) | 5083.296(3322.119-7651.532) | 2.173(1.354 - 3.048) | 0.321(0.138-0.505) |
| Zimbabwe | 128706.303(90533.163-175789.548) | 3583.442(2520.625-4894.335) | 241327.127(155494.026-354334.611) | 4728.926(3046.983-6943.364) | 0.875(0.401 - 1.362) | 0.249(0.066-0.431) |
| location | Rate per 100 000 (95% UI) | | | | | |
|  | 1990 | | 2021 | | 1990-2021 | |
|  | DALYs cases | DALYs rate | DALYs cases | DALYs rate | Cases change | EAPCa |
| Afghanistan | 26519.957(16081.930-39971.817) | 785.262(476.190-1183.575) | 109264.821(63929.379-171809.484) | 1025.980(600.287-1613.265) | 3.12(2.151 - 4.259) | 0.336(0.130-0.542) |
| Albania | 4571.450(2677.124-7025.150) | 466.731(273.326-717.247) | 3606.078(2010.060-5804.355) | 682.370(380.359-1098.345) | -0.211(-0.395 - 0.021) | 0.471(0.187-0.757) |
| Algeria | 67051.796(40172.296-100677.493) | 787.639(471.893-1182.631) | 93582.325(53741.135-146554.153) | 920.606(528.673-1441.711) | 0.396(0.05 - 0.808) | 0.262(0.133-0.391) |
| American Samoa | 82.301(49.626-126.636) | 547.259(329.988-842.064) | 90.184(51.761-148.281) | 647.483(371.623-1064.600) | 0.096(-0.153 - 0.398) | 0.160(0.048-0.273) |
| Andorra | 108.124(65.268-166.406) | 890.057(537.269-1369.818) | 149.136(82.603-235.491) | 1144.853(634.107-1807.767) | 0.379(0.054 - 0.757) | 0.301(0.064-0.539) |
| Angola | 15684.705(9410.994-24161.115) | 489.483(293.695-754.012) | 61300.085(34424.998-96326.997) | 580.555(326.029-912.284) | 2.908(1.983 - 3.95) | 0.195(0.062-0.329) |
| Antigua and Barbuda | 99.072(59.206-153.325) | 570.073(340.679-882.253) | 145.664(80.115-231.515) | 739.712(406.840-1175.684) | 0.47(0.114 - 0.86) | 0.268(0.077-0.459) |
| Argentina | 64669.698(39082.591-98290.212) | 742.288(448.595-1128.189) | 105143.146(57907.455-165116.000) | 983.401(541.607-1544.326) | 0.626(0.25 - 1.109) | 0.140(-0.128-0.409) |
| Armenia | 2993.946(1786.949-4571.030) | 343.796(205.196-524.893) | 2742.927(1506.931-4424.980) | 509.002(279.640-821.139) | -0.084(-0.32 - 0.186) | 0.474(0.192-0.757) |
| Australia | 32744.663(19716.528-49304.434) | 824.778(496.623-1241.888) | 42327.496(24391.035-64494.100) | 895.877(516.245-1365.041) | 0.293(-0.024 - 0.647) | 0.362(0.208-0.517) |
| Austria | 15092.350(9072.732-23003.595) | 927.942(557.830-1414.359) | 16983.706(9528.588-27110.402) | 1202.109(674.435-1918.878) | 0.125(-0.137 - 0.425) | 0.502(0.325-0.680) |
| Azerbaijan | 6576.424(3963.484-10128.665) | 310.944(187.400-478.900) | 9592.876(5566.221-15467.040) | 434.845(252.317-701.120) | 0.459(0.125 - 0.901) | 0.348(0.102-0.594) |
| Bahamas | 460.112(277.666-701.062) | 569.350(343.589-867.505) | 758.893(433.490-1159.758) | 765.380(437.196-1169.671) | 0.649(0.247 - 1.169) | 0.336(0.096-0.577) |
| Bahrain | 1076.072(640.901-1659.112) | 832.460(495.808-1283.506) | 3410.823(1907.027-5467.263) | 1016.013(568.064-1628.584) | 2.17(1.449 - 3.144) | 0.157(-0.013-0.326) |
| Bangladesh | 111438.688(67755.500-169656.066) | 319.306(194.141-486.117) | 191906.231(107100.114-303507.995) | 419.054(233.868-662.752) | 0.722(0.257 - 1.265) | 0.500(0.302-0.699) |
| Barbados | 382.865(230.085-575.137) | 566.834(340.643-851.493) | 439.020(253.260-714.179) | 768.570(443.369-1250.278) | 0.147(-0.117 - 0.458) | 0.294(0.087-0.502) |
| Belarus | 11220.947(6593.076-17129.298) | 506.795(297.777-773.647) | 11707.402(6576.941-18772.383) | 823.396(462.564-1320.285) | 0.043(-0.194 - 0.385) | 0.436(0.084-0.789) |
| Belgium | 17029.821(9979.120-25999.137) | 845.960(495.715-1291.513) | 20513.759(11822.468-32286.095) | 1044.265(601.830-1643.543) | 0.205(-0.052 - 0.551) | 0.300(0.084-0.516) |
| Belize | 342.728(207.479-533.693) | 548.417(331.998-853.991) | 932.778(531.726-1482.461) | 706.346(402.649-1122.593) | 1.722(1.078 - 2.535) | 0.334(0.129-0.539) |
| Benin | 6531.700(3891.057-9918.409) | 452.694(269.678-687.417) | 22740.074(12586.740-36870.765) | 519.325(287.449-842.034) | 2.481(1.57 - 3.535) | 0.203(0.109-0.296) |
| Bermuda | 72.018(42.193-111.011) | 578.128(338.708-891.138) | 68.964(38.671-109.085) | 752.257(421.824-1189.891) | -0.042(-0.266 - 0.242) | 0.267(0.067-0.468) |
| Bhutan | 744.444(440.073-1139.566) | 327.183(193.412-500.840) | 721.964(408.193-1164.982) | 352.697(199.412-569.123) | -0.03(-0.275 - 0.276) | 0.329(0.266-0.391) |
| Bolivia (Plurinational State of) | 13473.925(8061.228-20318.625) | 672.455(402.319-1014.060) | 35347.531(21016.073-56109.282) | 1092.744(649.697-1734.579) | 1.623(1.047 - 2.398) | 0.659(0.249-1.070) |
| Bosnia and Herzegovina | 5257.042(3139.967-7931.777) | 457.214(273.088-689.840) | 3635.860(2018.941-5757.140) | 671.596(372.928-1063.427) | -0.308(-0.478 - -0.121) | 0.397(0.119-0.676) |
| Botswana | 2145.073(1287.841-3293.398) | 475.579(285.524-730.171) | 4519.642(2576.254-7404.451) | 691.296(394.047-1132.538) | 1.107(0.611 - 1.734) | 0.342(0.115-0.570) |
| Brazil | 327862.620(213950.257-465033.188) | 703.177(458.865-997.370) | 550575.310(358850.226-796338.707) | 1133.193(738.585-1639.022) | 0.679(0.532 - 0.819) | 0.672(0.369-0.976) |
| Brunei Darussalam | 355.712(213.834-536.277) | 466.909(280.679-703.919) | 587.965(331.432-928.579) | 554.412(312.518-875.588) | 0.653(0.245 - 1.148) | 0.155(0.030-0.280) |
| Bulgaria | 8459.301(4999.971-12908.640) | 459.015(271.306-700.443) | 7456.862(4259.010-12062.568) | 768.194(438.756-1242.666) | -0.119(-0.346 - 0.173) | 0.477(0.100-0.855) |
| Burkina Faso | 13012.571(7759.924-20061.799) | 443.954(264.748-684.455) | 36208.823(20969.701-56055.391) | 498.091(288.461-771.102) | 1.783(1.164 - 2.639) | 0.193(0.117-0.269) |
| Burundi | 7505.096(4416.046-11318.806) | 447.465(263.291-674.844) | 24539.566(13147.210-39069.357) | 568.437(304.543-905.006) | 2.27(1.474 - 3.263) | 0.380(0.219-0.541) |
| Cabo Verde | 513.226(307.716-777.501) | 449.523(269.522-680.996) | 866.069(496.744-1419.235) | 577.294(331.113-946.016) | 0.688(0.3 - 1.151) | 0.332(0.142-0.523) |
| Cambodia | 15298.608(9341.093-23343.151) | 477.045(291.276-727.892) | 29240.727(16250.258-46513.271) | 631.510(350.956-1004.543) | 0.911(0.452 - 1.486) | 0.349(0.189-0.510) |
| Cameroon | 14737.955(8662.365-21855.163) | 457.111(268.671-677.858) | 54244.263(30527.559-86618.925) | 522.315(293.948-834.049) | 2.681(1.668 - 3.678) | 0.184(0.090-0.278) |
| Canada | 31017.968(20917.485-42994.426) | 532.167(358.876-737.644) | 40422.540(24252.088-62873.243) | 625.239(375.121-972.497) | 0.303(-0.084 - 0.855) | 0.335(-0.125-0.797) |
| Central African Republic | 4245.378(2544.983-6400.545) | 504.076(302.179-759.970) | 10413.008(5716.047-16859.804) | 573.878(315.021-929.172) | 1.453(0.906 - 2.113) | 0.302(0.168-0.436) |
| Chad | 8395.436(5029.888-12877.894) | 459.863(275.514-705.391) | 29425.516(16152.504-46548.987) | 504.419(276.890-797.953) | 2.505(1.629 - 3.455) | 0.221(0.129-0.313) |
| Chile | 28521.596(19428.791-39489.403) | 759.075(517.079-1050.973) | 39066.643(22057.118-60449.067) | 995.496(562.059-1540.363) | 0.37(-0.06 - 0.876) | 0.502(0.284-0.719) |
| China | 1926168.183(1247632.842-2800636.448) | 533.626(345.644-775.889) | 1279255.241(821741.733-1895445.139) | 546.599(351.113-809.883) | -0.336(-0.38 - -0.294) | -0.277(-0.449 - -0.104 |
| Colombia | 67431.238(39354.372-103360.617) | 658.605(384.377-1009.530) | 102378.595(56038.225-164770.873) | 855.410(468.220-1376.720) | 0.518(0.133 - 0.993) | -0.198(-0.669-0.275) |
| Comoros | 678.877(402.923-1024.420) | 440.610(261.508-664.877) | 1251.340(713.913-2043.623) | 567.991(324.049-927.613) | 0.843(0.443 - 1.399) | 0.346(0.181-0.512) |
| Congo | 3896.559(2383.274-5879.404) | 478.242(292.510-721.605) | 9484.157(5463.705-15815.033) | 568.841(327.702-948.555) | 1.434(0.87 - 2.204) | 0.216(0.074-0.358) |
| Cook Islands | 32.152(19.108-49.552) | 545.098(323.955-840.079) | 27.036(15.381-43.332) | 662.133(376.697-1061.240) | -0.159(-0.365 - 0.078) | 0.207(0.088-0.326) |
| Costa Rica | 5338.109(3211.788-8102.758) | 579.522(348.682-879.662) | 8470.694(4725.456-13277.079) | 774.567(432.099-1214.067) | 0.587(0.184 - 1.006) | 0.418(0.210-0.626) |
| Croatia | 4701.432(2748.381-7137.126) | 455.070(266.026-690.830) | 4189.994(2381.203-6903.403) | 632.766(359.606-1042.541) | -0.109(-0.333 - 0.155) | 0.348(0.107-0.589) |
| Cuba | 17949.313(10770.303-27361.395) | 577.122(346.296-879.747) | 14863.730(8452.675-23781.699) | 761.535(433.068-1218.442) | -0.172(-0.368 - 0.049) | 0.248(0.038-0.458) |
| Cyprus | 1829.687(1091.547-2777.598) | 962.167(574.006-1460.639) | 2532.146(1416.087-4126.730) | 1169.438(654.001-1905.876) | 0.384(0.073 - 0.763) | 0.235(0.098-0.373) |
| Czechia | 10239.377(6122.420-15641.104) | 430.997(257.706-658.367) | 9296.291(5250.154-14679.031) | 595.247(336.170-939.907) | -0.092(-0.316 - 0.17) | 0.251(0.014-0.490) |
| C么te d'Ivoire | 17429.767(10345.227-26563.068) | 455.996(270.651-694.941) | 43600.773(24599.287-70280.236) | 506.788(285.927-816.894) | 1.502(0.928 - 2.218) | 0.155(0.072-0.237) |
| Democratic People's Republic of Korea | 31268.010(18805.276-48928.139) | 568.162(341.705-889.058) | 32567.288(18658.715-51455.926) | 597.136(342.116-943.467) | 0.042(-0.193 - 0.342) | -0.019(-0.074-0.037) |
| Democratic Republic of the Congo | 57431.364(34796.410-87217.759) | 478.233(289.751-726.266) | 159071.432(92849.392-256445.268) | 533.949(311.664-860.801) | 1.77(1.091 - 2.569) | 0.219(0.095-0.342) |
| Denmark | 9211.754(5478.393-13620.076) | 842.542(501.074-1245.743) | 11034.363(6521.810-17419.390) | 1050.770(621.053-1658.797) | 0.198(-0.088 - 0.54) | 0.257(0.083-0.431) |
| Djibouti | 638.941(378.825-981.181) | 434.550(257.643-667.310) | 1854.387(1029.103-2967.431) | 521.115(289.196-833.899) | 1.902(1.214 - 2.682) | 0.223(0.085-0.362) |
| Dominica | 127.905(75.747-192.796) | 560.590(331.989-844.997) | 122.496(70.165-194.526) | 743.824(426.060-1181.207) | -0.042(-0.279 - 0.209) | 0.321(0.124-0.519) |
| Dominican Republic | 13942.622(8237.205-21283.960) | 585.327(345.807-893.525) | 20167.243(11278.794-31816.321) | 705.797(394.726-1113.481) | 0.446(0.104 - 0.828) | 0.279(0.073-0.485) |
| Ecuador | 21230.206(12998.829-31638.751) | 651.145(398.683-970.382) | 44784.325(25762.225-70843.548) | 921.513(530.101-1457.726) | 1.109(0.633 - 1.731) | 0.486(0.173-0.801) |
| Egypt | 122061.962(73820.157-184117.857) | 709.621(429.162-1070.390) | 268562.501(149168.148-431781.784) | 902.732(501.406-1451.369) | 1.2(0.689 - 1.804) | 0.449(0.209-0.690) |
| El Salvador | 10324.063(6232.939-15786.353) | 581.064(350.806-888.496) | 13474.453(7568.561-20774.421) | 770.757(432.932-1188.326) | 0.305(0.004 - 0.674) | 0.407(0.192-0.623) |
| Equa-rial Guinea | 624.143(376.048-933.341) | 478.770(288.461-715.951) | 2958.725(1710.931-4824.530) | 540.371(312.479-881.136) | 3.74(2.604 - 5.001) | 0.157(0.031-0.282) |
| Eritrea | 5034.642(3054.721-7608.944) | 446.755(271.064-675.188) | 11219.091(6289.495-18115.598) | 538.189(301.712-869.021) | 1.228(0.665 - 1.86) | 0.282(0.183-0.381) |
| Es-nia | 1613.682(954.627-2437.385) | 494.034(292.262-746.213) | 1438.381(816.614-2299.568) | 711.263(403.807-1137.110) | -0.109(-0.318 - 0.144) | 0.297(0.054-0.541) |
| Eswatini | 1312.963(775.272-1998.660) | 476.563(281.398-725.449) | 2802.767(1620.209-4483.277) | 761.967(440.474-1218.835) | 1.135(0.617 - 1.737) | 0.455(0.134-0.777) |
| Ethiopia | 75210.154(48222.151-108397.486) | 467.498(299.743-673.786) | 203891.652(130804.054-301676.688) | 540.521(346.764-799.752) | 1.711(1.352 - 2.126) | 0.140(-0.038-0.318) |
| Fiji | 1295.874(776.553-1981.382) | 544.776(326.457-832.958) | 1646.372(910.883-2683.470) | 687.688(380.475-1120.882) | 0.27(-0.026 - 0.62) | 0.223(0.076-0.370) |
| Finland | 7776.462(4788.590-11769.506) | 798.856(491.920-1209.051) | 7622.960(4215.545-11690.688) | 831.283(459.705-1274.868) | -0.02(-0.307 - 0.293) | -0.118(-0.382-0.146) |
| France | 129638.551(77111.713-197392.798) | 1025.511(609.995-1561.484) | 157732.465(87897.243-246223.005) | 1290.823(719.318-2014.997) | 0.217(-0.053 - 0.509) | 0.432(0.221-0.644) |
| Gabon | 1512.803(903.869-2280.981) | 492.590(294.313-742.720) | 3503.561(2030.451-5529.471) | 620.896(359.833-979.925) | 1.316(0.773 - 1.958) | 0.303(0.142-0.464) |
| Gambia | 1461.445(878.826-2250.310) | 466.841(280.730-718.834) | 4507.829(2517.074-7004.784) | 553.445(309.032-860.007) | 2.085(1.238 - 2.897) | 0.203(0.067-0.340) |
| Georgia | 4009.847(2360.979-6081.556) | 312.206(183.825-473.509) | 2741.745(1502.294-4449.341) | 440.148(241.171-714.277) | -0.316(-0.487 - -0.119) | 0.277(0.034-0.521) |
| Germany | 136741.835(82938.634-208163.470) | 918.005(556.802-1397.488) | 159145.229(91346.301-254119.604) | 1260.615(723.569-2012.922) | 0.164(-0.103 - 0.494) | 0.375(0.188-0.563) |
| Ghana | 20662.917(12457.330-31872.435) | 438.863(264.583-676.943) | 54265.488(29285.173-84773.540) | 516.697(278.843-807.185) | 1.626(0.981 - 2.348) | 0.230(0.112-0.348) |
| Greece | 21365.396(12863.916-32644.005) | 911.707(548.931-1392.989) | 19052.540(11208.662-30392.645) | 1234.121(726.037-1968.672) | -0.108(-0.312 - 0.151) | 0.289(0.044-0.534) |
| Greenland | 80.838(48.411-124.089) | 584.628(350.108-897.421) | 86.640(46.732-137.828) | 769.317(414.953-1223.846) | 0.072(-0.185 - 0.376) | 0.476(0.203-0.750) |
| Grenada | 145.501(86.697-217.635) | 553.838(330.005-828.412) | 187.800(105.803-298.433) | 739.101(416.396-1174.508) | 0.291(-0.003 - 0.684) | 0.344(0.165-0.522) |
| Guam | 213.129(127.790-326.767) | 543.252(325.730-832.910) | 250.171(142.354-390.614) | 695.779(395.916-1086.380) | 0.174(-0.111 - 0.53) | 0.272(0.088-0.456) |
| Guatemala | 15116.965(8981.759-22997.371) | 566.739(336.729-862.178) | 38024.469(21930.860-61341.739) | 768.959(443.502-1240.499) | 1.515(0.931 - 2.165) | 0.455(0.234-0.676) |
| Guinea | 7779.642(4596.638-11797.830) | 464.376(274.379-704.227) | 24044.432(13825.331-39413.136) | 555.139(319.200-909.973) | 2.091(1.407 - 3.01) | 0.221(0.093-0.349) |
| Guinea-Bissau | 1447.363(866.389-2190.450) | 447.714(268.001-677.573) | 3540.086(2022.705-5716.286) | 526.695(300.938-850.470) | 1.446(0.877 - 2.12) | 0.223(0.091-0.356) |
| Guyana | 1477.244(887.069-2252.491) | 569.767(342.139-868.777) | 1691.595(932.532-2775.530) | 809.618(446.321-1328.401) | 0.145(-0.109 - 0.457) | 0.344(0.085-0.604) |
| Haiti | 11009.919(6498.848-16501.579) | 567.230(334.820-850.160) | 26745.451(15268.677-43315.938) | 715.020(408.197-1158.019) | 1.429(0.865 - 2.067) | 0.314(0.153-0.474) |
| Honduras | 8560.798(5212.656-13129.177) | 558.402(340.010-856.388) | 26291.127(14842.315-43100.749) | 825.528(466.041-1353.342) | 2.071(1.328 - 2.891) | 0.528(0.251-0.805) |
| Hungary | 10274.550(6118.812-15935.869) | 445.715(265.437-691.306) | 9053.818(4974.014-14662.199) | 623.334(342.449-1009.458) | -0.119(-0.313 - 0.124) | 0.279(0.051-0.508) |
| Iceland | 548.782(328.412-829.115) | 868.092(519.499-1311.538) | 655.442(360.773-1002.436) | 961.297(529.125-1470.214) | 0.194(-0.109 - 0.545) | 0.151(0.048-0.254) |
| India | 788920.175(505993.476-1146219.853) | 306.061(196.300-444.675) | 1546897.085(988389.521-2202785.728) | 388.835(248.446-553.701) | 0.961(0.85 - 1.085) | 0.682(0.253-1.113) |
| Indonesia | 248607.953(160155.857-365069.928) | 418.163(269.385-614.054) | 402007.152(265443.408-597784.158) | 581.397(383.893-864.536) | 0.617(0.506 - 0.736) | 0.348(0.131-0.566) |
| Iran (Islamic Republic of) | 193204.604(128031.029-277185.644) | 1048.416(694.755-1504.135) | 237674.788(152645.062-345754.667) | 1341.749(861.729-1951.894) | 0.23(0.152 - 0.309) | 0.395(0.211-0.580) |
| Iraq | 49423.748(29370.634-74104.094) | 806.845(479.477-1209.753) | 123398.672(69876.448-196847.372) | 985.877(558.268-1572.685) | 1.497(0.906 - 2.169) | 0.479(0.309-0.649) |
| Ireland | 10115.847(6055.741-15251.335) | 1038.605(621.750-1565.871) | 13070.127(7560.071-20790.526) | 1329.272(768.882-2114.460) | 0.292(0.001 - 0.634) | 0.876(0.551-1.202) |
| Israel | 8525.242(5075.156-13080.047) | 625.980(372.652-960.425) | 16936.594(9645.769-26990.704) | 751.509(428.001-1197.628) | 0.987(0.525 - 1.487) | 0.338(0.088-0.590) |
| Italy | 114100.341(74435.738-163740.658) | 911.609(594.707-1308.213) | 99359.149(65380.625-141902.923) | 1142.245(751.623-1631.333) | -0.129(-0.188 - -0.056) | 0.360(0.037-0.683) |
| Jamaica | 4155.367(2557.835-6350.230) | 544.313(335.052-831.820) | 5324.057(2900.755-8482.571) | 750.206(408.741-1195.268) | 0.281(-0.025 - 0.682) | 0.368(0.154-0.582) |
| Japan | 119428.792(76867.877-174993.989) | 425.249(273.703-623.099) | 93296.252(60844.269-134924.581) | 531.091(346.357-768.061) | -0.219(-0.266 - -0.172) | -0.093(-0.329-0.144) |
| Jordan | 10874.095(6519.619-16454.962) | 802.155(480.936-1213.841) | 37153.272(19949.063-59112.145) | 980.930(526.700-1560.694) | 2.417(1.604 - 3.315) | 0.245(0.108-0.383) |
| Kazakhstan | 12319.125(7327.018-18800.000) | 282.018(167.735-430.383) | 14646.402(8462.054-22953.872) | 357.034(206.278-559.544) | 0.189(-0.098 - 0.555) | 0.280(0.118-0.441) |
| Kenya | 33749.206(21949.849-48952.640) | 425.556(276.774-617.262) | 90422.541(58161.701-129967.702) | 524.034(337.070-753.214) | 1.679(1.558 - 1.822) | 0.300(0.135-0.466) |
| Kiribati | 121.524(71.947-188.193) | 543.801(321.951-842.133) | 231.304(129.872-354.154) | 654.329(367.389-1001.854) | 0.903(0.437 - 1.444) | 0.198(0.083-0.314) |
| Kuwait | 3572.933(2111.263-5436.346) | 775.286(458.120-1179.626) | 7143.106(3998.750-11638.797) | 856.843(479.665-1396.118) | 0.999(0.55 - 1.557) | 0.251(0.153-0.350) |
| Kyrgyzstan | 3589.576(2139.765-5523.404) | 277.828(165.615-427.503) | 6694.833(3645.430-10716.608) | 377.307(205.449-603.967) | 0.865(0.409 - 1.429) | 0.379(0.139-0.621) |
| Lao People's Democratic Republic | 6881.822(4082.273-10333.338) | 533.505(316.473-801.079) | 14236.664(7675.913-22023.002) | 682.281(367.862-1055.435) | 1.069(0.558 - 1.699) | 0.478(0.358-0.597) |
| Latvia | 2825.869(1709.306-4336.032) | 523.635(316.736-803.470) | 2246.798(1261.771-3579.037) | 828.233(465.124-1319.334) | -0.205(-0.4 - 0.035) | 0.378(0.071-0.685) |
| Lebanon | 8384.932(5039.253-12703.009) | 966.315(580.745-1463.949) | 16986.603(9593.028-26206.730) | 1433.337(809.464-2211.335) | 1.026(0.578 - 1.584) | 0.788(0.477-1.100) |
| Lesotho | 2263.064(1306.567-3495.041) | 473.650(273.459-731.498) | 4494.892(2626.997-7112.741) | 734.497(429.270-1162.272) | 0.986(0.505 - 1.689) | 0.410(0.128-0.693) |
| Liberia | 3385.246(2045.789-5134.588) | 465.613(281.381-706.220) | 9451.330(5259.858-15558.360) | 525.548(292.478-865.134) | 1.792(1.107 - 2.603) | 0.201(0.104-0.297) |
| Libya | 12285.436(7205.096-18333.113) | 834.967(489.687-1245.991) | 18576.617(10528.315-29802.135) | 1065.467(603.854-1709.310) | 0.512(0.209 - 0.904) | 0.292(0.106-0.477) |
| Lithuania | 4847.805(2828.469-7252.967) | 592.516(345.705-886.483) | 3832.881(2172.626-6115.324) | 928.797(526.478-1481.887) | -0.209(-0.4 - 0.015) | 0.399(0.096-0.702) |
| Luxembourg | 638.691(376.946-974.175) | 884.133(521.802-1348.541) | 1150.298(658.051-1829.072) | 1075.119(615.043-1709.530) | 0.801(0.368 - 1.264) | 0.240(0.060-0.422) |
| Madagascar | 17708.061(10286.979-26304.835) | 462.391(268.613-686.869) | 56080.063(30870.978-91995.923) | 583.640(321.282-957.425) | 2.167(1.425 - 3.158) | 0.319(0.158-0.479) |
| Malawi | 14418.676(8537.648-21646.812) | 456.287(270.179-685.026) | 42190.136(23535.862-67638.604) | 593.035(330.826-950.746) | 1.926(1.206 - 2.762) | 0.286(0.119-0.453) |
| Malaysia | 28880.598(16867.537-44050.541) | 537.653(314.013-820.063) | 58268.848(33370.856-92195.108) | 720.083(412.395-1139.341) | 1.018(0.522 - 1.617) | 0.257(-0.078-0.593) |
| Maldives | 316.336(186.413-472.008) | 441.729(260.305-659.107) | 547.494(301.382-870.956) | 548.595(301.988-872.707) | 0.731(0.325 - 1.212) | 0.277(0.132-0.422) |
| Mali | 10538.308(6362.045-16142.526) | 413.061(249.368-632.725) | 38387.936(21992.060-64341.366) | 480.274(275.144-804.980) | 2.643(1.723 - 3.696) | 0.254(0.127-0.381) |
| Malta | 784.921(468.359-1189.469) | 959.200(572.351-1453.571) | 720.783(421.440-1140.300) | 1133.380(662.684-1793.040) | -0.082(-0.297 - 0.183) | 0.217(0.072-0.362) |
| Marshall Islands | 82.104(48.754-125.180) | 538.563(319.807-821.125) | 107.619(60.674-174.085) | 649.208(366.016-1050.163) | 0.311(0.018 - 0.7) | 0.191(0.083-0.299) |
| Mauritania | 2694.558(1587.658-4129.704) | 423.269(249.394-648.706) | 7027.371(3925.084-11206.939) | 489.274(273.280-780.273) | 1.608(1.001 - 2.403) | 0.190(0.073-0.307) |
| Mauritius | 1466.425(909.385-2209.676) | 451.873(280.224-680.903) | 1533.053(873.191-2501.164) | 593.350(337.959-968.047) | 0.045(-0.201 - 0.337) | 0.272(0.089-0.455) |
| Mexico | 114220.766(74285.673-168210.036) | 393.069(255.640-578.862) | 225887.840(144537.815-320802.045) | 680.759(435.594-966.802) | 0.978(0.837 - 1.119) | 1.282(0.879-1.686) |
| Micronesia (Federated States of) | 186.315(111.533-281.856) | 542.286(324.626-820.369) | 203.552(113.599-312.736) | 649.439(362.442-997.794) | 0.093(-0.144 - 0.413) | 0.184(0.073-0.296) |
| Monaco | 38.923(23.206-58.879) | 895.715(534.030-1354.974) | 60.503(33.097-93.690) | 1148.522(628.267-1778.494) | 0.554(0.196 - 1.008) | 0.272(0.082-0.462) |
| Mongolia | 2000.318(1200.750-3115.953) | 282.376(169.504-439.865) | 2413.954(1383.357-3931.606) | 317.896(182.176-517.757) | 0.207(-0.072 - 0.598) | -0.052(-0.217-0.113) |
| Montenegro | 731.941(429.007-1107.665) | 461.560(270.530-698.489) | 822.466(457.296-1343.147) | 701.375(389.969-1145.397) | 0.124(-0.144 - 0.444) | 0.399(0.094-0.705) |
| Morocco | 64161.003(37840.361-96968.150) | 792.519(467.405-1197.754) | 96105.881(54717.639-151498.178) | 1028.559(585.608-1621.387) | 0.498(0.166 - 0.92) | 0.352(0.120-0.585) |
| Mozambique | 19130.963(11512.767-29029.884) | 462.945(278.594-702.486) | 63470.284(36774.911-100778.396) | 599.794(347.523-952.356) | 2.318(1.499 - 3.193) | 0.254(0.090-0.418) |
| Myanmar | 58764.672(35552.026-89838.170) | 449.843(272.151-687.711) | 98333.813(53890.396-159485.062) | 645.213(353.599-1046.454) | 0.673(0.266 - 1.176) | 0.360(0.130-0.590) |
| Namibia | 2228.047(1355.852-3341.017) | 469.512(285.716-704.046) | 5489.789(3136.883-8447.090) | 736.323(420.737-1132.974) | 1.464(0.891 - 2.157) | 0.438(0.155-0.723) |
| Nauru | 16.529(9.939-25.278) | 546.256(328.458-835.400) | 22.256(12.661-36.676) | 649.866(369.695-1070.919) | 0.346(-0.005 - 0.723) | 0.166(0.054-0.277) |
| Nepal | 18492.322(11102.729-27916.443) | 310.459(186.398-468.676) | 43037.473(23789.710-71302.732) | 455.366(251.711-754.431) | 1.327(0.773 - 2.055) | 0.631(0.381-0.882) |
| Netherlands | 32427.562(19455.702-48411.596) | 989.886(593.906-1477.815) | 40097.847(23138.273-63370.348) | 1332.283(768.788-2105.531) | 0.237(-0.063 - 0.578) | 0.357(0.160-0.555) |
| New Zealand | 8878.921(5812.205-12569.519) | 1055.752(691.103-1494.584) | 10679.503(6604.990-15866.105) | 1054.800(652.366-1567.074) | 0.203(-0.021 - 0.446) | -0.206(-0.274 - -0.139) |
| Nicaragua | 7677.384(4576.433-11546.839) | 585.194(348.830-880.136) | 14594.309(8476.219-23609.131) | 775.642(450.484-1254.751) | 0.901(0.455 - 1.455) | 0.435(0.210-0.661) |
| Niger | 10502.396(6224.039-16119.912) | 429.096(254.295-658.610) | 38786.648(20391.345-61393.680) | 460.428(242.061-728.792) | 2.693(1.768 - 3.736) | 0.179(0.116-0.242) |
| Nigeria | 122288.141(79686.210-177976.301) | 433.253(282.319-630.550) | 361036.578(235684.766-526758.824) | 463.221(302.391-675.848) | 1.952(1.796 - 2.112) | 0.111(-0.032-0.255) |
| Niue | 3.439(2.105-5.311) | 536.758(328.466-828.920) | 2.565(1.393-4.112) | 645.612(350.569-1035.246) | -0.254(-0.425 - -0.052) | 0.167(0.057-0.277) |
| North Macedonia | 2331.640(1394.692-3525.586) | 456.630(273.138-690.454) | 2734.037(1501.026-4314.685) | 740.255(406.411-1168.224) | 0.173(-0.103 - 0.512) | 0.523(0.171-0.877) |
| Northern Mariana Islands | 71.587(42.362-108.269) | 564.961(334.320-854.459) | 81.762(46.321-128.998) | 713.593(404.273-1125.857) | 0.142(-0.146 - 0.471) | 0.109(-0.089-0.307) |
| Norway | 10554.090(6882.365-15270.149) | 1151.273(750.750-1665.715) | 12137.632(7763.157-17531.437) | 1229.138(786.150-1775.352) | 0.15(0.043 - 0.266) | -0.102(-0.261-0.057) |
| Oman | 4265.723(2501.333-6438.646) | 784.793(460.187-1184.560) | 9336.352(5230.498-14945.165) | 1006.588(563.920-1611.295) | 1.189(0.673 - 1.735) | 0.300(0.117-0.483) |
| Pakistan | 114536.360(74497.708-164639.670) | 321.551(209.146-462.212) | 289182.916(178244.338-436077.782) | 398.373(245.546-600.733) | 1.525(1.153 - 1.974) | 0.384(0.225-0.543) |
| Palau | 25.539(15.168-38.997) | 545.327(323.876-832.708) | 22.528(12.485-35.557) | 641.224(355.372-1012.051) | -0.118(-0.341 - 0.155) | 0.104(-0.009-0.217) |
| Palestine | 5770.273(3492.738-8759.657) | 849.667(514.302-1289.851) | 17806.105(9868.551-29092.600) | 1077.297(597.063-1760.148) | 2.086(1.36 - 2.993) | 0.297(0.128-0.466) |
| Panama | 4070.178(2421.705-6157.317) | 538.620(320.472-814.818) | 7859.975(4343.608-12223.181) | 713.036(394.041-1108.855) | 0.931(0.448 - 1.481) | 0.404(0.147-0.662) |
| Papua New Guinea | 7039.127(4240.735-10801.120) | 537.562(323.855-824.856) | 18690.819(9913.758-30427.221) | 612.032(324.627-996.341) | 1.655(0.977 - 2.489) | 0.133(0.054-0.212) |
| Paraguay | 9755.666(5897.557-14706.842) | 790.814(478.068-1192.167) | 21649.950(12358.944-34742.329) | 1087.711(620.923-1745.482) | 1.219(0.716 - 1.813) | 0.401(0.188-0.614) |
| Peru | 47247.575(28783.879-71220.907) | 670.536(408.500-1010.765) | 95898.597(54715.048-152586.205) | 1045.826(596.697-1664.035) | 1.03(0.535 - 1.502) | 0.591(0.203-0.980) |
| Philippines | 97461.783(63622.040-141693.904) | 475.932(310.684-691.929) | 208376.240(135862.203-302385.215) | 641.229(418.084-930.519) | 1.138(1.041 - 1.251) | 0.341(0.119-0.563) |
| Poland | 35179.882(22936.326-51276.790) | 410.344(267.533-598.101) | 33884.162(21808.492-49718.583) | 582.217(374.726-854.293) | -0.037(-0.102 - 0.037) | 0.394(0.159-0.629) |
| Portugal | 30243.963(17832.043-44749.204) | 1212.347(714.808-1793.799) | 26718.127(14796.385-42324.424) | 1628.753(901.996-2580.122) | -0.117(-0.296 - 0.124) | 0.433(0.187-0.679) |
| Puer- Rico | 5571.789(3285.906-8389.741) | 567.056(334.415-853.846) | 4374.257(2396.697-6902.803) | 715.241(391.887-1128.687) | -0.215(-0.406 - 0.002) | 0.134(-0.063-0.332) |
| Qatar | 751.709(444.234-1148.122) | 762.192(450.429-1164.133) | 3758.897(2153.052-5825.620) | 843.999(483.433-1308.048) | 4(2.796 - 5.333) | -0.075(-0.234-0.086) |
| Republic of Korea | 67297.414(40096.640-103509.325) | 512.046(305.083-787.571) | 44581.451(24758.097-74233.174) | 578.382(321.202-963.072) | -0.338(-0.509 - -0.145) | 0.127(-0.069-0.323) |
| Republic of Moldova | 5571.282(3328.264-8420.924) | 542.126(323.864-819.417) | 4149.884(2371.533-6562.065) | 760.504(434.605-1202.558) | -0.255(-0.433 - -0.031) | 0.383(0.140-0.626) |
| Romania | 25171.439(15005.844-38467.516) | 426.634(254.336-651.990) | 19699.333(11135.857-30875.079) | 635.022(358.972-995.280) | -0.217(-0.408 - 0.026) | 0.355(0.054-0.657) |
| Russian Federation | 144571.774(94321.056-210700.220) | 459.672(299.898-669.931) | 156060.280(101647.065-224111.576) | 662.424(431.458-951.280) | 0.079(0.033 - 0.13) | 0.367(0.116-0.619) |
| Rwanda | 10035.603(5954.809-15047.036) | 443.239(263.004-664.577) | 24740.428(14023.197-41028.291) | 573.176(324.884-950.527) | 1.465(0.911 - 2.139) | 0.379(0.225-0.534) |
| Saint Kitts and Nevis | 71.484(42.716-108.374) | 562.983(336.416-853.512) | 87.699(49.266-139.710) | 720.118(404.533-1147.196) | 0.227(-0.082 - 0.579) | 0.338(0.165-0.510) |
| Saint Lucia | 255.317(153.813-396.038) | 562.962(339.152-873.246) | 286.697(155.378-463.749) | 789.913(428.101-1277.730) | 0.123(-0.134 - 0.495) | 0.342(0.121-0.564) |
| Saint Vincent and the Grenadines | 208.861(123.096-315.153) | 558.458(329.137-842.664) | 196.341(111.953-309.490) | 739.452(421.632-1165.587) | -0.06(-0.279 - 0.236) | 0.289(0.099-0.479) |
| Samoa | 317.348(186.837-480.328) | 537.895(316.683-814.140) | 406.433(227.950-663.633) | 649.355(364.193-1060.280) | 0.281(-0.024 - 0.669) | 0.195(0.081-0.310) |
| San Marino | 50.494(29.924-75.813) | 906.310(537.098-1360.762) | 63.153(35.602-101.082) | 1167.888(658.384-1869.302) | 0.251(-0.034 - 0.592) | 0.274(0.030-0.520) |
| Sao -me and Principe | 181.209(109.526-275.723) | 448.008(270.785-681.678) | 357.652(210.023-572.505) | 507.042(297.749-811.640) | 0.974(0.473 - 1.579) | 0.179(0.067-0.290) |
| Saudi Arabia | 38824.144(23037.102-58808.846) | 769.814(456.785-1166.075) | 73158.347(40940.102-117070.865) | 888.466(497.194-1421.758) | 0.884(0.434 - 1.459) | 0.294(0.154-0.435) |
| Senegal | 10520.162(6324.999-16343.259) | 434.292(261.108-674.681) | 28853.357(15749.964-47810.815) | 551.505(301.046-913.859) | 1.743(1.079 - 2.643) | 0.220(0.025-0.415) |
| Serbia | 9603.889(5724.402-14904.100) | 439.168(261.766-681.536) | 9857.193(5513.073-16130.126) | 610.404(341.395-998.853) | 0.026(-0.243 - 0.33) | 0.317(0.084-0.550) |
| Seychelles | 100.520(60.125-153.675) | 450.368(269.386-688.528) | 135.393(76.521-218.893) | 617.570(349.036-998.441) | 0.347(0.031 - 0.733) | 0.273(0.059-0.488) |
| Sierra Leone | 5955.549(3553.625-9018.888) | 489.699(292.199-741.584) | 14941.059(8588.589-24359.432) | 513.477(295.163-837.157) | 1.509(0.928 - 2.269) | 0.166(0.074-0.258) |
| Singapore | 3768.544(2236.755-5856.895) | 456.987(271.237-710.228) | 3715.485(2125.091-6051.934) | 513.155(293.501-835.847) | -0.014(-0.248 - 0.256) | 0.134(0.045-0.223) |
| Slovakia | 5682.140(3416.593-8556.529) | 446.463(268.452-672.312) | 5583.401(3095.449-8856.939) | 667.872(370.270-1059.444) | -0.017(-0.243 - 0.261) | 0.354(0.097-0.611) |
| Slovenia | 1979.820(1212.206-3001.848) | 446.772(273.550-677.406) | 1738.079(923.371-2852.304) | 587.523(312.127-964.164) | -0.122(-0.336 - 0.106) | 0.264(0.040-0.488) |
| Solomon Islands | 623.913(374.742-951.235) | 540.923(324.896-824.706) | 1367.376(764.315-2248.997) | 648.980(362.757-1067.413) | 1.192(0.663 - 1.939) | 0.178(0.069-0.287) |
| Somalia | 10662.237(6391.419-16299.786) | 418.628(250.944-639.974) | 41642.316(23741.453-65262.539) | 572.440(326.364-897.138) | 2.906(1.952 - 4.096) | 0.291(0.087-0.497) |
| South Africa | 58642.919(38068.012-85760.001) | 496.424(322.254-725.976) | 100265.108(64753.385-145349.034) | 699.582(451.805-1014.147) | 0.71(0.534 - 0.897) | 0.353(0.095-0.611) |
| South Sudan | 10283.697(6071.534-15351.361) | 518.139(305.911-773.471) | 19874.756(11078.979-31867.622) | 584.344(325.736-936.950) | 0.933(0.458 - 1.459) | 0.186(0.072-0.300) |
| Spain | 71063.949(42615.865-106505.136) | 729.634(437.549-1093.518) | 71620.876(41248.653-109127.148) | 1023.666(589.561-1559.738) | 0.008(-0.246 - 0.293) | 0.866(0.565-1.168) |
| Sri Lanka | 23480.321(14102.938-35931.248) | 453.364(272.303-693.770) | 32413.885(17919.426-54231.261) | 619.278(342.357-1036.107) | 0.38(0.023 - 0.798) | 0.310(0.118-0.503) |
| Sudan | 51043.072(30213.091-75759.852) | 790.217(467.740-1172.866) | 139881.605(81949.866-229561.396) | 974.488(570.905-1599.244) | 1.74(1.086 - 2.565) | 0.274(0.091-0.456) |
| Suriname | 676.058(396.977-1030.418) | 553.321(324.906-843.346) | 1139.018(629.003-1828.874) | 801.388(442.553-1286.754) | 0.685(0.254 - 1.233) | 0.331(0.071-0.593) |
| Sweden | 13566.543(8797.585-19534.965) | 819.459(531.400-1179.969) | 17360.741(10657.199-25602.126) | 978.425(600.624-1442.897) | 0.28(0.046 - 0.558) | 0.253(0.081-0.425) |
| Switzerland | 14430.444(8609.679-21580.650) | 1085.300(647.526-1623.061) | 17033.145(9713.230-26721.859) | 1252.772(714.399-1965.368) | 0.18(-0.105 - 0.482) | 0.353(0.254-0.452) |
| Syrian Arab Republic | 36524.977(21939.414-54615.946) | 842.935(506.325-1260.444) | 50158.079(28658.657-78462.190) | 1084.481(619.637-1696.452) | 0.373(0.047 - 0.729) | 0.350(0.183-0.516) |
| Taiwan (Province of China) | 39311.195(23985.304-58599.603) | 687.571(419.514-1024.934) | 21365.362(11972.665-36178.520) | 607.407(340.377-1028.538) | -0.457(-0.622 - -0.236) | -0.464(-0.594 - -0.334) |
| Tajikistan | 4967.782(2978.505-7470.276) | 299.689(179.683-450.655) | 11406.726(6318.325-18090.149) | 410.399(227.325-650.859) | 1.296(0.715 - 2.037) | 0.370(0.149-0.592) |
| Thailand | 75616.100(45106.810-113127.737) | 422.009(251.738-631.359) | 60567.881(35166.402-99681.893) | 515.334(299.209-848.130) | -0.199(-0.398 - 0.042) | 0.230(0.113-0.346) |
| Timor-Leste | 1011.602(607.240-1521.022) | 439.431(263.780-660.719) | 2660.086(1463.074-4290.842) | 561.986(309.098-906.510) | 1.63(0.988 - 2.379) | 0.330(0.182-0.479) |
| -go | 5709.108(3395.293-8778.606) | 477.901(284.215-734.844) | 13935.533(7859.466-21650.153) | 532.144(300.122-826.735) | 1.441(0.866 - 2.111) | 0.146(0.060-0.231) |
| -kelau | 2.487(1.454-3.823) | 543.557(317.863-835.598) | 2.396(1.334-3.916) | 640.458(356.630-1046.914) | -0.037(-0.251 - 0.261) | 0.132(0.022-0.243) |
| -nga | 182.030(109.809-273.949) | 544.790(328.642-819.890) | 203.038(114.266-322.295) | 653.271(367.649-1036.978) | 0.115(-0.142 - 0.448) | 0.180(0.067-0.294) |
| Trinidad and -bago | 1810.473(1080.470-2780.591) | 527.467(314.786-810.103) | 2157.818(1250.574-3505.076) | 787.207(456.230-1278.709) | 0.192(-0.069 - 0.519) | 0.426(0.179-0.673) |
| Tunisia | 21986.442(13182.657-33479.889) | 822.232(492.995-1252.056) | 30215.297(17263.294-46396.225) | 1203.564(687.648-1848.098) | 0.374(0.049 - 0.76) | 0.403(0.151-0.655) |
| Turkey | 124665.153(84974.260-171004.710) | 679.163(462.931-931.617) | 181406.664(97635.124-280209.990) | 942.287(507.149-1455.504) | 0.455(-0.013 - 0.99) | 0.354(0.138-0.571) |
| Turkmenistan | 3352.745(2005.095-5164.947) | 292.478(174.915-450.566) | 5203.270(2826.585-8532.311) | 390.018(211.870-639.550) | 0.552(0.15 - 1.01) | 0.164(0.047-0.281) |
| Tuvalu | 13.464(8.026-20.516) | 547.150(326.147-833.720) | 22.518(12.399-36.723) | 646.421(355.928-1054.214) | 0.672(0.29 - 1.194) | 0.407(0.202-0.613) |
| Uganda | 25895.465(15402.235-38633.911) | 458.360(272.626-683.836) | 88793.770(50926.862-137257.444) | 585.284(335.684-904.732) | 2.429(1.63 - 3.396) | 0.358(0.189-0.528) |
| Ukraine | 46780.782(29685.548-69336.525) | 431.107(273.566-638.969) | 40030.936(24198.198-62469.758) | 608.174(367.633-949.078) | -0.144(-0.345 - 0.096) | 0.407(0.162-0.652) |
| United Arab Emirates | 3097.007(1859.800-4590.074) | 736.070(442.022-1090.930) | 10670.254(5995.997-17096.853) | 956.868(537.699-1533.182) | 2.445(1.603 - 3.426) | 0.335(0.086-0.584) |
| United Kingdom | 86238.480(55791.747-126627.267) | 727.602(470.720-1068.366) | 113384.620(73004.808-164361.895) | 937.281(603.486-1358.679) | 0.315(0.27 - 0.363) | 0.341(0.089-0.593) |
| United Republic of Tanzania | 38503.328(23348.729-58337.437) | 451.695(273.912-684.375) | 108758.516(60563.025-182824.087) | 563.932(314.030-947.975) | 1.825(1.141 - 2.731) | 0.318(0.179-0.456) |
| United States of America | 326703.618(212298.367-469381.707) | 590.471(383.699-848.341) | 548938.511(357515.618-799305.175) | 847.105(551.707-1233.463) | 0.68(0.579 - 0.785) | 0.465(0.162-0.769) |
| United States Virgin Islands | 158.082(97.578-245.225) | 562.563(347.246-872.673) | 104.099(58.078-164.085) | 746.895(416.700-1177.287) | -0.341(-0.493 - -0.144) | 0.331(0.128-0.535) |
| Uruguay | 5694.548(3434.326-8612.049) | 742.945(448.063-1123.580) | 7350.815(4085.961-11453.316) | 1021.660(567.891-1591.851) | 0.291(-0.04 - 0.623) | 0.296(0.090-0.503) |
| Uzbekistan | 17699.349(10406.771-27133.288) | 277.051(162.899-424.722) | 27409.397(15269.147-45080.501) | 342.188(190.625-562.800) | 0.549(0.176 - 1.04) | 0.283(0.113-0.453) |
| Vanuatu | 256.061(152.218-388.620) | 541.106(321.666-821.231) | 615.618(336.360-971.240) | 656.454(358.671-1035.665) | 1.404(0.827 - 2.079) | 0.203(0.087-0.320) |
| Venezuela (Bolivarian Republic of) | 33420.466(20255.936-50597.334) | 557.718(338.029-844.364) | 38945.144(22261.324-61585.738) | 657.019(375.557-1038.974) | 0.165(-0.104 - 0.499) | 0.278(0.167-0.388) |
| Viet Nam | 60867.236(35884.533-91758.495) | 282.534(166.569-425.926) | 75559.497(42529.199-125131.556) | 355.756(200.240-589.155) | 0.241(-0.089 - 0.64) | 0.093(-0.291-0.478) |
| Yemen | 32941.003(19637.032-50847.448) | 781.828(466.069-1206.823) | 93828.852(52781.053-147077.051) | 852.668(479.647-1336.560) | 1.848(1.163 - 2.692) | 0.157(0.089-0.225) |
| Zambia | 12806.269(7481.703-19665.207) | 461.701(269.736-708.985) | 40860.164(22571.761-66386.775) | 618.936(341.910-1005.605) | 2.191(1.333 - 3.1) | 0.360(0.177-0.543) |
| Zimbabwe | 15780.041(9517.264-24269.947) | 439.348(264.980-675.724) | 29604.606(16453.097-46827.179) | 580.117(322.407-917.602) | 0.876(0.413 - 1.422) | 0.260(0.079-0.442) |

a EAPC is expressed as 95% CIs.
